# Supplementary material for: Carbene-activated stannylenes to access selective C(sp3)–H bond scission at the steric limit
Source: Nat Commun. 2025 Mar 18;16:2657. doi: 10.1038/s41467-025-57907-2 (PMC11920205; doi:10.1038/s41467-025-57907-2)
Supplement: Supplementary file 1 — Supplementary Information [file 41467_2025_57907_MOESM1_ESM.pdf]

Supplementary Information

Carbene-activated stannylenes to access selective C–H bond scission at the steric limit

Jennifer Klaucke,[1] Navutheya Sinthathurai,[1] Christopher Golz,[2] Oliver P. E. Townrow,*[3] Malte Fischer*[1]

[1] Institut für Anorganische Chemie, Georg-August-Universität Göttingen, Tammannstraße 4, D-37077 Göttingen (Germany)
[2] Institut für Organische und Biomolekulare Chemie, Georg-August-Universität Göttingen, Tammannstraße 2, D-37077 Göttingen
[3] Insitute of Nanotechnology, Karlsruher Institut für Technologie, Hermann-von-Helmholtz-Platz 1, D-76344, Eggenstein-Leopoldshafen (Germany

Corresponding authors: oliver.townrow@kit.edu; malte.fischer@uni-goettingen.de

Table of Contents

General Considerations S2

Synthesis and Characterization of Compounds S3

Crystallographic Details S35

Computational Details S44

References S56

General Considerations

Materials and Synthetic Methods

All manipulations of air- and moisture-sensitive materials were carried out using standard Schlenk-line and glovebox techniques (MBraun glovebox with oxygen and water concentrations below 0.1 ppm as monitored by an O_2/H_2O Combi-Analyzer) under an inert atmosphere of argon. Solvents were purified by a Solvent Purification System, degassed by sparging with argon and stored over 3 Å molecular sieves. IMe_4,[1,2] $I^iPr_2Me_2$,[2] $^{Mes}TerSn\{N(SiMe_3)_2\}$ (**1a**),[3] $^{Dipp}TerSn\{N(SiMe_3)_2\}$ (**1b**),[3] and $^{Tipp}TerSn\{N(SiMe_3)_2\}$ (**1c**)[4] were synthesized according to literature procedures.

Analytical Methods

NMR spectra were measured in benzene-d_6 (C_6D_6) or toluene-d_8 (C_7D_8) (dried over CaH_2, distilled by trap-to-trap transfer in vacuo, degassed by three freeze-pump-thaw cycles and transferred to the glovebox). NMR samples were prepared under argon in NMR tubes with J. Young Teflon valves. NMR spectra were measured on Bruker Avance 400 MHz, 500 MHz, and 600 MHz spectrometers. ^1H and ^{13}C NMR spectra were referenced internally to residual protio-solvent (^1H) or solvent (^{13}C) resonances (C_6D_6: δ_H = 7.16 ppm; δ_C = 128.06 ppm; C_7D_8: 2.08 ppm; δ_C = 20.43 ppm). ^{119}Sn NMR spectra were referenced with respect to $SnMe_4$. LIFDI-(JEOL AccuTOF JMS-T100GCV; inert conditions) and ESI- (Bruker Daltronik micro TOF) MS were measured by the Zentrale Massenabteilung (Fakultät für Chemie, Georg-August-Universität Göttingen). Elemental analyses were obtained from the Analytische Labor (Georg-August-Universität Göttingen) using an Elementar Vario EL 3 analyzer.

Synthesis and Characterization of Compounds

Additional analytical data of the starting materials:

^{Mes}TerSn{N(SiMe$_3$)$_2$} (1a): ^{119}Sn{^{1}H} NMR (149 MHz, C_6D_6, 298 K): δ = 1192.3 ppm.

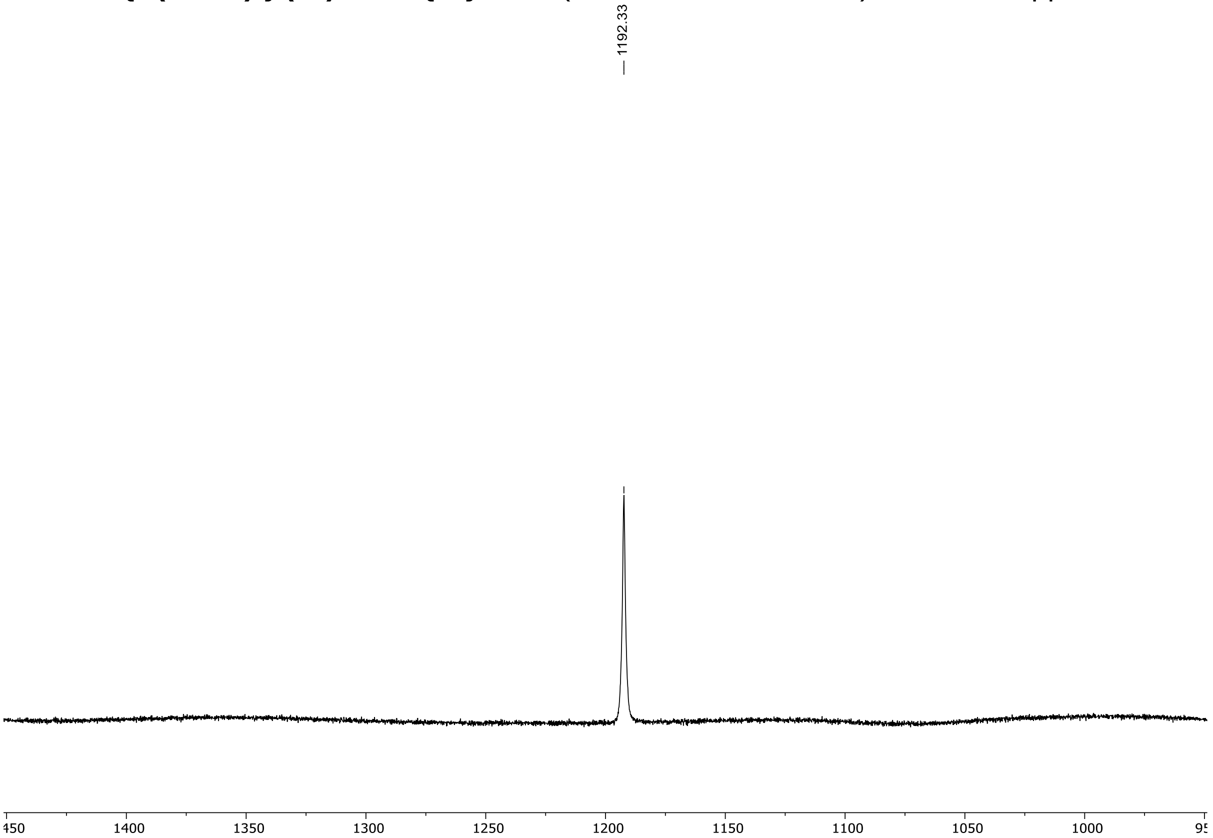

Figure S1. ^{119}Sn{^{1}H} NMR spectrum of **1a** (149 MHz, C_6D_6, 298 K).

^{Dipp}**TerSn{N(SiMe$_3$)$_2$} (1b): ^{119}Sn{^{1}H} NMR** (149 MHz, C_6D_6, 298 K): δ = 1200.4 ppm.

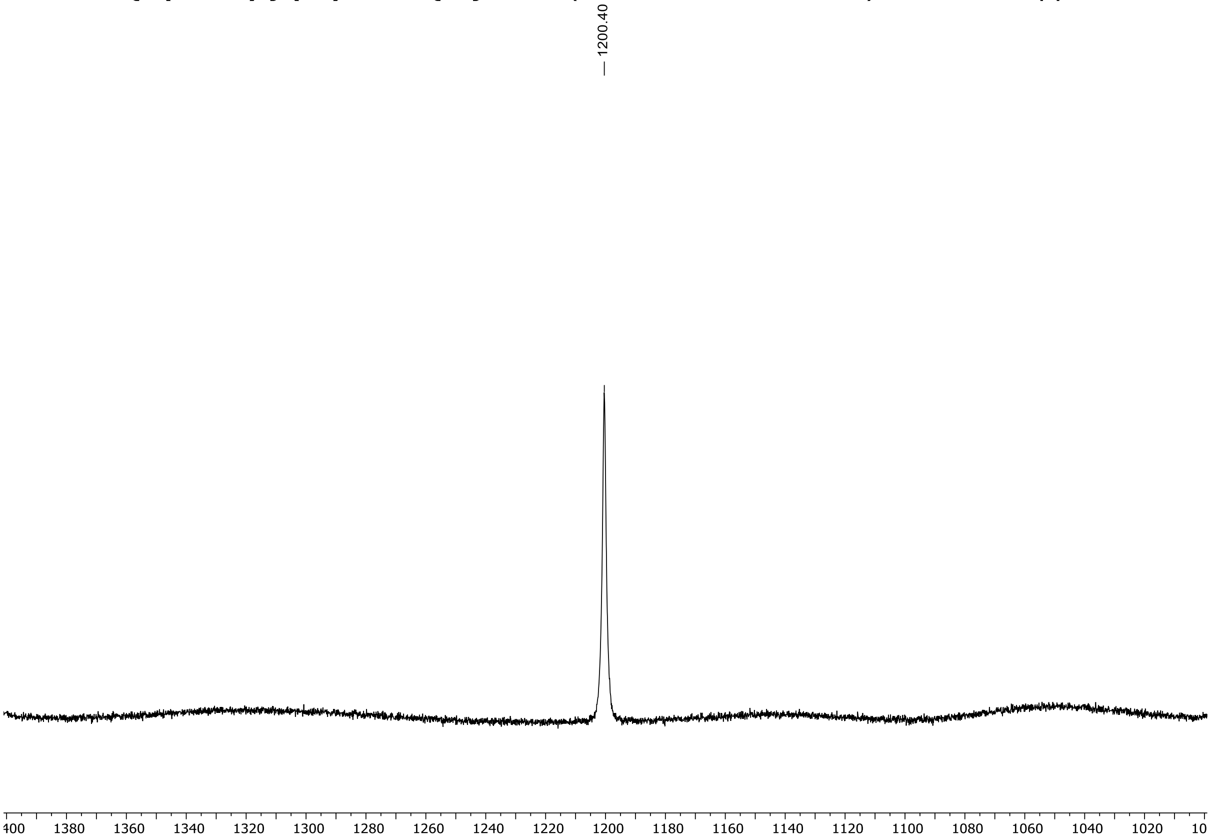

Figure S2. ^{119}Sn{^{1}H} NMR spectrum of **1b** (149 MHz, C_6D_6, 298 K).

IMe$_4$: ^{1}H NMR (400 MHz, C_6D_6, 298 K): δ = 1.59 (s, 6H, C_qCH_3), 3.37 (s, 6H, NCH_3) ppm; **^{13}C{^{1}H} NMR** (126 MHz, C_6D_6, 298 K): δ = 8.8 ($C_q\underline{C}H_3$), 35.2 (NCH_3), 122.6 ($\underline{C}_qCH_3$), 213.3 (C_{NHC}) ppm.

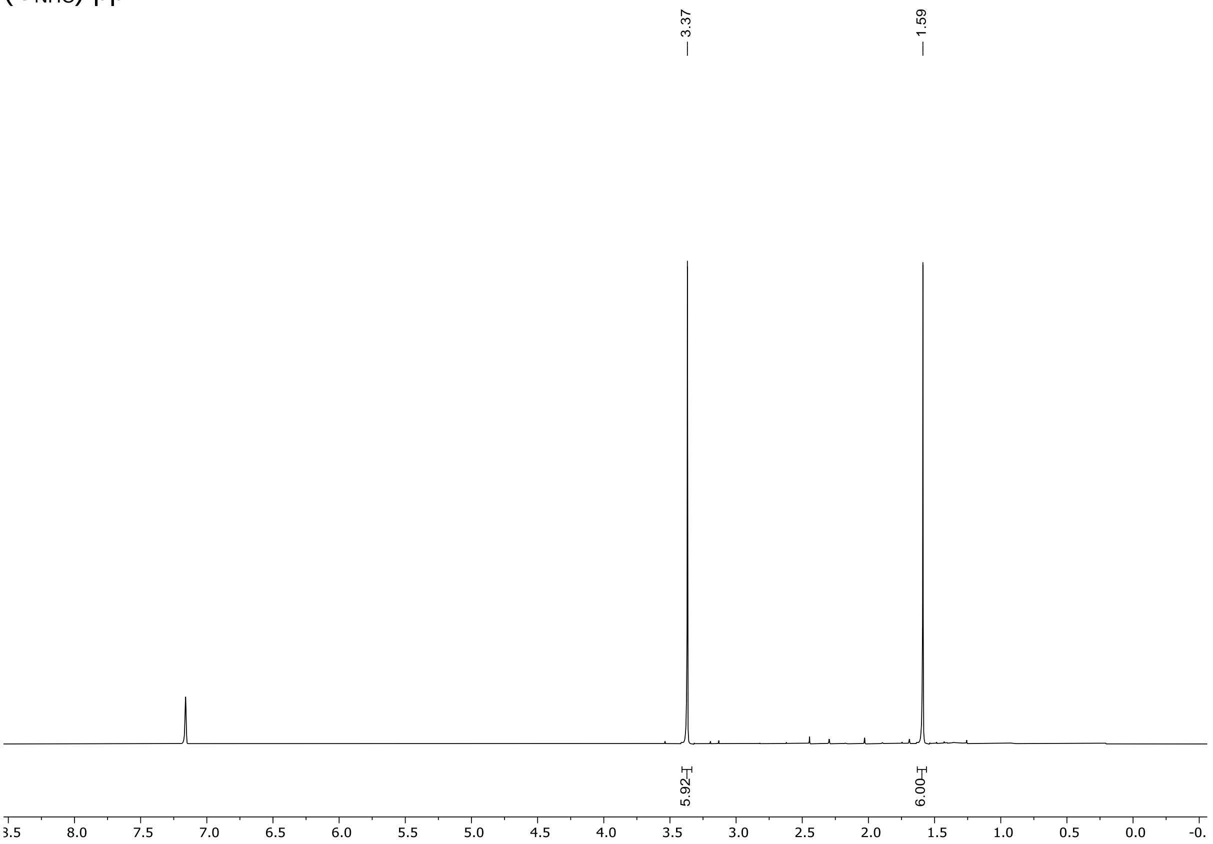

Figure S3. ^{1}H NMR spectrum of IMe$_4$ (400 MHz, C_6D_6, 298 K).

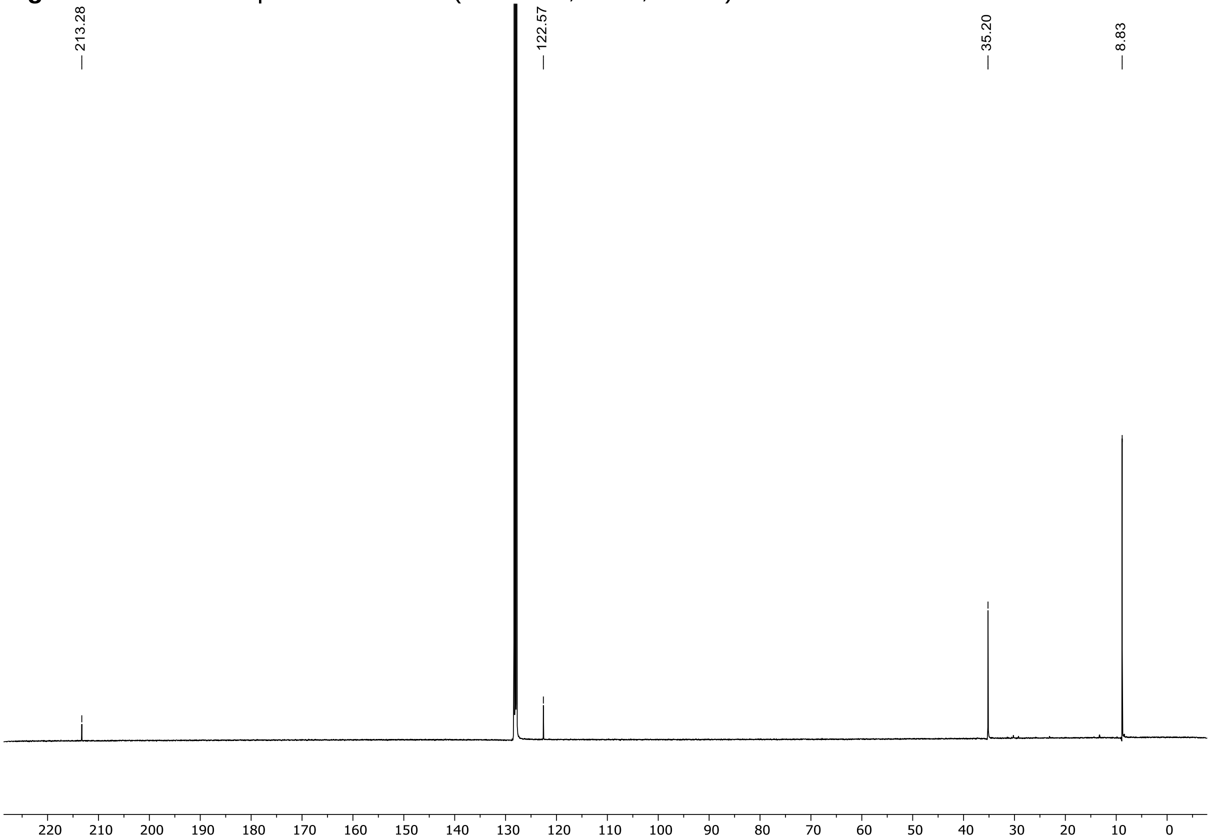

Figure S4. ^{13}C{^{1}H} NMR spectrum of IMe$_4$ (126 MHz, C_6D_6, 298 K).

I^{i}Pr$_2$Me$_2$: ^{1}H NMR (400 MHz, C_6D_6, 298 K): δ = 1.50 (d, $^{3}J_{H,H}$ = 6.6 Hz, 12H, CH(CH_3)$_2$), 1.72 (s, 6H, CH_3), 3.95 (hept, $^{3}J_{H,H}$ = 6.6 Hz, 2H, CH(CH$_3$)$_2$) ppm; **^{13}C{^{1}H} NMR** (126 MHz, C_6D_6, 298 K): δ = 8.9 (C$_q$<u>C</u>H$_3$), 24.7 (CH(<u>C</u>H$_3$)$_2$), 48.5 (<u>C</u>H(CH$_3$)$_2$), 121.5 (<u>C</u>$_q$CH$_3$), 207.6 (C$_{NHC}$) ppm.

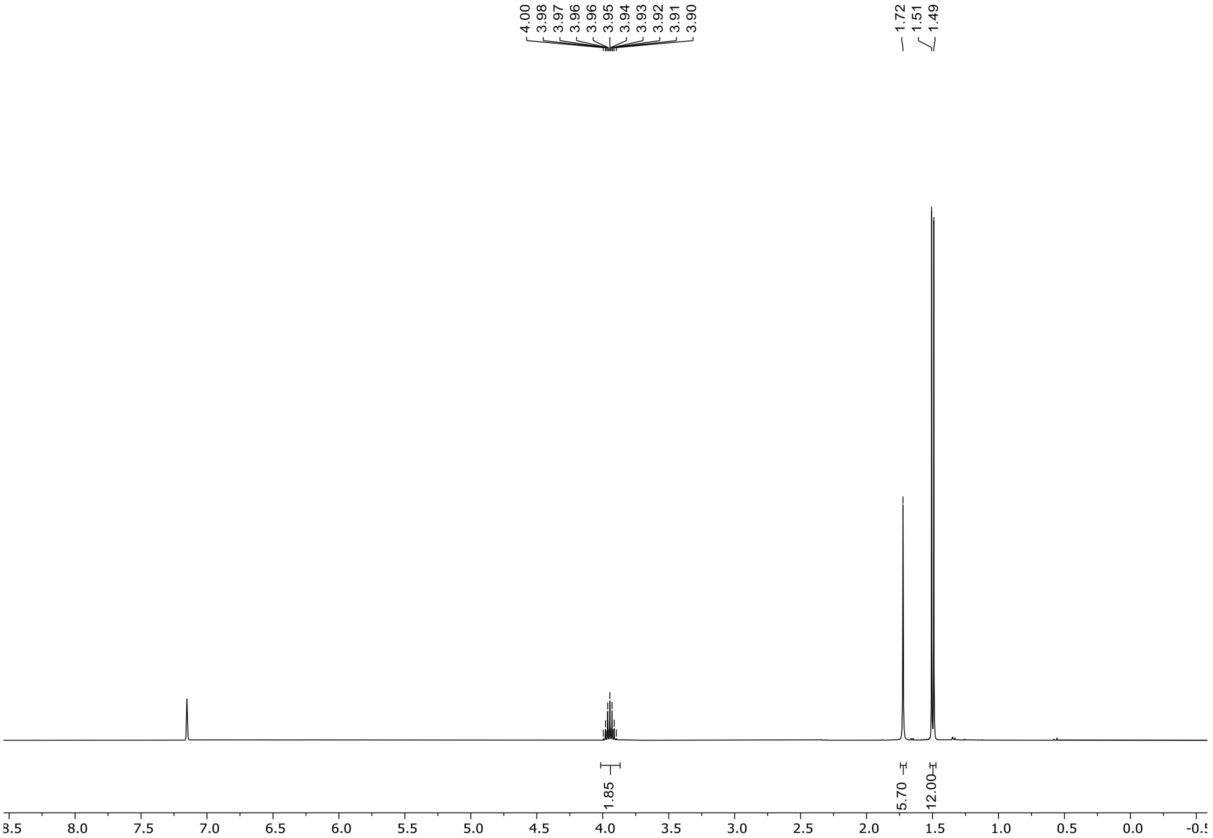

Figure S5. ^{1}H NMR spectrum of I^{i}Pr$_2$Me$_2$ (400 MHz, C_6D_6, 298 K).

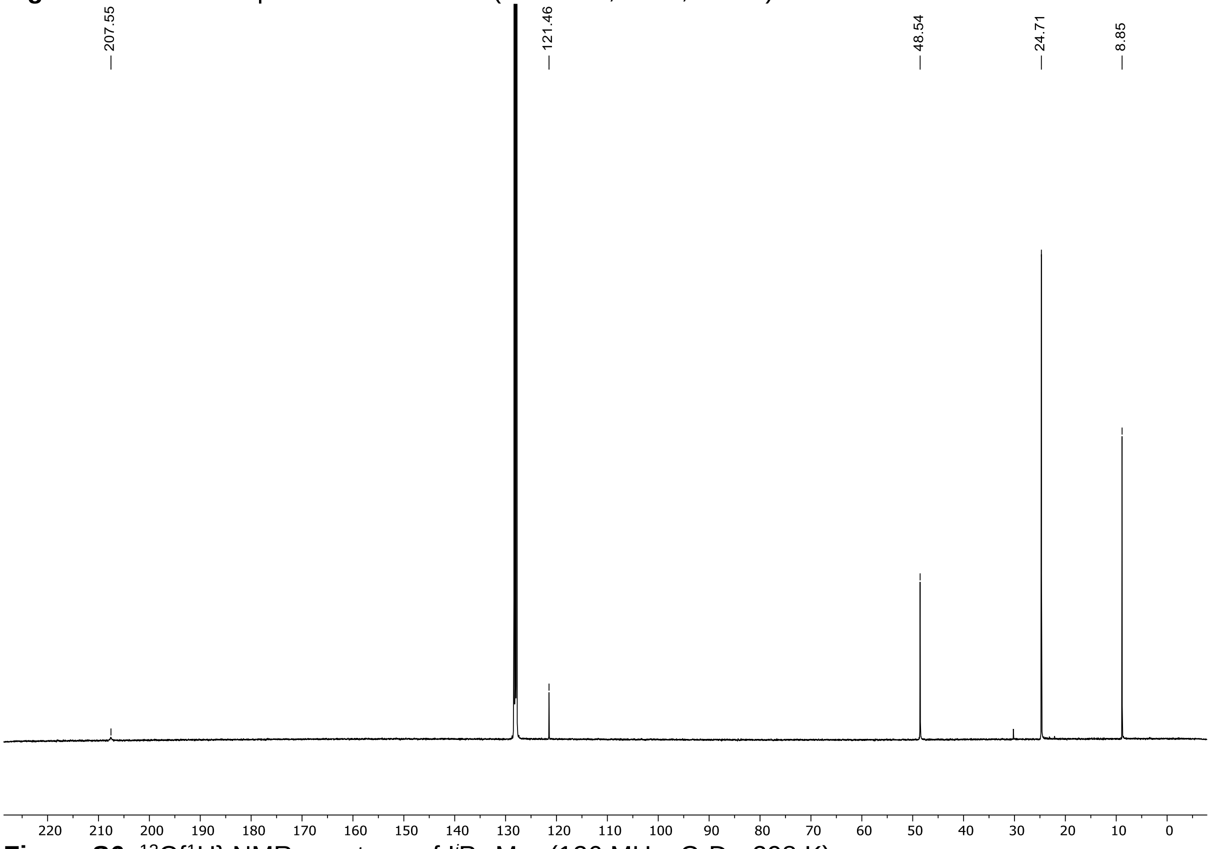

Figure S6. ^{13}C{^{1}H} NMR spectrum of I^{i}Pr$_2$Me$_2$ (126 MHz, C_6D_6, 298 K).

^{Tipp}TerSn{N(SiMe$_3$)$_2$} (1c): ^{1}H NMR (400 MHz, C_6D_6, 298 K): δ = 0.01 (s, 18H, Si(CH_3)$_3$), 1.13 (d, $^3J_{H,H}$ = 6.7 Hz, 12H, *o*-CH(C*H*$_3$)$_2$-C_6H_3), 1.26 (d, $^3J_{H,H}$ = 7.0 Hz, 12H, *p*-CH(C*H*$_3$)$_2$-C_6H_3), 1.45 (d, $^3J_{H,H}$ = 6.8 Hz, 12H, *o*-CH(C*H*$_3$)$_2$-C_6H_3), 2.83 (hept, $^3J_{H,H}$ = 6.9 Hz, 2H, *p*-C*H*(CH_3)$_2$-C_6H_3), 3.34 (hept, $^3J_{H,H}$ = 6.8 Hz, 4H, *o*-C*H*(CH_3)$_2$-C_6H_3), 7.23 (s, 4H, *m*-C*H*$_{Aryl}$-C_6H_3), 7.27-7.30 (m, 1H, *p*-CH_{Aryl}Sn), 7.34-7.36 (m, 2H, *m*-CH_{Aryl}Sn) ppm; **^{13}C{^{1}H} NMR** (126 MHz, C_6D_6, 298 K): δ = 6.2 (Si(CH_3)$_3$), 23.1 (*o*-CH($\underline{C}H_3$)$_2$-C_6H_3), 24.3 (*p*-CH($\underline{C}H_3$)$_2$-C_6H_3), 27.3 (*o*-CH($\underline{C}H_3$)$_2$-C_6H_3), 31.4 (*o*-$\underline{C}$H(CH_3)$_2$-C_6H_3), 34.8 (*p*-$\underline{C}$H(CH_3)$_2$-C_6H_3), 122.2 (*m*-$\underline{C}H_{Aryl}$-C_6H_3), 126.9 (*p*-CH_{Aryl}Sn), 131.7 (*m*-CH_{Aryl}Sn), 135.1 ($C_{q,ipso,Tipp}$), 144.9 (*o*-$C_{q,Aryl}$Sn), 147.5 (*o*-$\underline{C}_{q,Aryl}$-C_6H_3), 149.7 (*p*-$\underline{C}_{q,Aryl}$-C_6H_3), 185.5 ($C_{q,Aryl}$Sn) ppm; **^{119}Sn{^{1}H} NMR** (149 MHz, C_6D_6, 298 K): δ = 1198.9 ppm.

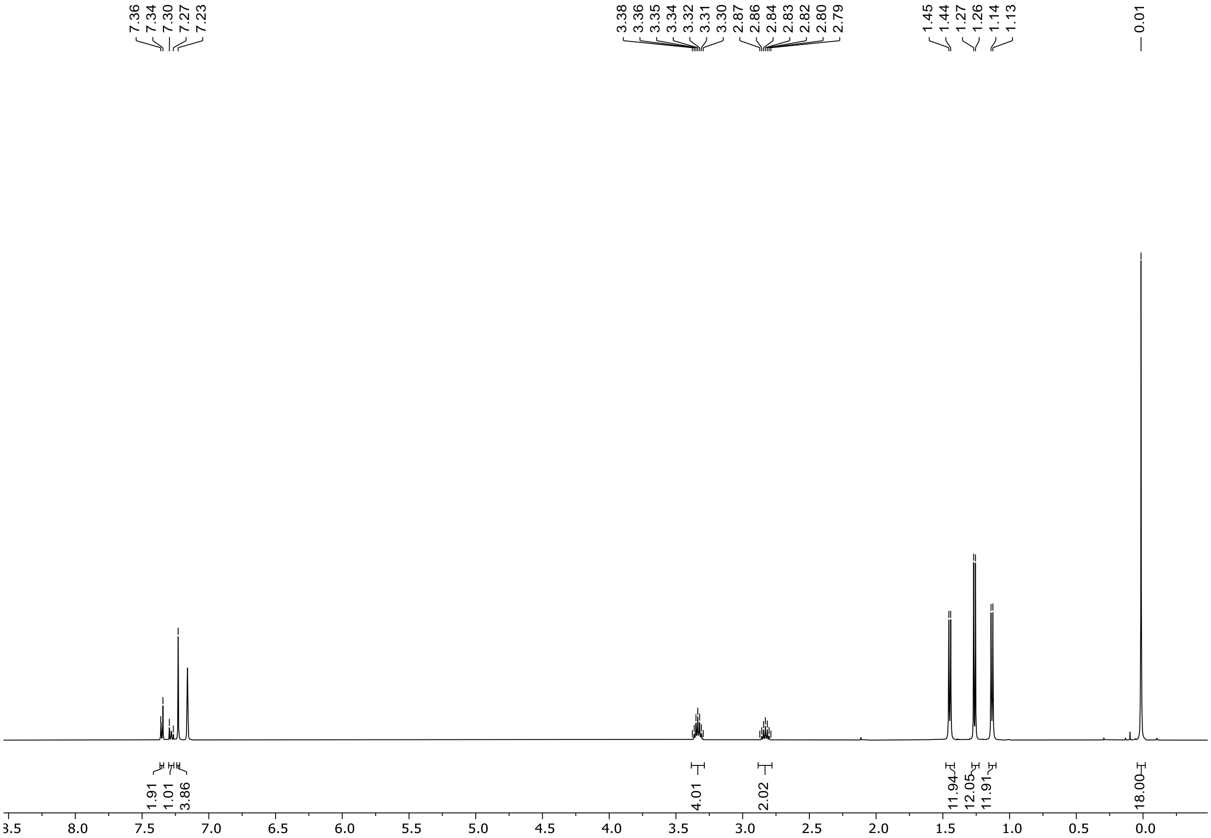

Figure S7. ^{1}H NMR spectrum of ^{Tipp}TerSn{N(SiMe$_3$)$_2$} (**1c**) (400 MHz, C_6D_6, 298 K).

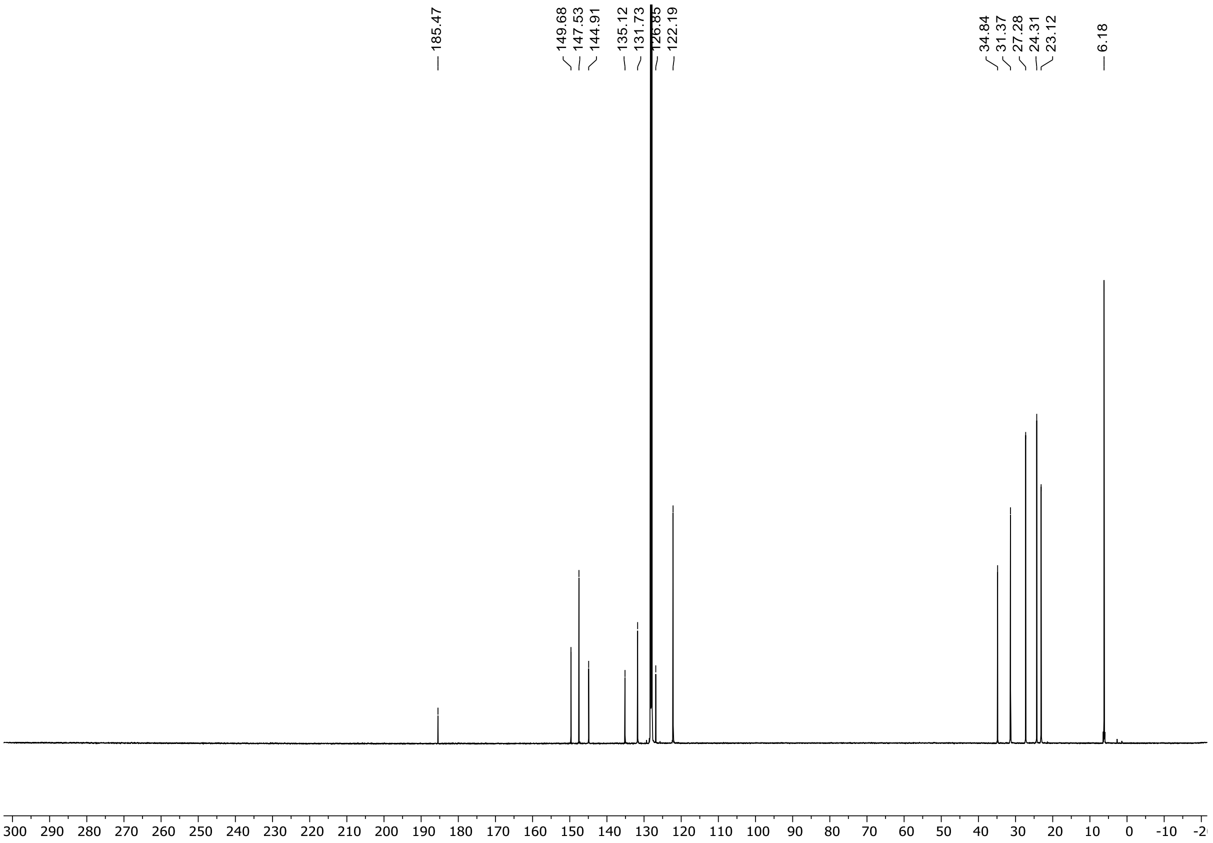

Figure S8. $^{13}C\{^1H\}$ NMR spectrum of ^{Tipp}TerSn{N(SiMe$_3$)$_2$} (**1c**) (126 MHz, C_6D_6, 298 K).

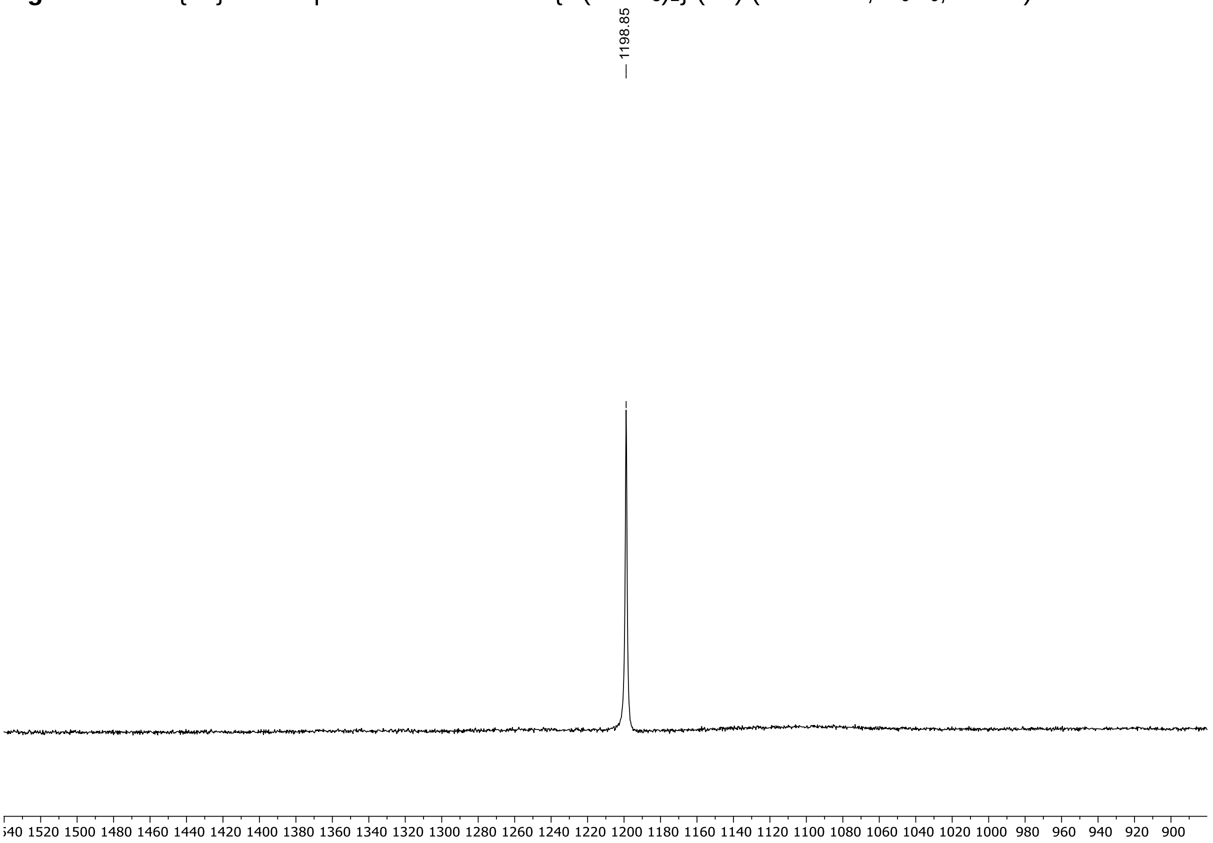

Figure S9. $^{119}Sn\{^1H\}$ NMR spectrum of ^{Tipp}TerSn{N(SiMe$_3$)$_2$} (**1c**) (149 MHz, C_6D_6, 298 K).

Reaction of $^{Mes}TerSn\{N(SiMe_3)_2\}$ (1a) with IMe_4 – Synthesis of $^{Mes}TerSn(IMe_4)\{N(SiMe_3)_2$ (2a) and of the C–H Activation Product 3a

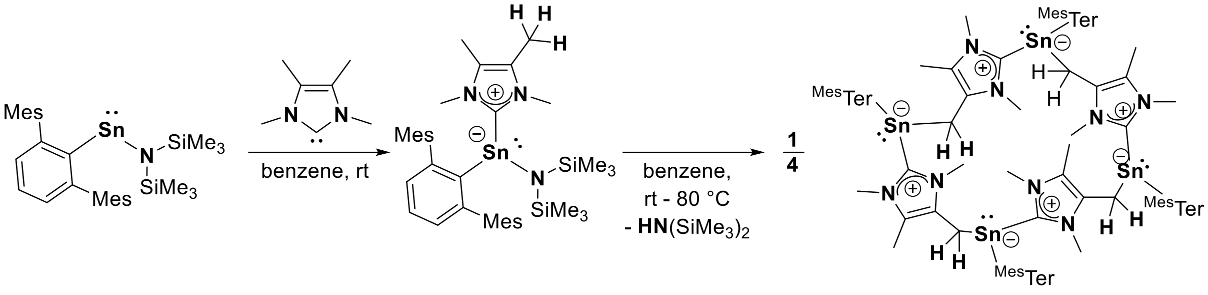

In a typical experiment, $^{Mes}TerSn\{N(Si(CH_3)_3)_2\}$ (**1a**) (0.030 g, 0.051 mmol) and IMe_4 (0.006 g, 0.051 mmol) were dissolved in 0.5 mL of C_6D_6 or 1 mL of benzene, resulting in the immediate formation of $^{Mes}TerSn(IMe_4)\{N(Si(CH_3)_3)_2\}$ (**2a**), as confirmed by ^1H NMR spectroscopy (Figure S10). Additionally, the formation of $HN(SiMe_3)_2$ can be observed as evident by its characteristic ^1H NMR chemical shift (δ^1H = 0.10 ppm). The amount of $HN(SiMe_3)_2$ increases over time and can be accelerated by heating the reaction mixture to 80 °C over a prolonged time. By slow evaporation of the benzene solution, the formation of colourless crystals can be observed which were identified as the C–H activation product **3**. These crystals were suitable for single crystal X-ray diffraction.

For the isolation of $^{Mes}TerSn(IMe_4)\{N(SiMe_3)_2\}$ (**2a**), $^{Mes}TerSn\{N(SiMe_3)_2\}$ (**1a**) (0.030 g, 0.051 mmol) and IMe_4 (0.006 g, 0.051 mmol) were dissolved in 1 mL of benzene, and the reaction mixture was stirred for 10 minutes at room temperature. All volatile components were removed under vacuum, the remaining solid was dissolved in 1 mL of Et_2O, filtered and stored at -30 °C to give **2a** as slightly yellow crystals. These crystals were suitable for single crystal X-ray diffraction. The resulting crystals of **2a** can be stored at -30 °C for at least a month without any signs of decomposition and at room temperature for at least one week. However, by dissolving **2a** in *e.g.* C_6D_6 and immediate measurement of the ^1H NMR spectroscopy the follow-up reaction to **3a** starts. For obtaining multinuclear NMR data, **2a** was generated *in situ* and immediately characterized by NMR spectroscopy.

For the isolation of **3a**, a crystalline fraction of **2a** (0.030 g, 0.042 mmol) was either dissolved in 0.5 mL of C_6D_6 or toluene-d_8 and heated to 80 °C until complete consumption of **2a**. Figure S15 shows that the reaction in toluene-d_8 is finished after approximately 3 h at that temperature. The volume of the benzene solution was reduced to incipient crystallization, the crystalline solid was collected by filtration, and all volatile components were removed under vacuum to yield **3a** as a colourless powder which is only marginally soluble in common organic solvents.

Data of $^{Mes}TerSn(IMe_4)\{N(SiMe_3)_2\}$ (2a):
Yield: 0.022 g (0.031 mmol; 61%).
^1H NMR (400 MHz, C_6D_6, 298 K): δ = 0.24 (s, 18H, $Si(CH_3)_3$), 1.26 (s, 6H, NC_qCH_3), 2.05 (s, 6H, CH_3), 2.27 (s, 6H, CH_3), 2.50 (s, 6H, CH_3), 3.23 (s, 6H, NCH_3), 6.52 (m, 2H, CH_{Aryl}), 6.73 (m, 2H, CH_{Aryl}), 7.05-7.06 (m, 2H, CH_{Aryl}), 7.26-7.29 (m, 1H, CH_{Aryl}) ppm.
^1H NMR (400 MHz, C_7D_8, 298 K): δ = 0.15 (s, 18H, $Si(CH_3)_3$), 1.31 (s, 6H, NC_qCH_3), 2.04 (s, 6H, CH_3), 2.22 (s, 6H, CH_3), 2.43 (s, 6H, CH_3), 3.23 (s, 6H, NCH_3), 6.47 (m, 2H, CH_{Aryl}), 6.67 (m, 2H, CH_{Aryl}), 6.97-6.99 (m, 2H, CH_{Aryl}), 7.20-7.25 (m, 1H, CH_{Aryl}) ppm.
$^{13}C\{^1H\}$ NMR (101 MHz, C_6D_6, 298 K): δ = 7.1 ($Si(CH_3)_3$), 8.1 ($NC_q\underline{C}H_3$), 21.0 (CH_3), 22.8 (CH_3), 23.2 (CH_3), 35.3 (NCH_3), 124.0 ($N\underline{C}_qCH_3$), 126.9 (CH_{Aryl}), 127.8 (CH_{Aryl})*, 1281. (CH_{Aryl})*, 128.9 (CH_{Aryl}), 135.6 ($C_{q,Aryl}$), 135.7 ($C_{q,Aryl}$), 137.0 ($C_{q,Aryl}$), 143.2 ($C_{q,Aryl}$), 149.3 ($C_{q,Aryl}$), 169.6 ($C_{q,Aryl}Sn$), 179.8 (C_{NHC}) ppm.
* = overlap with C_6D_6 signal and assigned by $^1H/^{13}C$-HSQC
$^{119}Sn\{^1H\}$ NMR (149 MHz, C_6D_6, 298 K): δ = -15.7 ppm.
MS: No meaningful results could be obtained using LIFDI, ESI and CI MS.

EA: Anal. calcd. for $C_{37}H_{55}N_3Si_2Sn$: C, 62.00; H, 7.73; N, 5.86; Found: C, 61.43; H, 7.64; N, 5.71.

Data of the C–H activation product 3a:
Yield: 0.017 g (0.008 mmol; 73%). **Note**: Due to the low solubility in common organic solvents preventing sufficient NMR analysis, and systematically low carbon values by elemental combustion analysis, the yield has to be taken with caution.
MS: No meaningful results could be obtained using LIFDI, ESI and CI MS.
EA: Anal. calcd. for $C_{124}H_{144}N_8Sn_4$: C, 67.05; H, 6.53; N, 5.04; Found: C, 65.48; H, 6.50; N, 4.95.

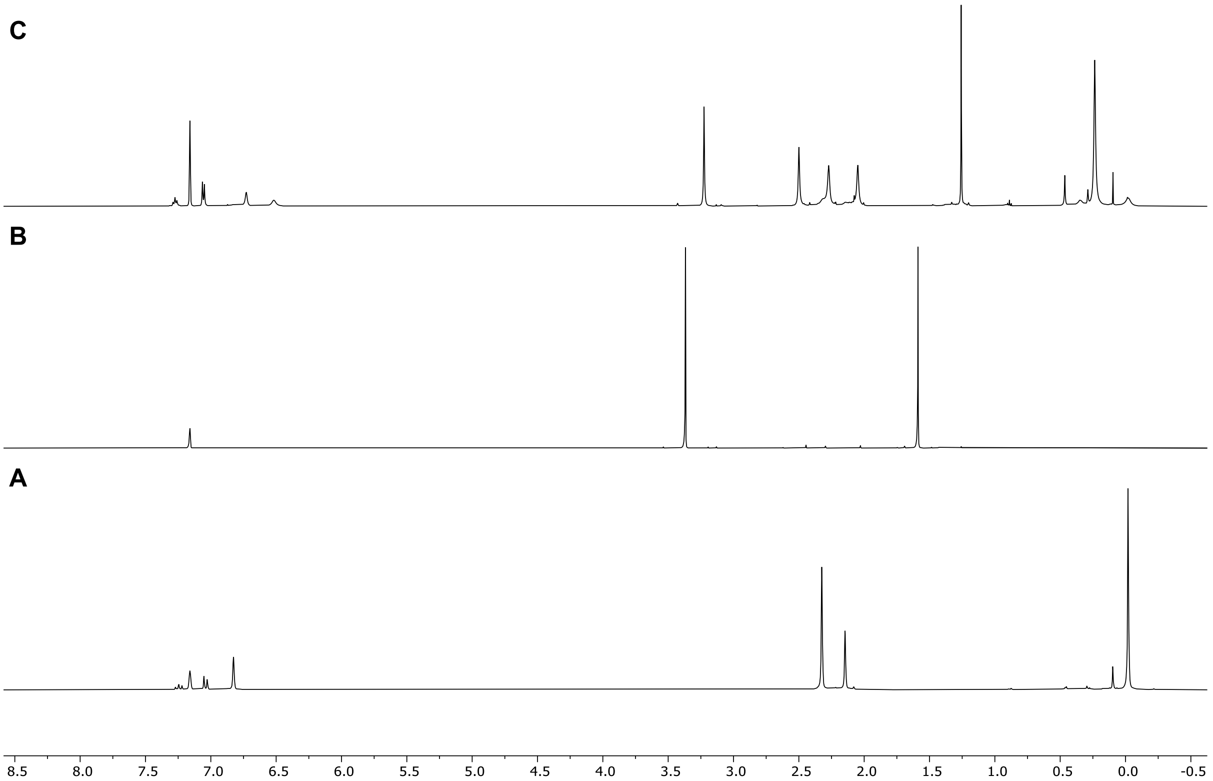

Figure S10. Stacked ^{1}H NMR spectra of ^{Mes}TerSn{N(SiMe$_3$)$_2$} (**1a**) (**A**), IMe$_4$ (**B**), and of ^{Mes}TerSn(IMe$_4$){N(SiMe$_3$)$_2$} (**2a**) (**C**) measured after 5 minutes at 298 K.

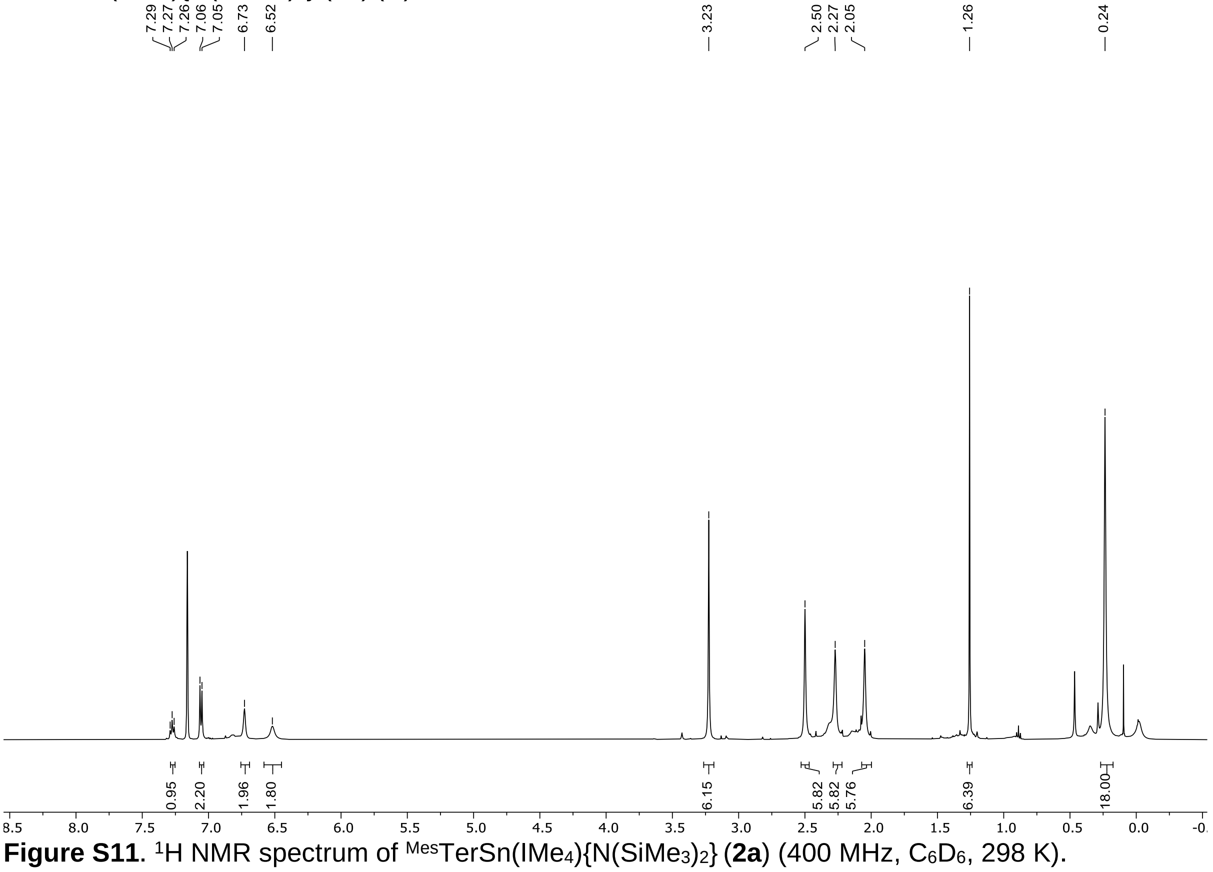

Figure S11. ^{1}H NMR spectrum of ^{Mes}TerSn(IMe$_4$){N(SiMe$_3$)$_2$} (**2a**) (400 MHz, C_6D_6, 298 K).

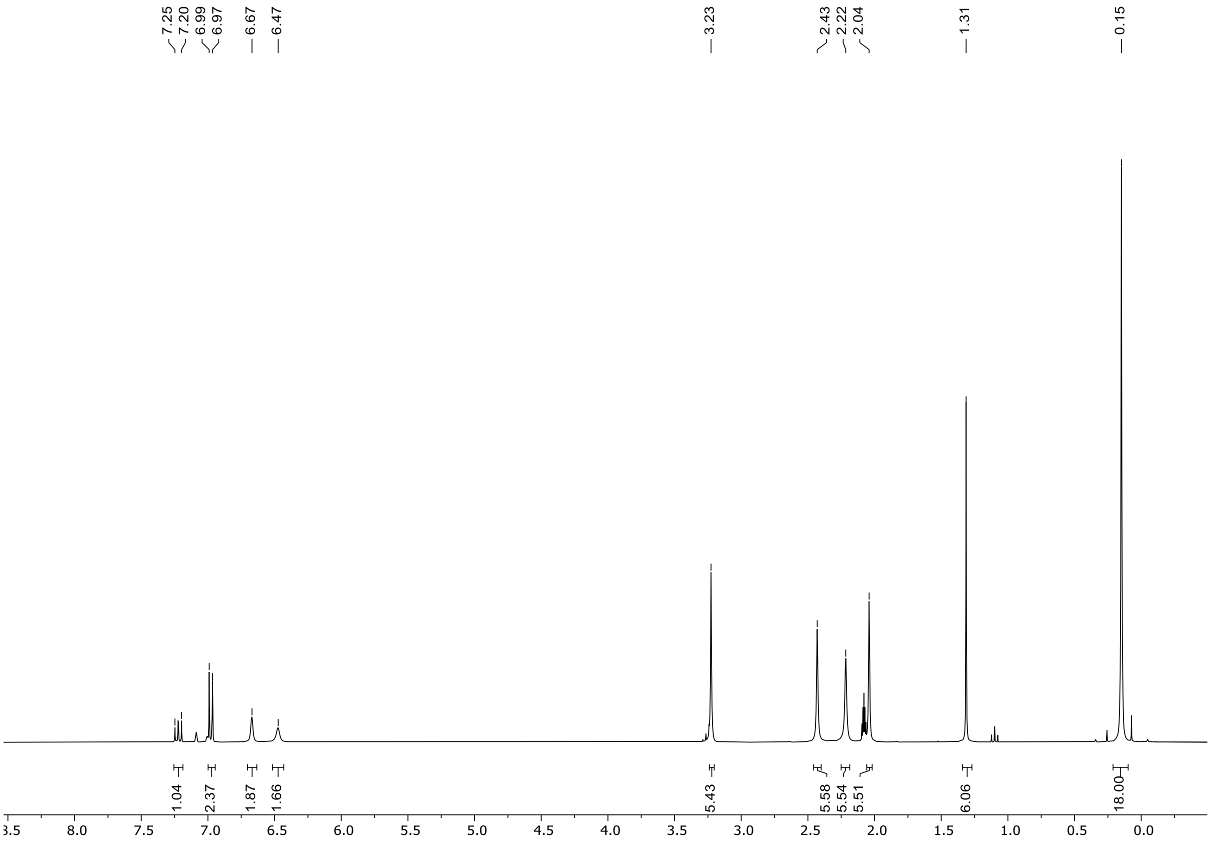

Figure S12. ^{1}H NMR spectrum of $^{Mes}TerSn(IMe_4)\{N(SiMe_3)_2\}$ (**2a**) (400 MHz, C_7D_8, 298 K).

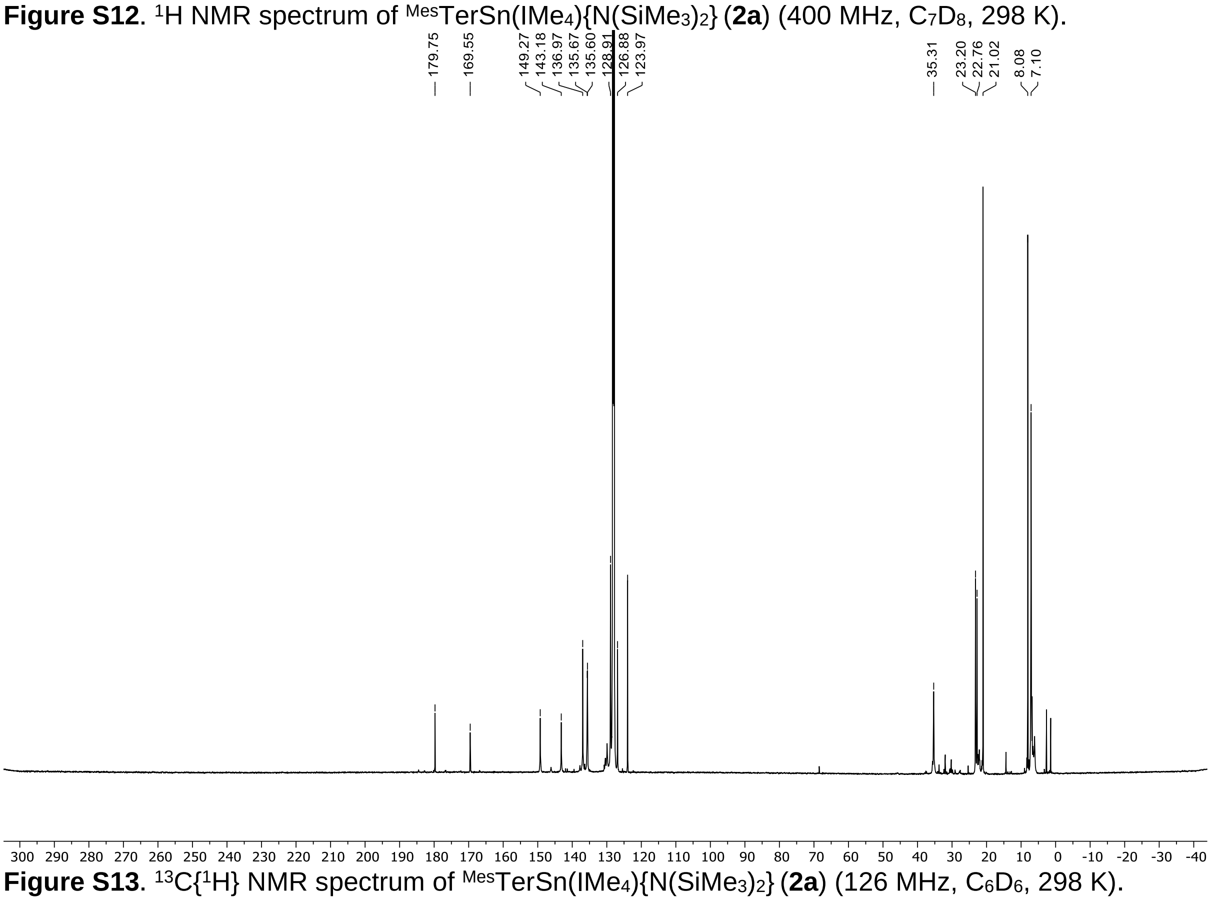

Figure S13. $^{13}C\{^{1}H\}$ NMR spectrum of $^{Mes}TerSn(IMe_4)\{N(SiMe_3)_2\}$ (**2a**) (126 MHz, C_6D_6, 298 K).

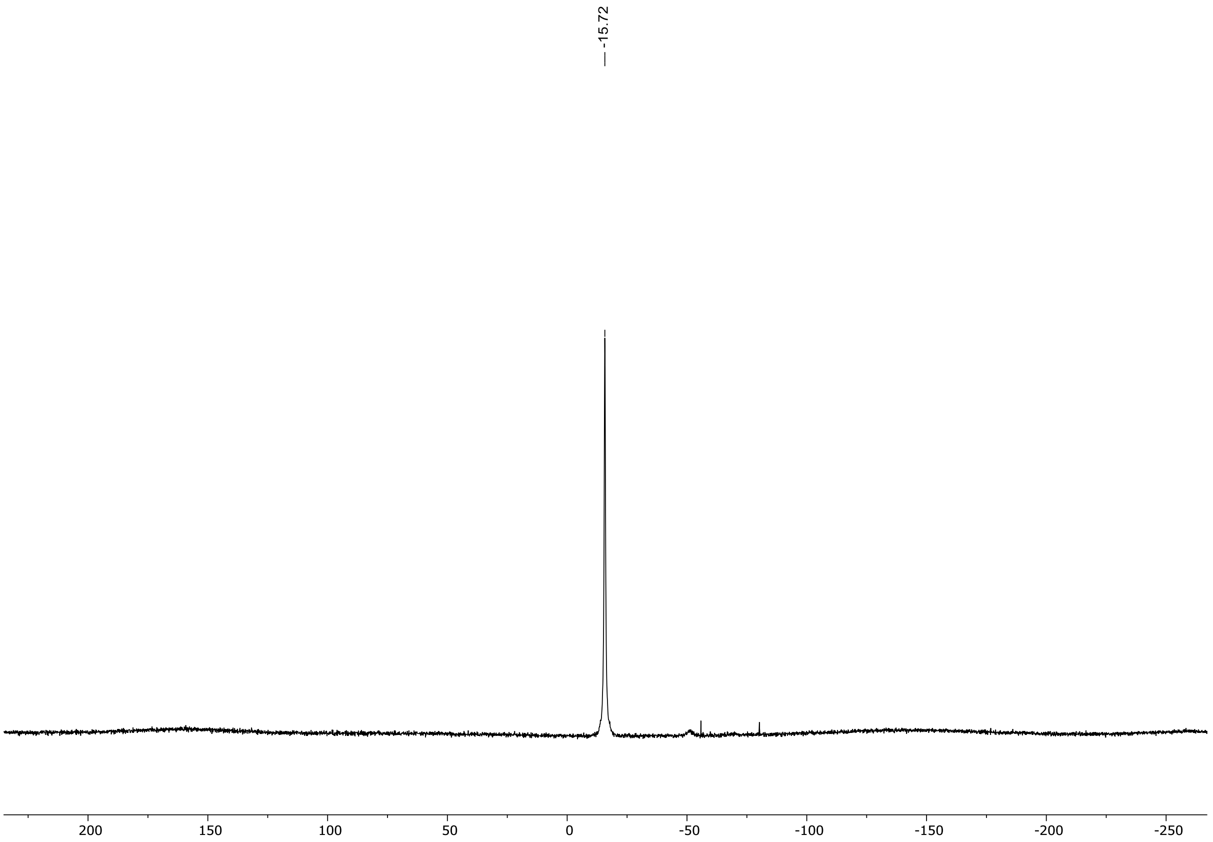

Figure S14. $^{119}Sn\{^{1}H\}$ NMR spectrum of $^{Mes}TerSn(IMe_4)\{N(SiMe_3)_2\}$ (**2a**) (149 MHz, C_6D_6, 298 K).

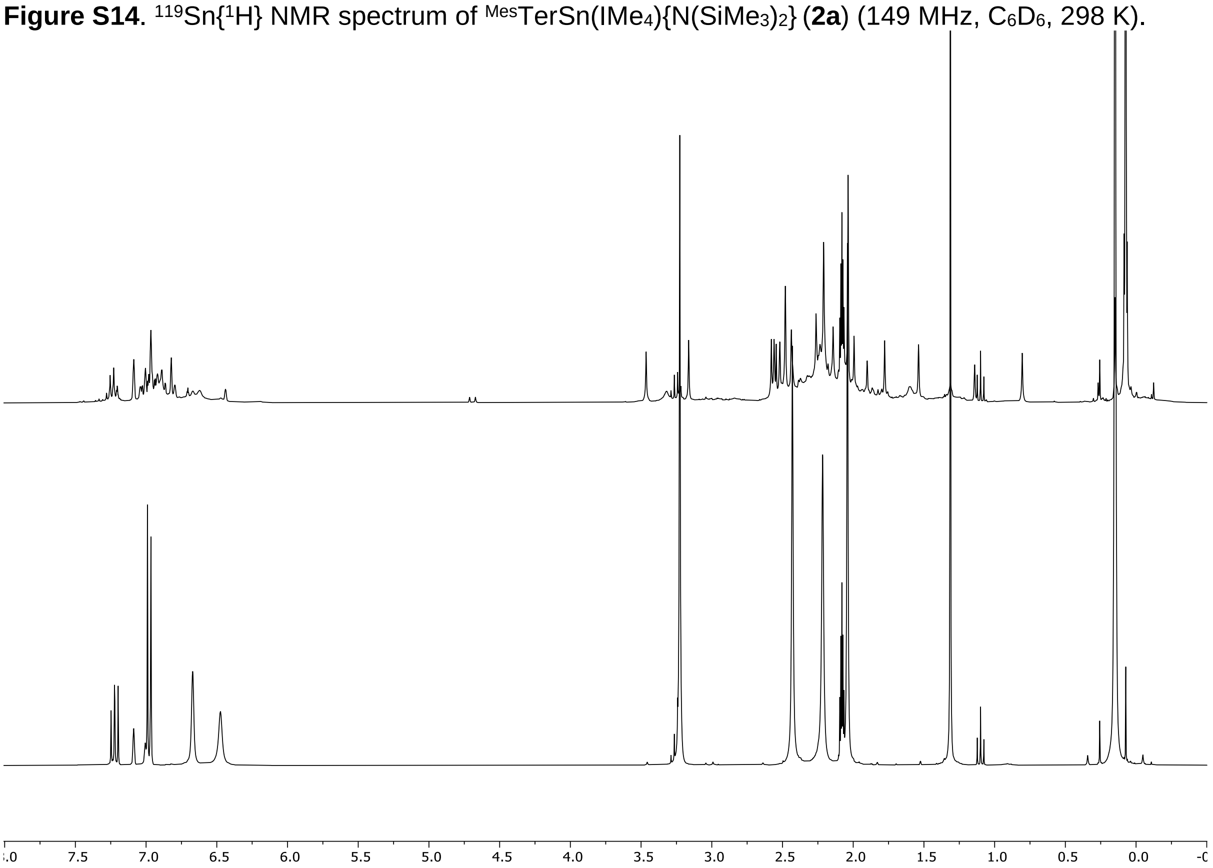

Figure S15. Bottom: ^{1}H NMR spectrum of $^{Mes}TerSn(IMe_4)\{N(SiMe_3)_2\}$ (**2a**) measured after 5 minutes at room temperature; Top: Corresponding ^{1}H NMR spectrum obtained after 3 hours at 80 °C (400 MHz, C_7D_8, 298 K); 0.12 ppm: $HN(SiMe_3)_2$.

Reaction Monitoring and Isolation of 3a

Following the procedure described above, ^{Mes}TerSn{N(Si(CH$_3$)$_3$)$_2$} (**1a**) (0.030 g, 0.051 mmol) was dissolved in 0.5 mL of C_6D_6. Figures S16 and S17 (bottom) display the respective ^{1}H NMR spectrum of **1a**, along with an excerpt of the high-field region. In a glove box, IMe$_4$ (0.006 g, 0.051 mmol) was added to the solution, and the reaction mixture was subsequently analysed by ^{1}H NMR spectroscopy. Figure S18 (top left) shows the solution in a J. Young NMR tube immediately after the addition of IMe$_4$, highlighting the colour change from orange (characteristic of **1a**) to yellow (indicative of the formation of ^{Mes}TerSn(IMe$_4$){N(Si(CH$_3$)$_3$)$_2$} (**2a**)). The corresponding ^{1}H NMR spectrum and a high-field region excerpt of this solution are presented in Figures S16 and S17 (second from bottom), consistent with the formation of **2a**. After two hours at room temperature, Figures S16 and S17 (middle) show the corresponding ^{1}H NMR spectrum, revealing an increased formation of HN(Si(CH$_3$)$_3$)$_2$ (δ^1H = 0.10 ppm). The reaction mixture was then left undisturbed over the weekend, and Figures S16 and S17 (second from top) show the ^{1}H NMR spectra obtained at this stage, alongside the observation of a colourless crystalline precipitate. Upon heating the reaction mixture overnight, **2a** was fully consumed, and the formation of HN(Si(CH$_3$)$_3$)$_2$ was complete. Figures S16 and S17 (top) display the corresponding ^{1}H NMR spectrum of this final stage. Additionally, Figure S18 shows the resulting suspension (top right), captures the colourless crystalline precipitate (**3a**) (bottom left), and presents the crystalline precipitate under the microscope (bottom right).

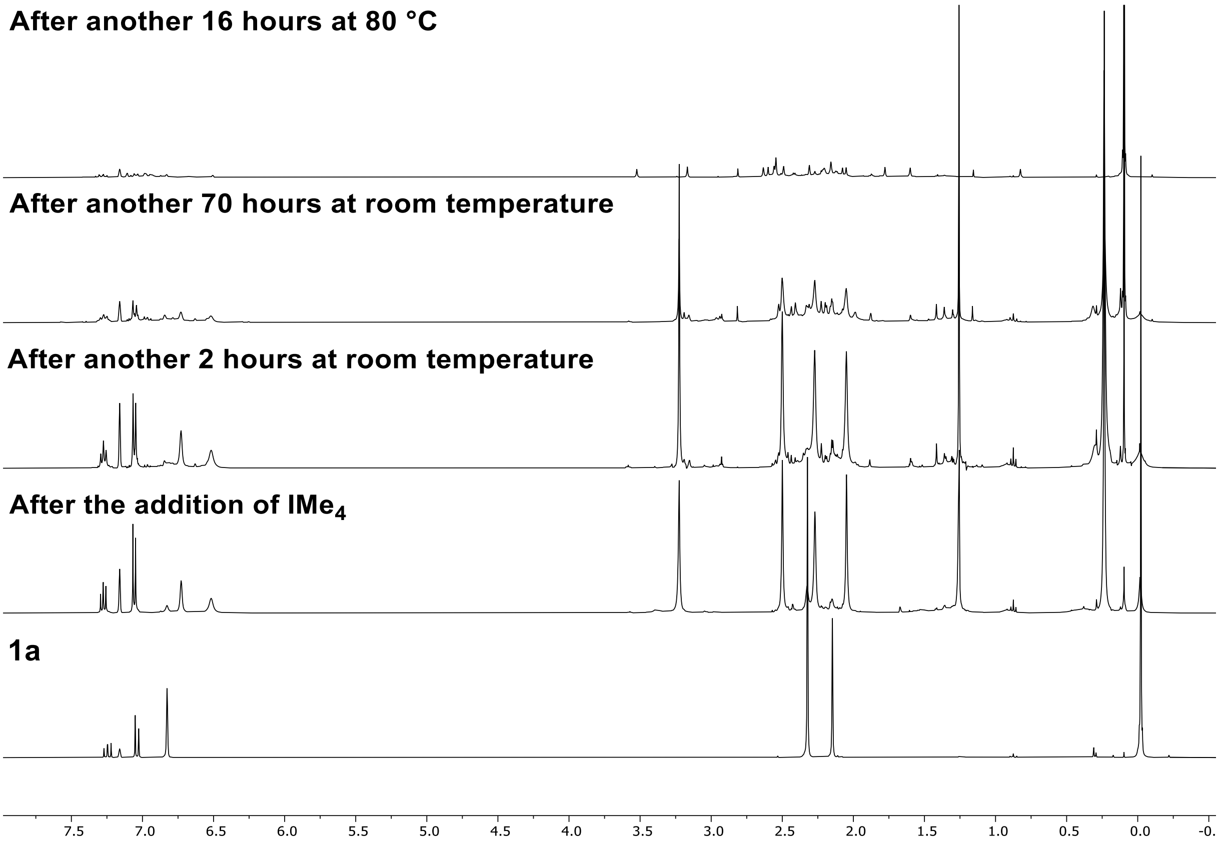

Figure S16. Monitoring of the reaction of ^{Mes}TerSn{N(Si(CH$_3$)$_3$)$_2$} (**1a**) with IMe$_4$ to stepwise yield ^{Mes}TerSn(IMe$_4$){N(Si(CH$_3$)$_3$)$_2$} (**2a**) and **3a** (400 MHz, C_6D_6, 298 K).

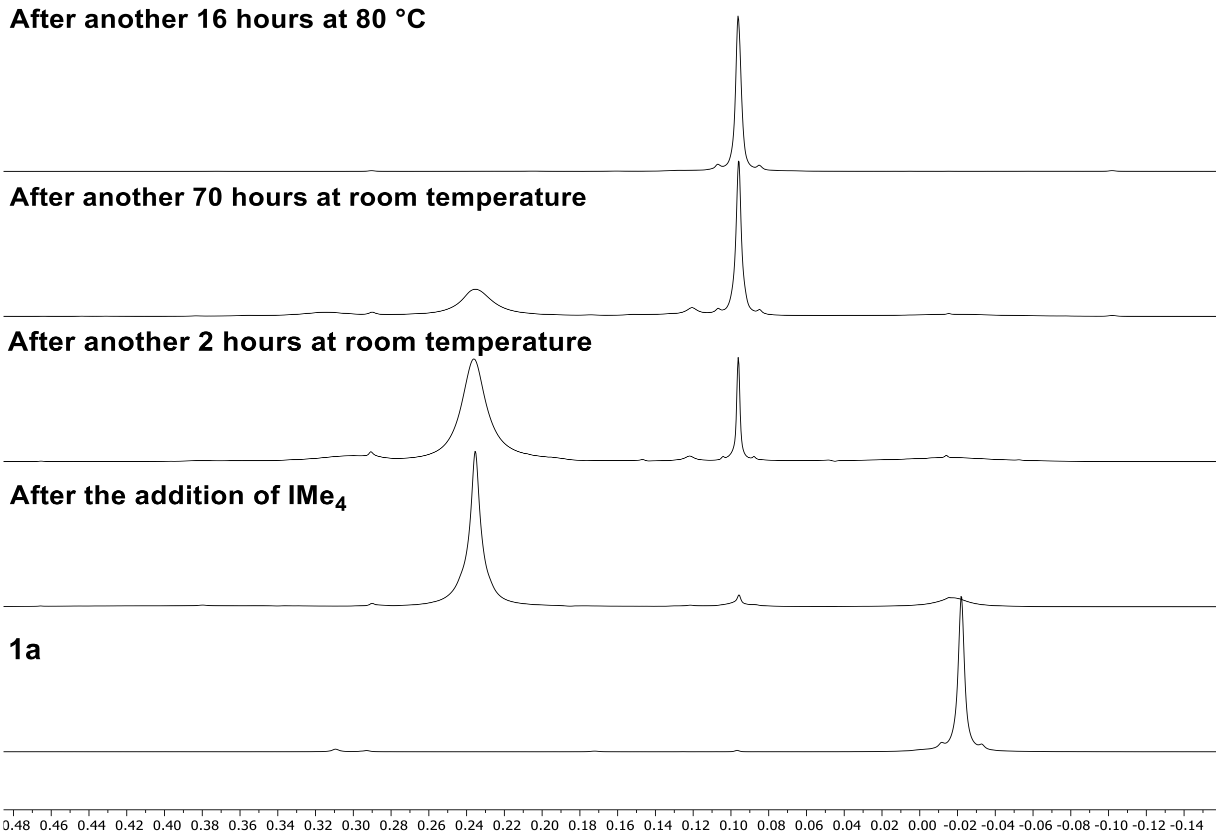

Figure S17. Excerpt of the high field region of Figure S16 (400 MHz, C_6D_6, 298 K).

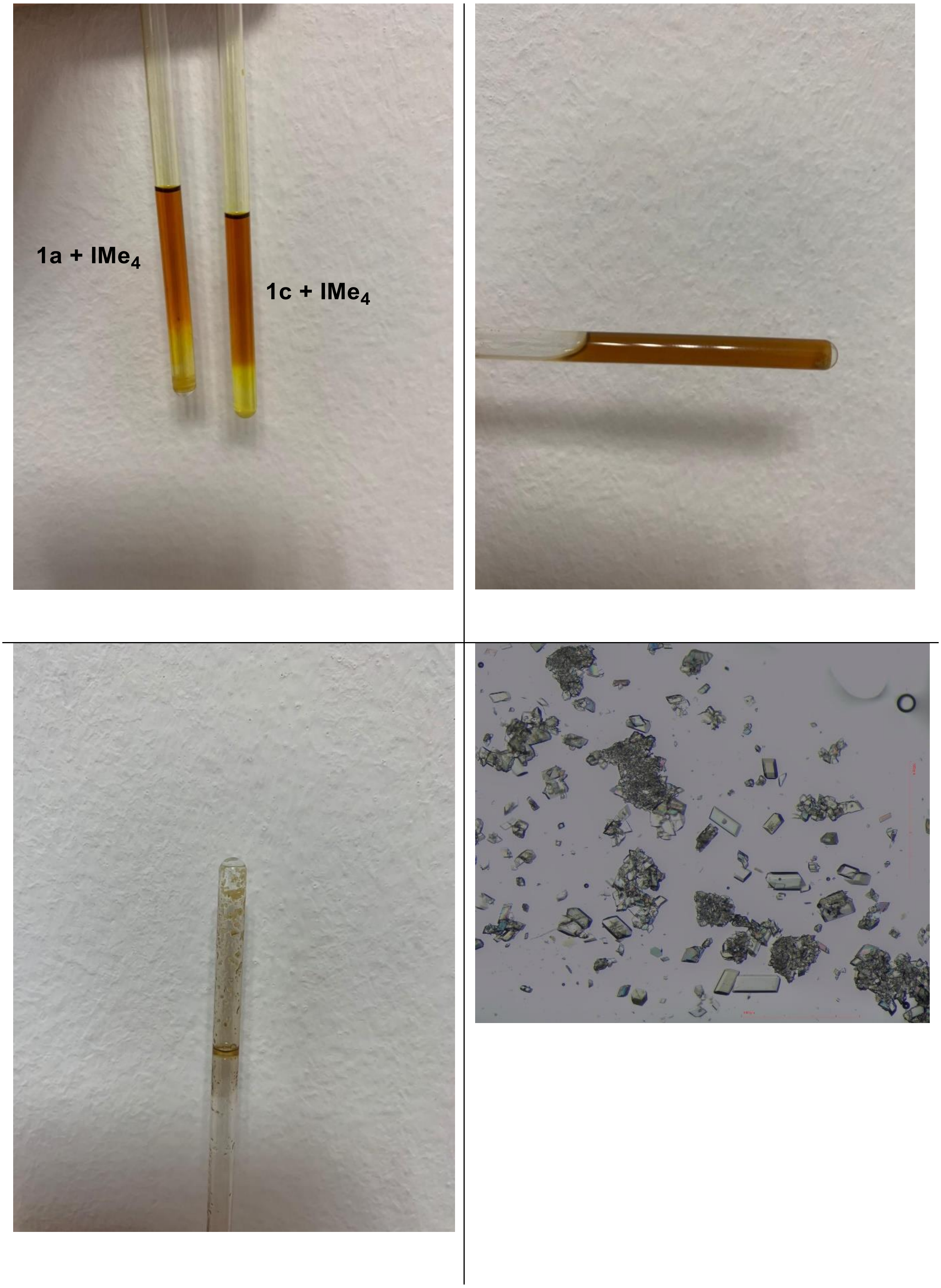

Figure S18. Photographs taken during the monitoring of the reaction between $^{Mes}TerSn\{N(Si(CH_3)_3)_2\}$ (**1a**) and IMe_4.

Reaction of $^{Dipp}TerSn\{N(SiMe_3)_2\}$ (1b) with IMe_4 – Synthesis of $^{Dipp}TerSn(IMe_4)\{N(SiMe_3)_2\}$ (2b) and of the C–H Activation Product 4b

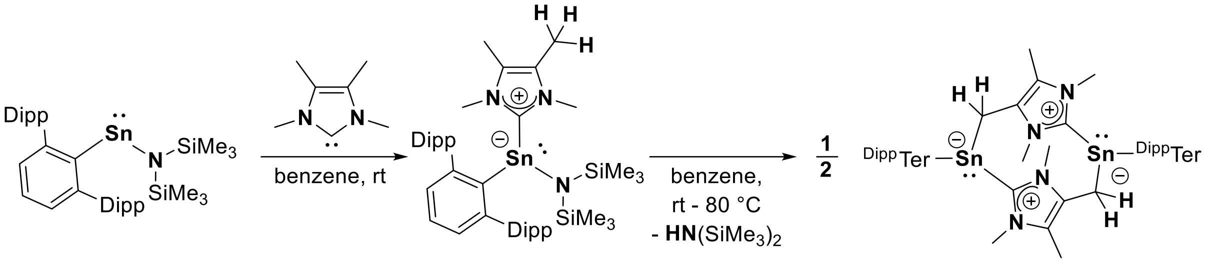

In a typical experiment, $^{Dipp}TerSn\{N(Si(CH_3)_3)_2$ (**1b**) (0.030 g, 0.044 mmol) and IMe_4 (0.006 g, 0.044 mmol) were dissolved in 0.5 mL of C_6D_6, 1 mL of benzene or 0.5 mL of toluene-d_8, resulting in the immediate formation of $^{Dipp}TerSn(IMe_4)\{N(Si(CH_3)_3)_2\}$ (**2b**), as confirmed by ^1H NMR spectroscopy. Over time, the amount of $HN(SiMe_3)_2$ increases and can be accelerated by heating the reaction mixture to 80 °C over a prolonged time. By slow evaporation of a benzene solution, the formation of colourless crystals can be observed which were identified as the C–H activation product **4b**. These crystals were suitable for single crystal X-ray diffraction. In other attempts, the crystals were collected by filtration and dried under vacuum to give **4b** as a colourless powder.

For the isolation of $^{Dipp}TerSn(IMe_4)\{N(SiMe_3)_2\}$ (**2b**), $^{Dipp}TerSn\{N(SiMe_3)_2\}$ (**1b**) (0.030 g, 0.044 mmol) and IMe_4 (0.006 g, 0.044 mmol) were dissolved in 1 mL of benzene, and the reaction mixture was stirred for 10 minutes at room temperature. All volatile components were removed under vacuum to obtain **2b** as an orange appearing solid. For obtaining multinuclear NMR data, **2b** was generated *in situ* and immediately characterized by multinuclear NMR spectroscopy in toluene-d_8.

Data of $^{Dipp}TerSn(IMe_4)\{N(SiMe_3)_2\}$ (2b):
Yield: 0.029 g (0.036 mmol; 82%).
^1H NMR (400 MHz, C_7D_8, 298 K): δ = 0.07 (s, 18H, $Si(CH_3)_3$), 1.06 (m(br), 12H, $CH(CH_3)_2$), 1.25 (m(br), 12H, $CH(CH_3)_2$), 1.37 (s, 6H, NC_qCH_3), 3.03 (s, 6H, NCH_3), 3.28 (hept, $^3J_{H,H}$ = 6.7 Hz, 4H, $CH(CH_3)_2$), 7.02-7.11 (m(br), 3H, CH_{Aryl})*, 7.13-7.21 (m, 6H, CH_{Aryl}) ppm.
* = overlap with C_7D_7H signals
$^{13}C\{^1H\}$ NMR (101 MHz, C_7D_8, 298 K): δ = 6.7 ($Si(CH_3)_3$), 8.1 ($NC_q\underline{C}H_3$), 23.6 ($CH(\underline{C}H_3)_2$), 26.2 ($CH(\underline{C}H_3)_2$), 30.9 ($\underline{C}H(CH_3)_2$), 35.0 (NCH_3), 123.1 (CH_{Aryl}), 124.6 ($N\underline{C}_qCH_3$)**, 132.0 (CH_{Aryl}), 147.3 ($C_{q,Aryl}$), 180.0 (C_{NHC}) ppm. Note: Due to significant signal broadening and overlap with the C_7D_8 signals, only the clearly assignable signals are listed.
** = overlap with C_7D_8 signal
$^{119}Sn\{^1H\}$ NMR (149 MHz, C_7D_8, 298 K): δ = -27.4 ppm.
MS: No meaningful results could be obtained using LIFDI, ESI and CI MS.
EA: Anal. calcd. for $C_{43}H_{67}N_3Si_2Sn$: C, 64.49; H, 8.43; N, 5.25; Found: C, 65.26; H, 8.26; N, 4.86.

Data of the C–H activation product 4b:
Yield: 0.014 g (0.011 mmol; 50%). **Note**: Due to the low solubility in common organic solvents preventing sufficient NMR analysis, and systematically low carbon values by elemental combustion analysis, the yield has to be taken with caution.
MS: No meaningful results could be obtained using LIFDI, ESI and CI MS.
EA: Anal. calcd. for $C_{74}H_{96}N_4Sn_2$: C, 69.49; H, 7.57; N, 4.38; Found: C, 67.28; H, 7.51; N, 4.12.

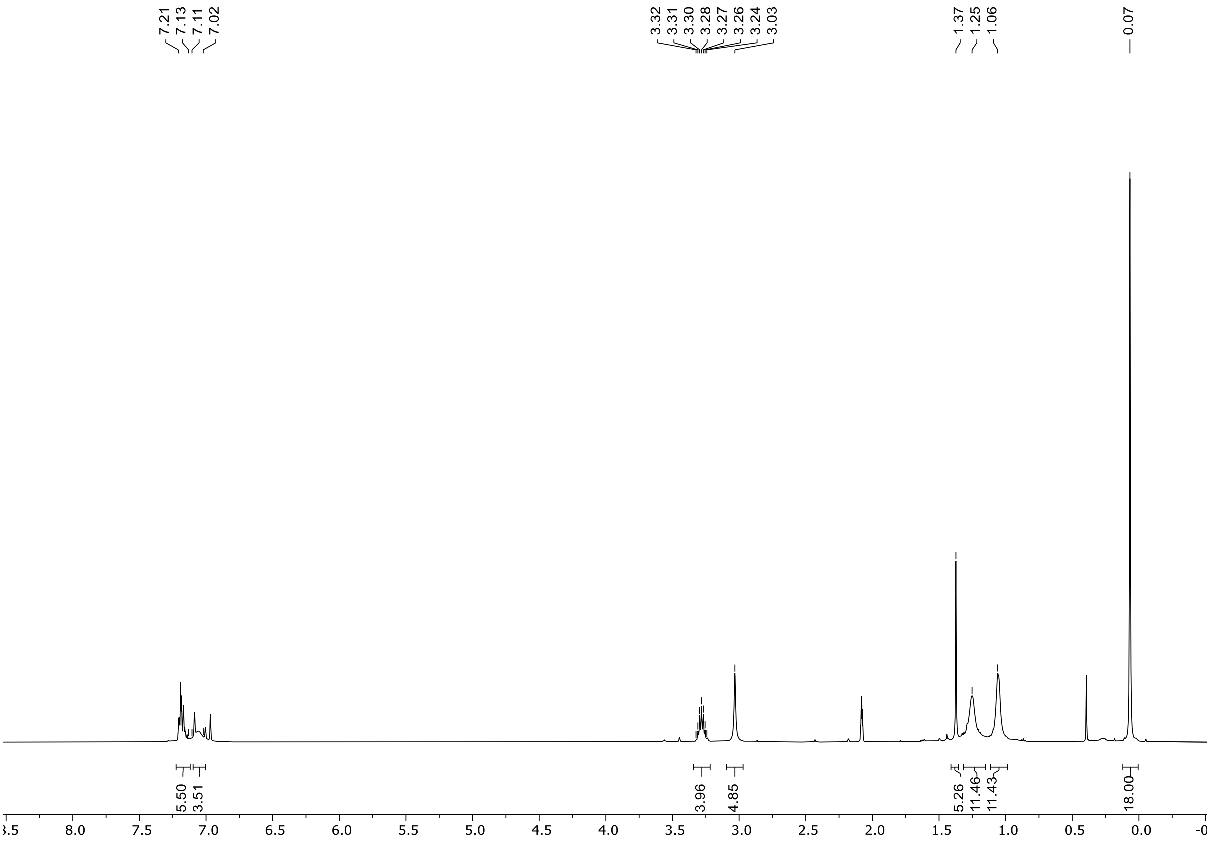

Figure S19. ^{1}H NMR spectrum of $^{Dipp}TerSn(IMe_4)\{N(SiMe_3)_2\}$ (**2b**) (400 MHz, C_7D_8, 298 K).

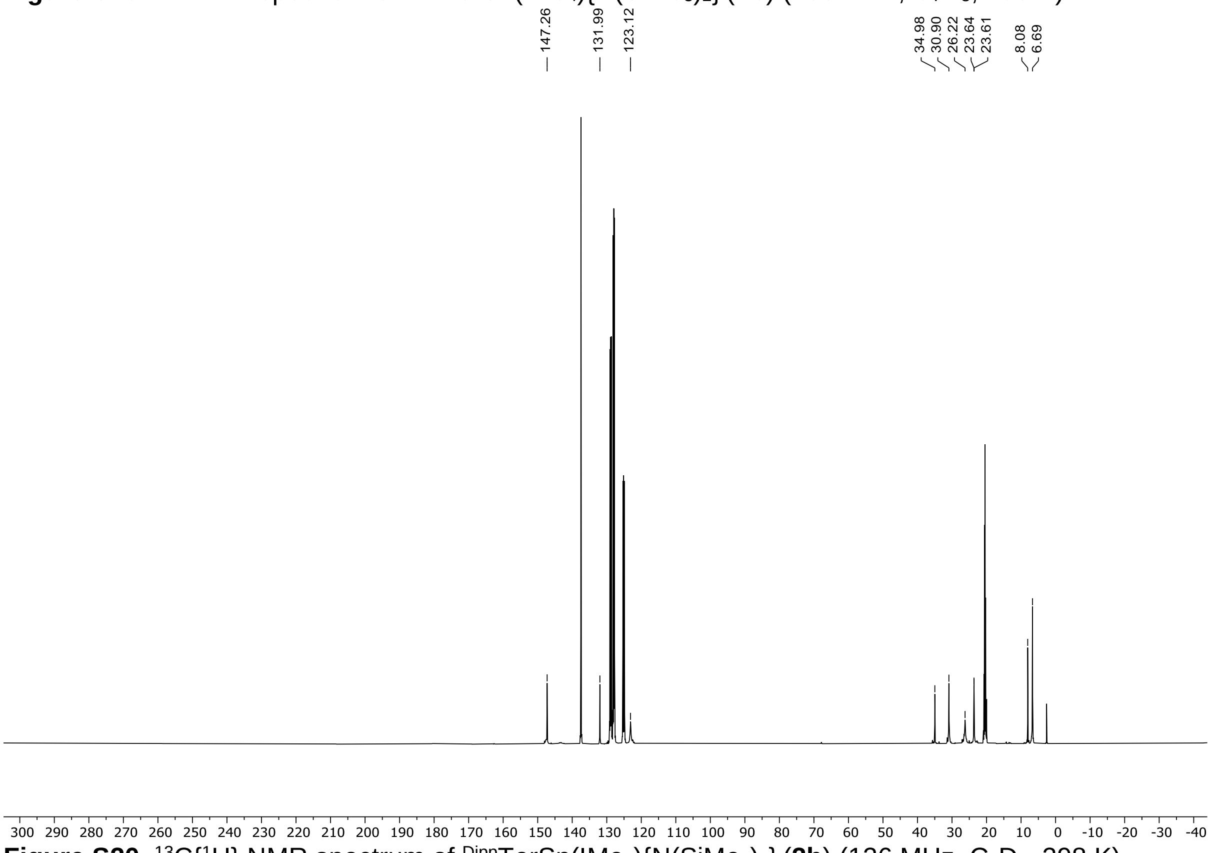

Figure S20. $^{13}C\{^{1}H\}$ NMR spectrum of $^{Dipp}TerSn(IMe_4)\{N(SiMe_3)_2\}$ (**2b**) (126 MHz, C_6D_6, 298 K).

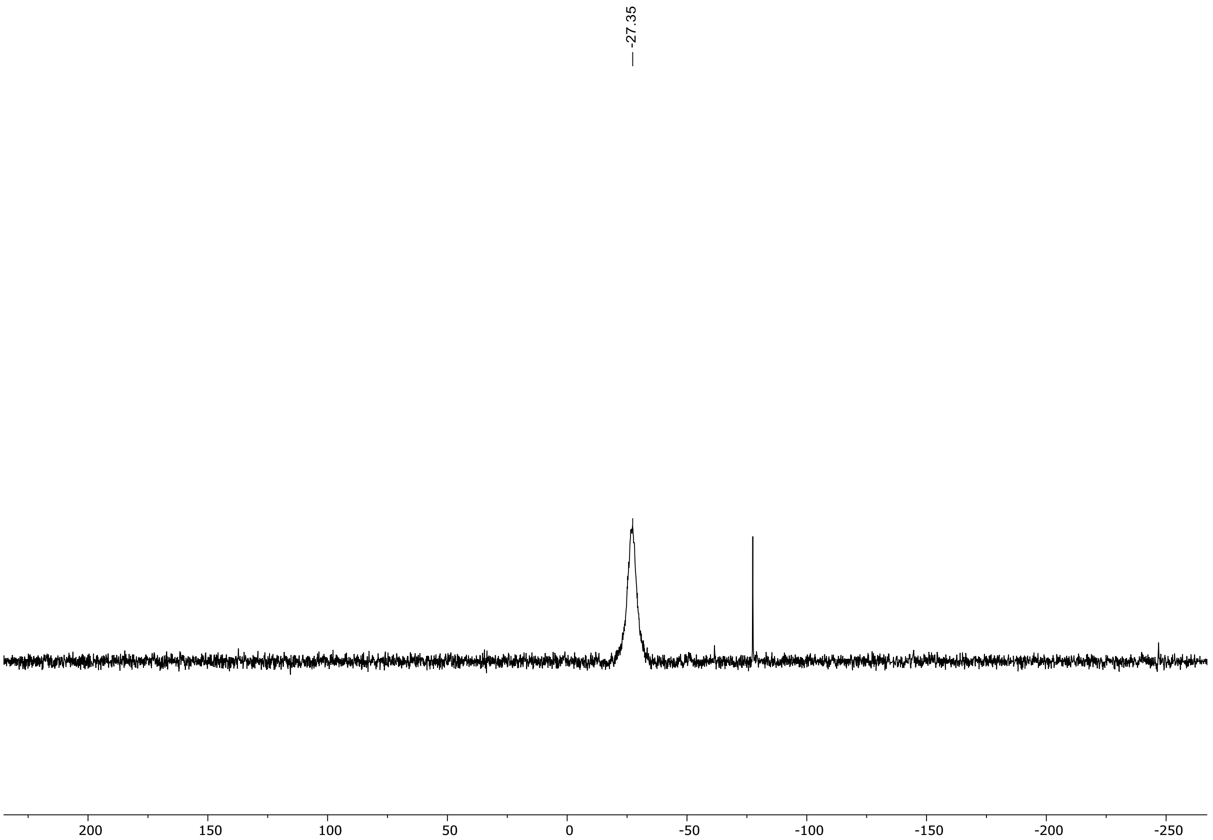

Figure S21. $^{119}Sn\{^{1}H\}$ NMR spectrum of $^{Dipp}TerSn(IMe_4)\{N(SiMe_3)_2\}$ (**2b**) (149 MHz, C_6D_6, 298 K); -77.5 ppm: unknown impurity.

Reaction Monitoring and Isolation of 4b

Following the procedure described above, $^{Dipp}TerSn\{N(Si(CH_3)_3)_2\}$ (**1b**) (0.030 g, 0.044 mmol) was dissolved in 0.5 mL of C_6D_6. Figures S22 and S23 (bottom) display the respective ^{1}H NMR spectrum of **1b**, along with an excerpt of the high-field region. In a glove box, IMe_4 (0.006 g, 0.044 mmol) was added to the solution, and the reaction mixture was subsequently analysed by ^{1}H NMR spectroscopy. The corresponding ^{1}H NMR spectrum and a high-field region excerpt of this solution are presented in Figures S22 and S23 (second from bottom), consistent with the formation of **2b**. The reaction mixture was then left undisturbed over the weekend, and Figures S22 and S23 (second from top) show the ^{1}H NMR spectra obtained at this stage, alongside the observation of a colourless crystalline precipitate. Upon heating the reaction mixture overnight, **2b** was fully consumed, and the formation of $HN(Si(CH_3)_3)_2$ ($\delta^{1}H$ = 0.10 ppm) was complete. Figures S22 and S23 (top) display the corresponding ^{1}H NMR spectrum of this final stage. The solution was transferred to a glove box and slightly concentrated under vacuum, leading to the further precipitation of **4b** as a colourless crystalline solid.

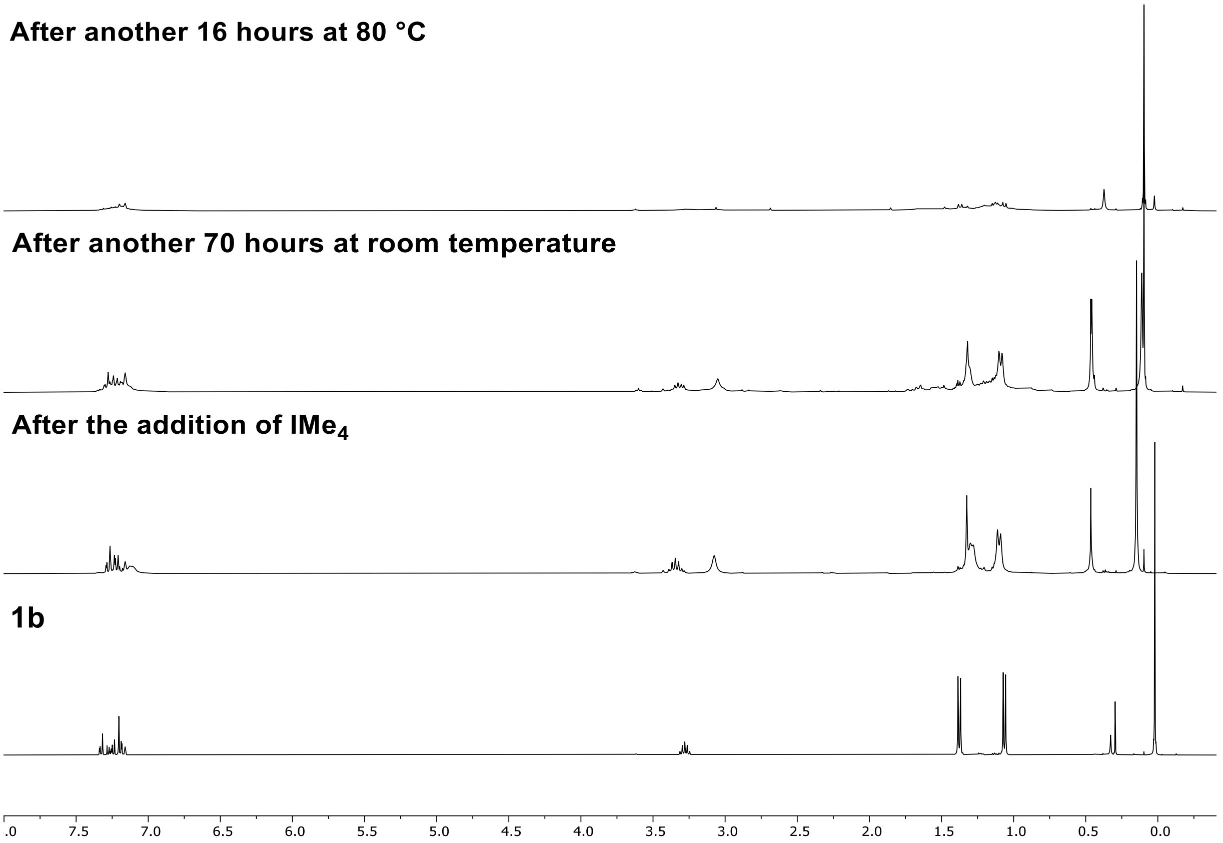

Figure S22. Monitoring of the reaction of ^{Dipp}TerSn{N(Si(CH$_3$)$_3$)$_2$} (**1b**) with IMe$_4$ to stepwise yield ^{Mes}TerSn(IMe$_4$){N(Si(CH$_3$)$_3$)$_2$} (**2b**) and **4b** (400 MHz, C$_6$D$_6$, 298 K).

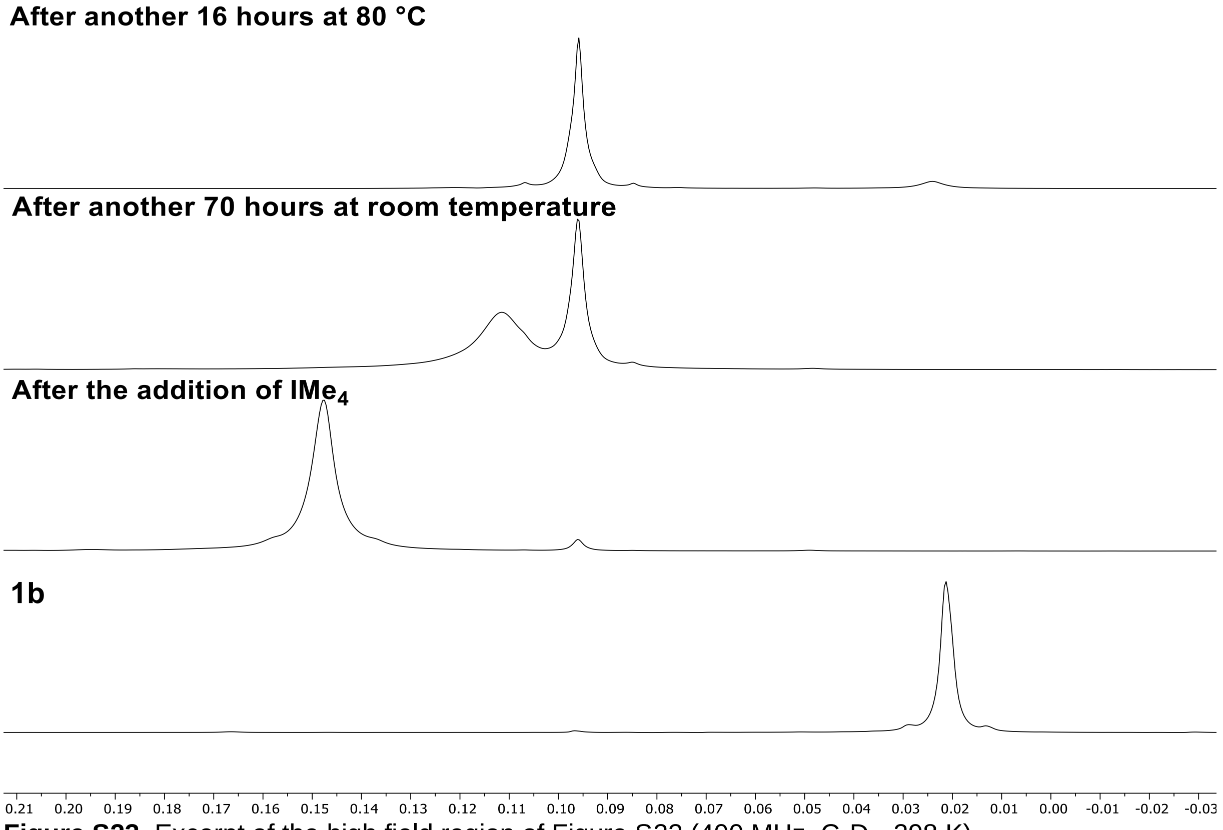

Figure S23. Excerpt of the high field region of Figure S22 (400 MHz, C$_6$D$_6$, 298 K).

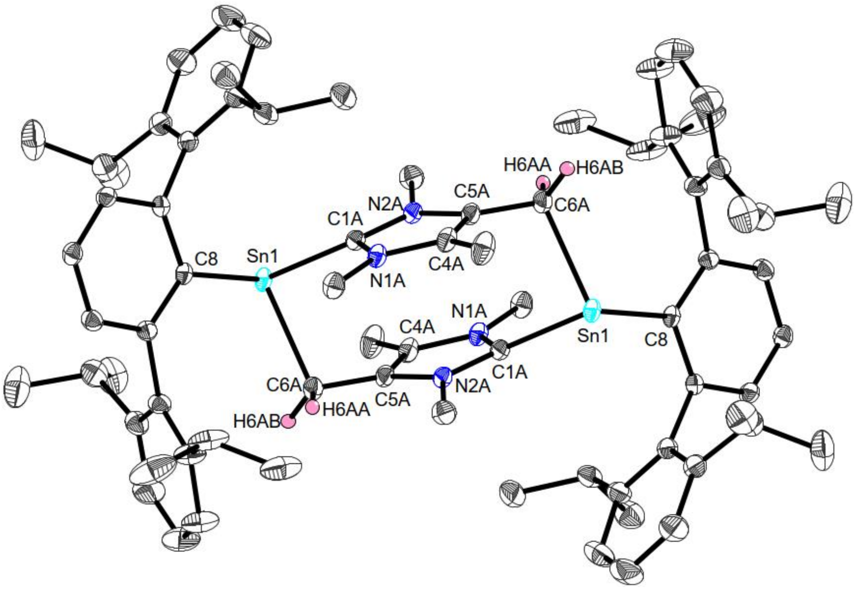

Figure S24. Molecular structure of C–H activation product **4b** (polymorph b) in the crystal. Thermal ellipsoids are drawn at the 50 % probability level (hydrogen atoms, except H6AA and H6AB and disorder have been omitted for clarity). Selected bond lengths (Å) and angles (deg): Sn1–C1A 2.3039(14), Sn1–C6A 2.3048(16), Sn1–C8 2.2364(13), C1A–Sn1–C6A , C1A–Sn1–C8 , C6A–Sn1–C8 .

Reaction of ^{Tipp}TerSn{N(SiMe$_3$)$_2$} (1c) with IMe$_4$ – Synthesis of ^{Tipp}TerSn(IMe$_4$){N(SiMe$_3$)$_2$} (2c) and of the C–H Activation Product 4c

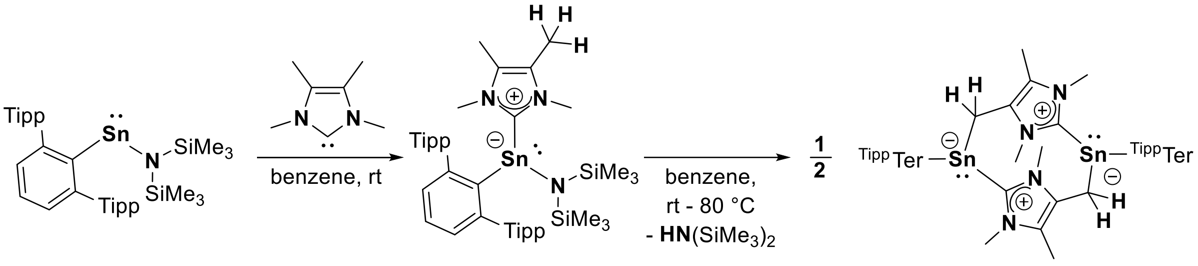

In a typical experiment, ^{Tipp}TerSn{N(SiMe$_3$)$_2$} (**1c**) (0.030 g, 0.039 mmol) and IMe$_4$ (0.005 g, 0.039 mmol) were dissolved in 0.5 mL of C_6D_6, resulting in an immediate colour change of the reaction mixture from orange to yellow due to the formation of ^{Tipp}TerSn(IMe$_4$){N(SiMe$_3$)$_2$} (**2c**). Over time, the amount of HN(SiMe$_3$)$_2$ increases and can be accelerated by heating the reaction mixture to 80 °C over a prolonged time. During prolonged heating a yellow crystalline material precipitates which was suitable for single crystal X-ray diffraction and was identified as the C–H activation product **4c**. These crystals were collected by filtration and dried under vacuum to give **4c** as a yellow powder.
For the isolation of ^{Tipp}TerSn(IMe$_4$){N(SiMe$_3$)$_2$} (**2c**), ^{Tipp}TerSn{N(SiMe$_3$)$_2$} (**1c**) (0.030 g, 0.039 mmol) and IMe$_4$ (0.005 g, 0.039 mmol) were dissolved in 1 mL of benzene, the reaction mixture was stirred for five minutes at room temperature, and subsequently, all volatile components were removed under vacuum. The yellow microcrystalline solid was stored at -30 °C.

Data of ^{Tipp}TerSn(IMe$_4$){N(SiMe$_3$)$_2$} (2c):
Yield: 0.034 g (0.038 mmol; 97%).
^{1}H NMR (400 MHz, C_6D_6, 298 K): δ = 0.16 (s, 18H, Si(CH$_3$)$_3$), 1.17 (d(br), $^3J_{H,H}$ = 6.7 Hz, 12H, *o*-CH(C*H*$_3$)$_2$-C$_6$H$_3$), 1.30 (d, $^3J_{H,H}$ = 6.9 Hz, 12H, *p*-CH(C*H*$_3$)$_2$-C$_6$H$_3$), 1.33 (s, 6H, NC$_q$CH$_3$), 1.36 (m(br), 12H, *o*-CH(C*H*$_3$)$_2$-C$_6$H$_3$), 2.87 (hept, $^3J_{H,H}$ = 7.1 Hz, 2H, *p*-C*H*(CH$_3$)$_2$-C$_6$H$_3$), 3.05 (s, 6H, NCH$_3$), 3.41 (hept, $^3J_{H,H}$ = 6.7 Hz, 4H, *o*-C*H*(CH$_3$)$_2$-C$_6$H$_3$), 7.13 (m(br), 4H, *m*-C*H*$_{Aryl}$-C$_6$H$_3$), 7.17-7.20 (m, 1H, *p*-CH$_{Aryl}$Sn), 7.27 (d, $^3J_{H,H}$ = 7.1 Hz, 2H, *m*-CH$_{Aryl}$Sn) ppm.
^{13}C{^{1}H} NMR (126 MHz, C_6D_6, 298 K): δ = 6.9 (Si(CH$_3$)$_3$), 8.1 (NC$_q\underline{C}$H$_3$), 23.8 (br, *o*-CH($\underline{C}$H$_3$)$_2$-C$_6$H$_3$), 24.6 (*p*-CH($\underline{C}$H$_3$)$_2$-C$_6$H$_3$), 26.3 (br, *o*-CH($\underline{C}$H$_3$)$_2$-C$_6$H$_3$), 31.1 (*o*-$\underline{C}$H(CH$_3$)$_2$-C$_6$H$_3$), 34.7 (*p*-$\underline{C}$H(CH$_3$)$_2$-C$_6$H$_3$), 35.0 (NCH$_3$), 120.9 (br, *m*-$\underline{C}$H$_{Aryl}$-C$_6$H$_3$), 124.8 (N$\underline{C}_q$CH$_3$), 125.4 (br, *p*-CH$_{Aryl}$Sn), 132.2 (*m*-CH$_{Aryl}$Sn), 147.2 (br, C$_{q,Aryl}$), 147.8 (br, C$_{q,Aryl}$), 148.0 (br, C$_{q,Aryl}$), 175.9 (C$_{q,Aryl}$Sn), 181.3 (C$_{NHC}$)* ppm.
* assigned by ^{1}H/^{13}C HMBC
6.2 (Si(CH$_3$)$_3$), 23.1 (*o*-CH($\underline{C}$H$_3$)$_2$-C$_6$H$_3$), 24.3 (*p*-CH($\underline{C}$H$_3$)$_2$-C$_6$H$_3$), 27.3 (*o*-CH($\underline{C}$H$_3$)$_2$-C$_6$H$_3$), 31.4 (*o*-$\underline{C}$H(CH$_3$)$_2$-C$_6$H$_3$), 34.8 (*p*-$\underline{C}$H(CH$_3$)$_2$-C$_6$H$_3$), 122.2 (*m*-$\underline{C}$H$_{Aryl}$-C$_6$H$_3$), 126.9 (*p*-CH$_{Aryl}$Sn), 131.7 (*m*-CH$_{Aryl}$Sn), 135.1 (C$_{q,ipso,Tipp}$), 144.9 (*o*-C$_{q,Aryl}$Sn), 147.5 (*o*-$\underline{C}_{q,Aryl}$-C$_6$H$_3$), 149.7 (*p*-$\underline{C}_{q,Aryl}$-C$_6$H$_3$), 185.5 (C$_{q,Aryl}$Sn) ppm.
^{119}Sn{^{1}H} NMR (149 MHz, C_6D_6, 298 K): δ = -20.0 ppm.
MS: No meaningful results could be obtained using LIFDI, ESI and CI MS.

Data of the C–H activation product 4c:
Yield: 0.011 g (0.008 mmol; 41%). **Note**: Due to the low solubility in common organic solvents preventing sufficient NMR analysis, the yield has to be taken with caution.
MS (LIFDI): m/z calcd. for $C_{86}H_{120}N_4Sn$: 1448.7557; found: 1448.4.
EA: Anal. calcd. for $C_{86}H_{120}N_4Sn_2$: C, 71.37; H, 8.36; N, 3.87; Found: C, 71.20; H, 8.39; N, 3.77.

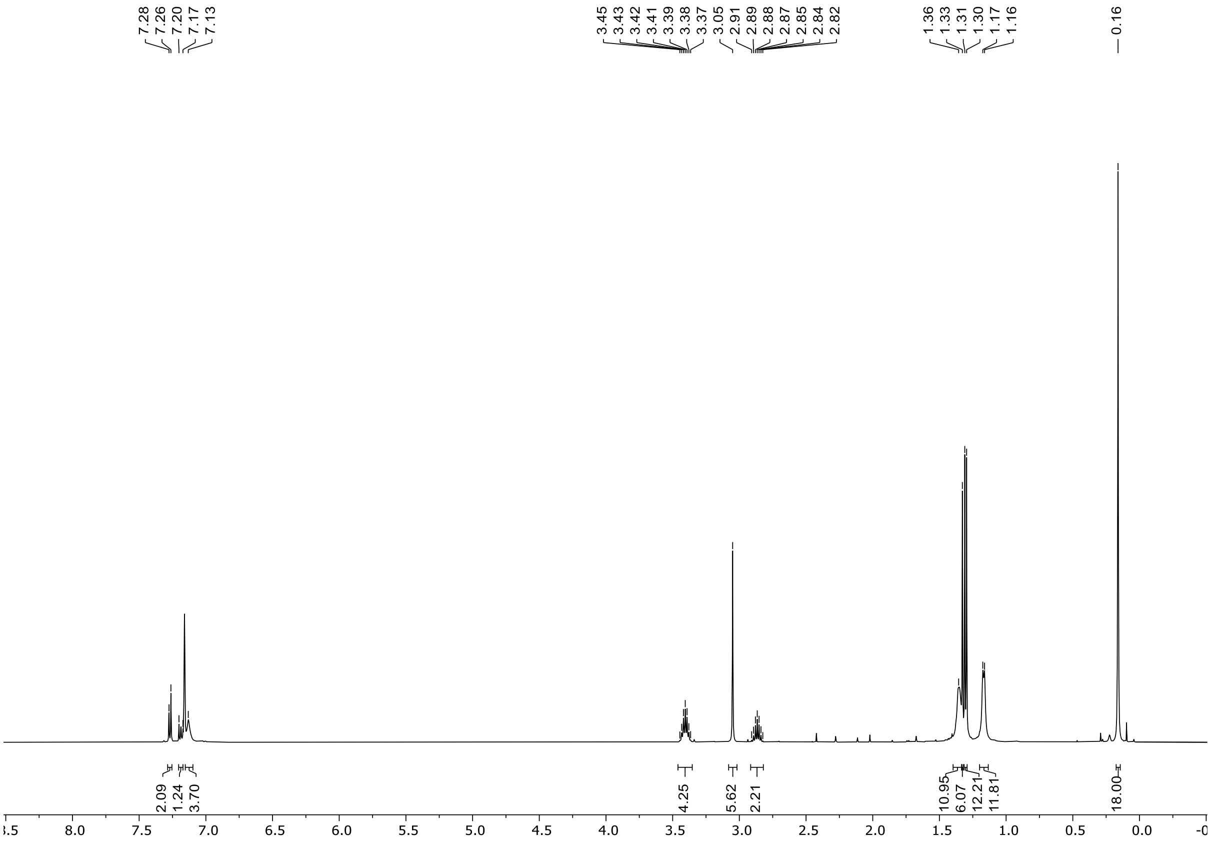

Figure S25. ^{1}H NMR spectrum of ^{Tipp}TerSn(IMe$_4$){N(SiMe$_3$)$_2$} (**2c**) (400 MHz, C_6D_6, 298 K).

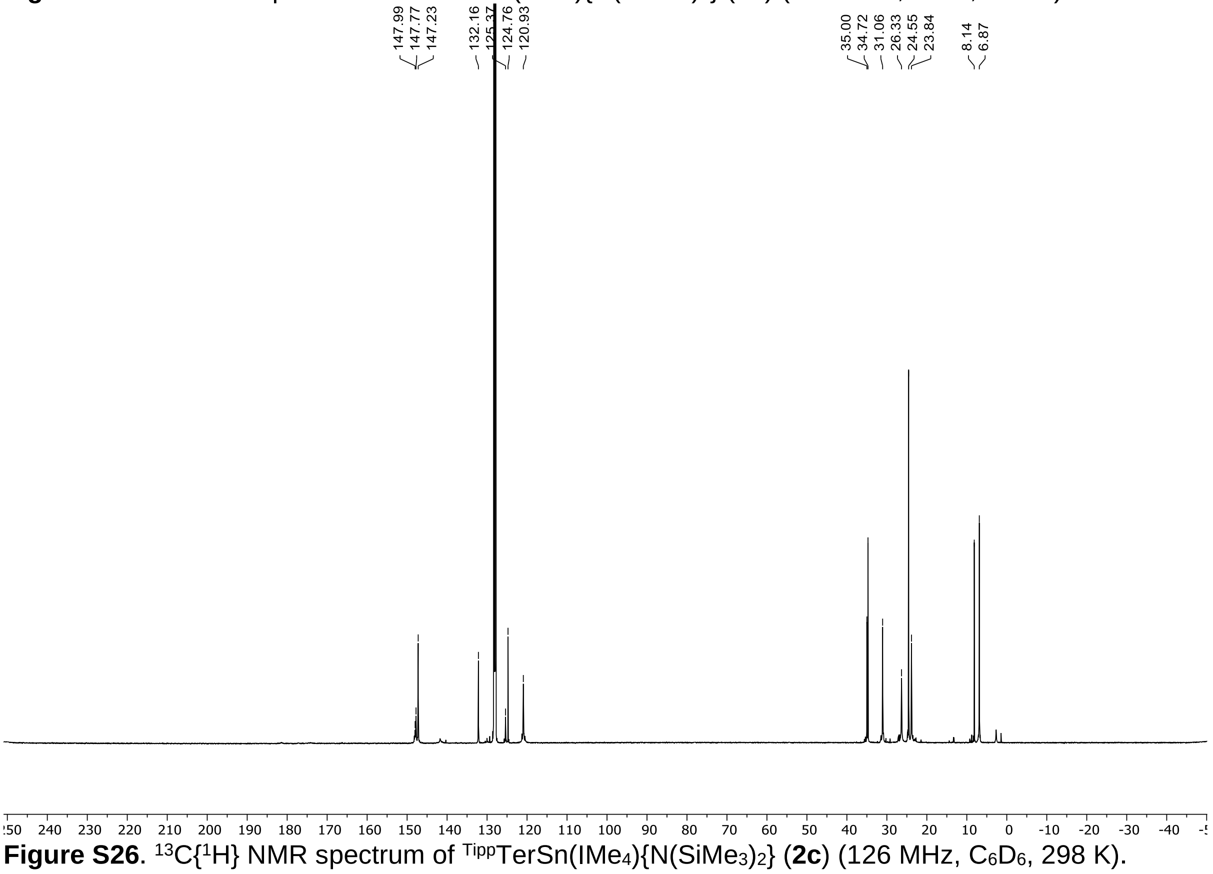

Figure S26. $^{13}C\{^{1}H\}$ NMR spectrum of ^{Tipp}TerSn(IMe$_4$){N(SiMe$_3$)$_2$} (**2c**) (126 MHz, C_6D_6, 298 K).

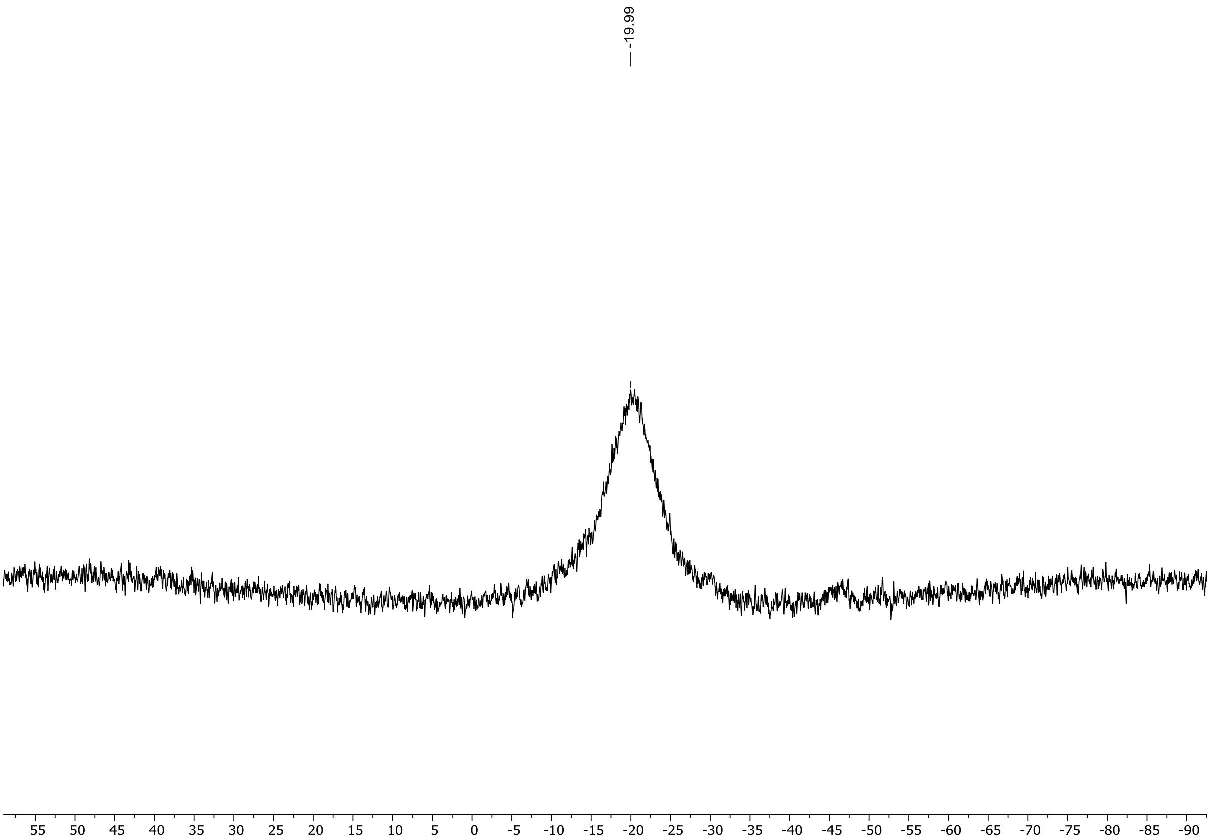

Figure S27. $^{119}Sn\{^1H\}$ NMR spectrum of $^{Tipp}TerSn(IMe_4)\{N(SiMe_3)_2\}$ (**2c**) (149 MHz, C_6D_6, 298 K).

Reaction Monitoring and Isolation of 4c

Following the procedure described above, $^{Tipp}TerSn\{N(Si(CH_3)_3)_2\}$ (**1c**) (0.030 g, 0.039 mmol) was dissolved in 0.5 mL of C_6D_6. Figures S28 and S29 (bottom) display the respective ^1H NMR spectrum of **1c**, along with an excerpt of the high-field region. In a glove box, IMe_4 (0.005 g, 0.039 mmol) was added to the solution, and the reaction mixture was subsequently analysed by ^1H NMR spectroscopy. Figure S18 (top left) shows the solution in a J. Young NMR tube immediately after the addition of IMe_4, highlighting the colour change from orange (characteristic of **1c**) to yellow (indicative of the formation of $^{Tipp}TerSn(IMe_4)\{N(Si(CH_3)_3)_2\}$ (**2c**)). The corresponding ^1H NMR spectrum and a high-field region excerpt of this solution are presented in Figures S28 and S29 (second from bottom), consistent with the formation of **2c**. After two hours at room temperature, Figures S28 and S29 (middle) show the corresponding ^1H NMR spectrum, revealing an increased formation of $HN(Si(CH_3)_3)_2$ (δ^1H = 0.10 ppm). The reaction mixture was then left undisturbed over the weekend, and Figures S27 and S28 (second from top) show the ^1H NMR spectra obtained at this stage, alongside the observation of a colourless crystalline precipitate. Upon heating the reaction mixture overnight, **2c** was fully consumed, and the formation of $HN(Si(CH_3)_3)_2$ was complete. Figures S28 and S29 (top) display the corresponding ^1H NMR spectrum of this final stage. The solution was transferred to a glove box and slightly concentrated under vacuum, leading to the further precipitation of **4c** as a colourless crystalline solid.

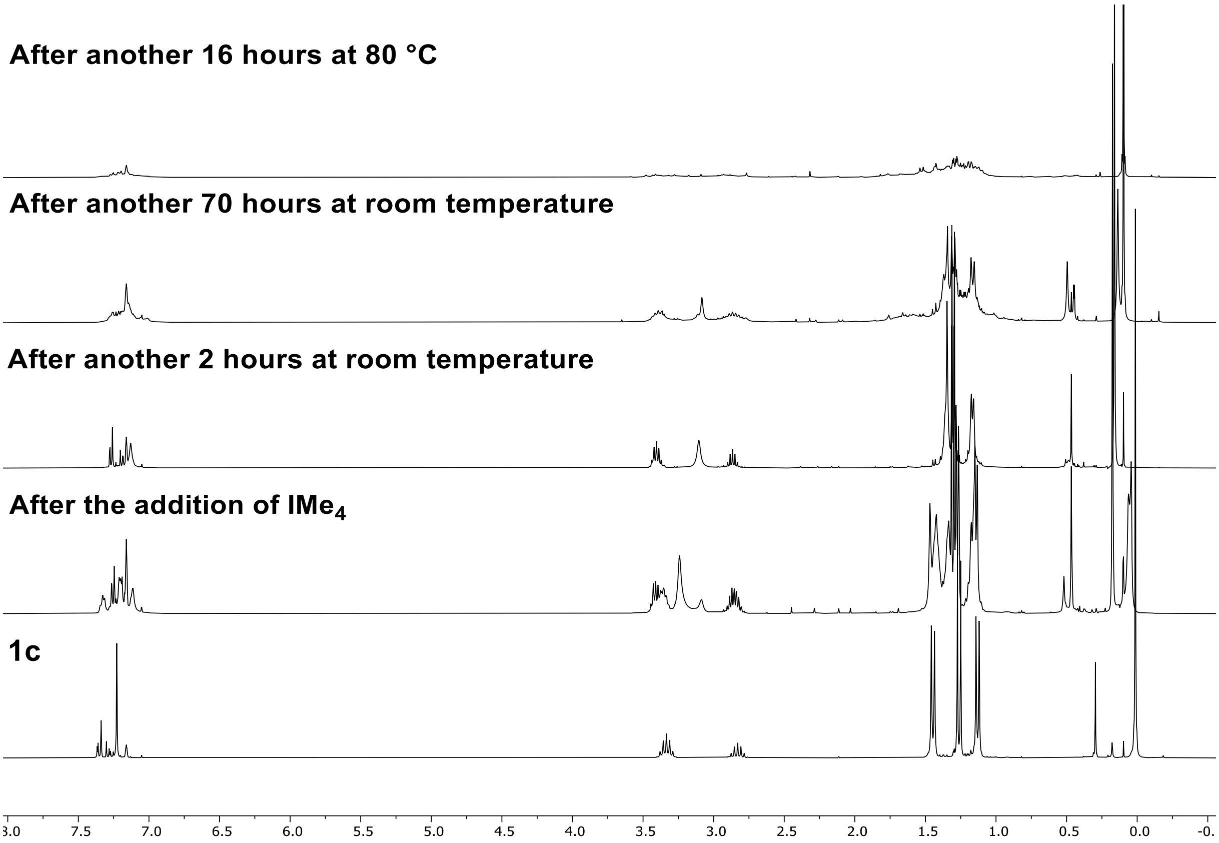

Figure S28. Monitoring of the reaction of ^{Tipp}TerSn{N(Si(CH$_3$)$_3$)$_2$} (**1c**) with IMe$_4$ to stepwise yield ^{Tipp}TerSn(IMe$_4$){N(Si(CH$_3$)$_3$)$_2$} (**2c**) and **4c** (400 MHz, C_6D_6, 298 K).

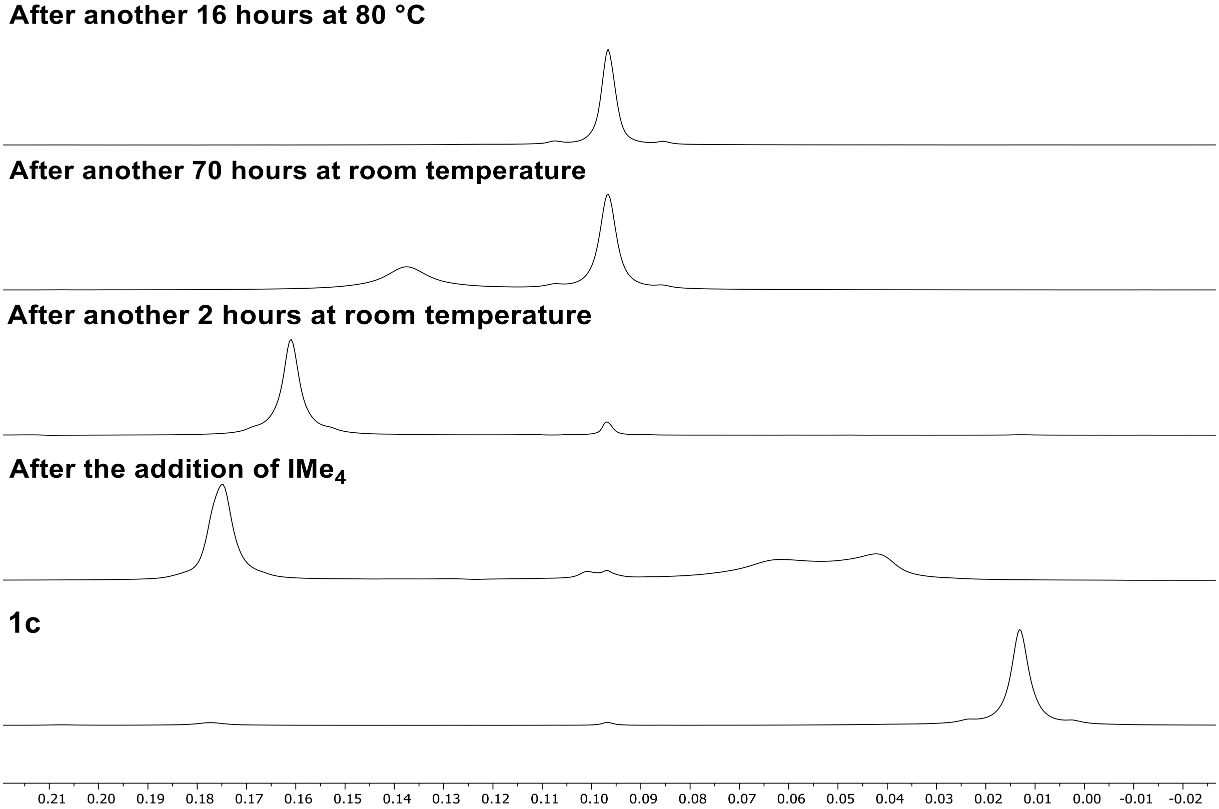

Figure S29. Excerpt of the high field region of Figure S27 (400 MHz, C_6D_6, 298 K).

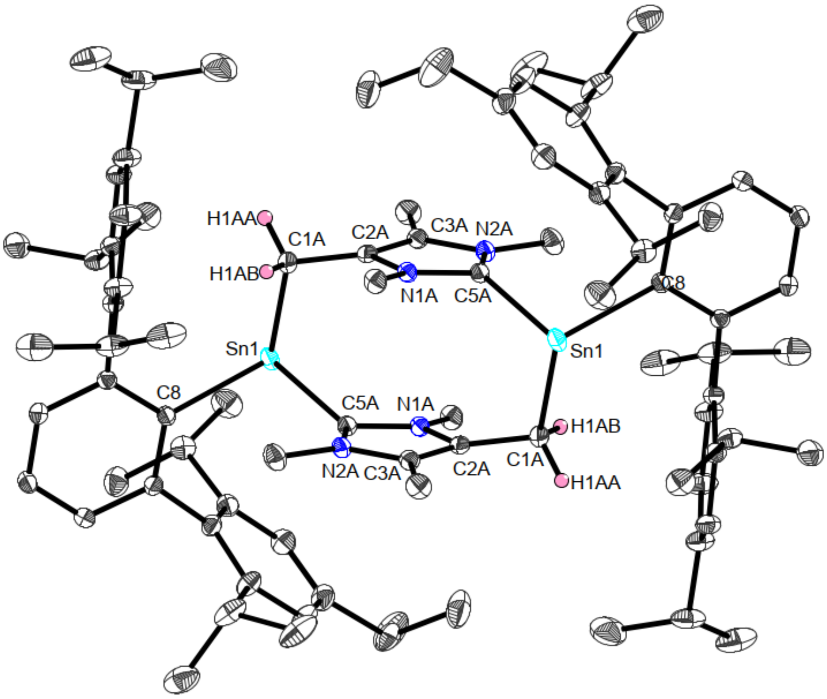

Figure S30. Molecular structure of C–H activation product **4c** in the crystal. Thermal ellipsoids are drawn at the 50 % probability level (hydrogen atoms, except H1AA and H1AB, lattice solvent and disorder have been omitted for clarity). Selected bond lengths (Å) and angles (deg): Sn1–C1A 2.2715(15), Sn1–C5A 2.3032(15), Sn1–C8 2.2444(11), C1A–Sn1–C5A 88.83(5), C1A–Sn1–C8 109.94(5), C5A–Sn1–C8 97.71(5).

Reaction of ^{Mes}TerSn{N(Si(CH$_3$)$_3$)$_2$} (1a) with I^{i}Pr$_2$Me$_2$ – Synthesis of the C–H Activation Product 5a

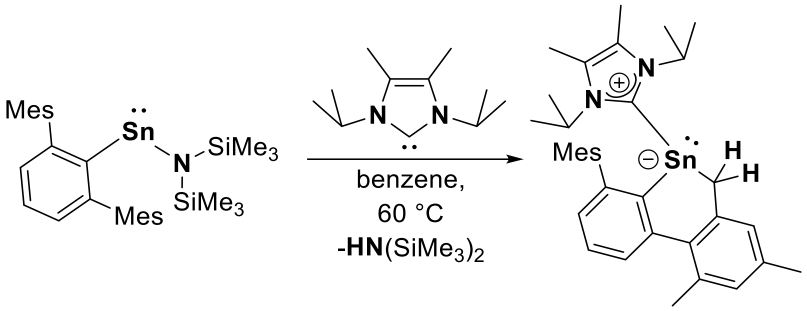

In a typical experiment, ^{Mes}TerSn{N(Si(CH$_3$)$_3$}$_2$ (**1a**) (0.030 g, 0.051 mmol) and I^{i}Pr$_2$Me$_2$ (0.009 g, 0.051 mmol) were dissolved in 0.5 mL of C_6D_6 or benzene. Subsequent ^{1}H NMR analysis of the respective sample in C_6D_6 revealed no occurring reaction at room temperature. Heating the reaction mixture to 60 °C results in the formation of HN(SiMe$_3$)$_2$, as evident by its characteristic ^{1}H NMR chemical shift (δ^1H = 0.10 ppm). Heating was continued until both starting materials were completely consumed. All volatile components were removed under vacuum and the remaining solid was recrystallized from *n*-hexane at -30 °C to give the C–H activation product **5a** as a yellow crystalline solid. These crystals were suitable for single crystal X-ray diffraction.

Yield: 0.022 g (0.036 mmol; 71 %).
^{1}H NMR (400 MHz, C_6D_6, 298 K): δ = 0.96 (d, $^3J_{H,H}$ = 7.0 Hz, 6H, CH(CH_3)$_2$), 1.09 (d, $^3J_{H,H}$ = 7.1 Hz, 6H, CH(CH_3)$_2$), 1.33 (s, 6H, NC$_q$CH$_3$), 2.09 (s, 3H, CH$_3$), 2.25 (s, 3H, CH$_3$), 2.29 (s, 3H, CH$_3$), 2.33 (d, $^3J_{H,H}$ = 10.2 Hz, 1H, CH$_2$), 2.43 (s, 3H, CH$_3$), 2.47 (s, 3H, CH$_3$), 2.58 (d, $^3J_{H,H}$ = 10.4 Hz, 1H, CH$_2$), 4.91 (hept, $^3J_{H,H}$ = 7.1 Hz, 2H, C*H*(CH$_3$)$_2$), 6.52 (s(br), 1H, CH$_{Aryl}$), 6.76 (s(br), 1H, CH$_{Aryl}$), 6.96 (s(br), 1H, CH$_{Aryl}$), 7.00 (s(br), 1H, CH$_{Aryl}$), 7.10-7.12 (m, 1H, CH$_{Aryl}$), 7.39-7.43 (m, 1H, CH$_{Aryl}$), 7.62-7.64 (m, 1H, CH$_{Aryl}$) ppm.
^{13}C{^{1}H} NMR (101 MHz, C_6D_6, 298 K): δ = 21.3 (CH$_3$), 21.4 (CH$_3$), 21.8 (CH($\underline{C}$H$_3$)$_2$), 22.1 (CH($\underline{C}$H$_3$)$_2$, CH$_3$), 22.4 (CH$_3$), 23.3 (CH$_3$), 34.0 (NC$_q\underline{C}$H$_3$), 52.6 ($\underline{C}$H(CH$_3$)$_2$), 124.4 (CH$_{Aryl}$), 125.4 (N$\underline{C}_q$CH$_3$), 125.6 (CH$_{Aryl}$), 127.1 (CH$_{Aryl}$), 127.25 (CH$_{Aryl}$), 127.35 (CH$_{Aryl}$), 127.7 (CH$_{Aryl}$)*, 128.8 (CH$_{Aryl}$), 133.9 (C$_{q,Aryl}$), 134.7 (C$_{q,Aryl}$), 135.5 (C$_{q,Aryl}$), 137.0 (C$_{q,Aryl}$), 139.2 (C$_{q,Aryl}$), 145.5 (C$_{q,Aryl}$), 145.54 (C$_{q,Aryl}$), 148.6 (C$_{q,Aryl}$), 149.5 (C$_{q,Aryl}$), 167.4 (C$_{q,Aryl}$), 176.6 (C$_{q,Aryl}$Sn) ppm. **Note**: The signal of the carbene carbon atom could not be observed.
* = overlap with C_6D_6 signal
^{119}Sn{^{1}H} NMR (149 MHz, C_6D_6, 298 K): δ = -164.4 ppm.
MS (LIFDI): m/z calcd. for $C_{35}H_{42}N_2Sn$: 612.2527; found: 612.4.
EA: Anal. calcd. for $C_{41}H_{63}N_3Si_2Sn$: C, 68.75; H, 7.25; N, 4.58; Found: C, 68.84; H, 7.32; N, 4.41.

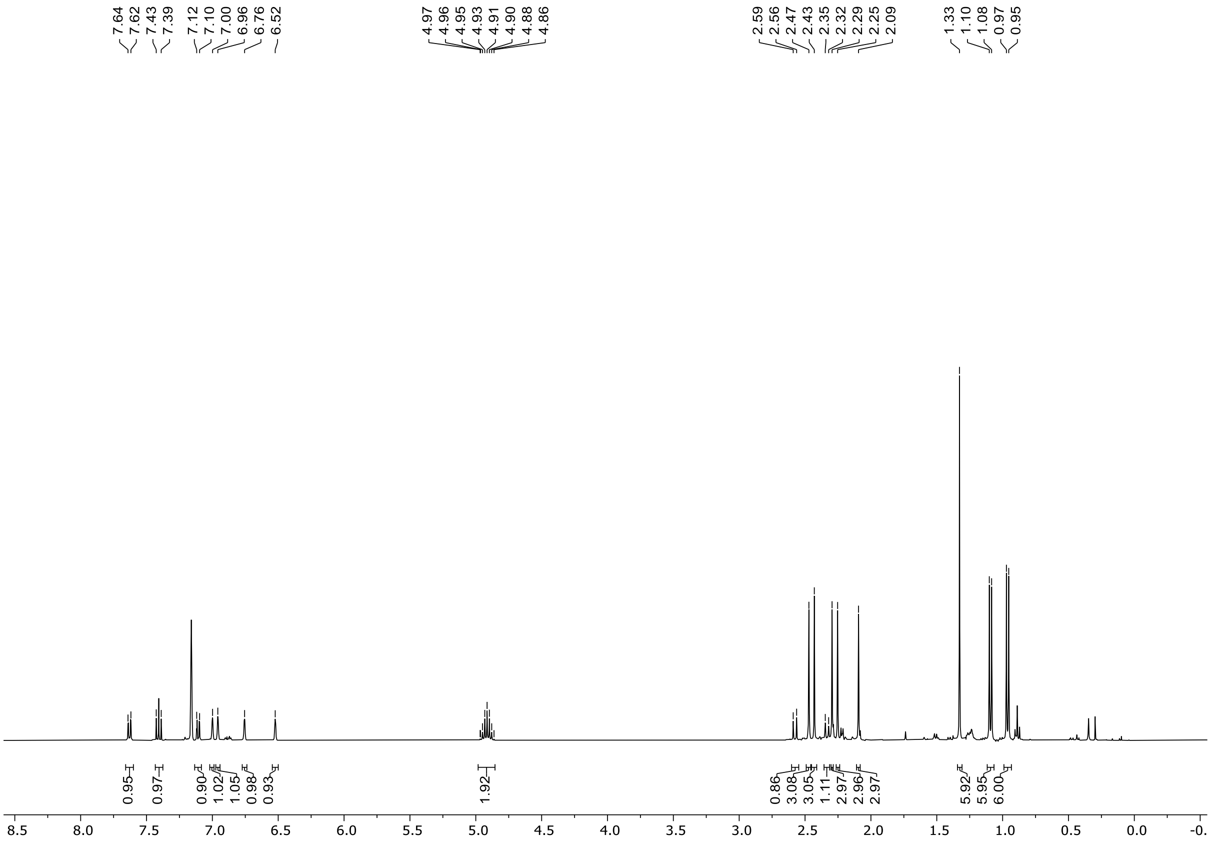

Figure S31. ^{1}H NMR spectrum of C–H activation product (**5a**) (400 MHz, C_6D_6, 298 K).

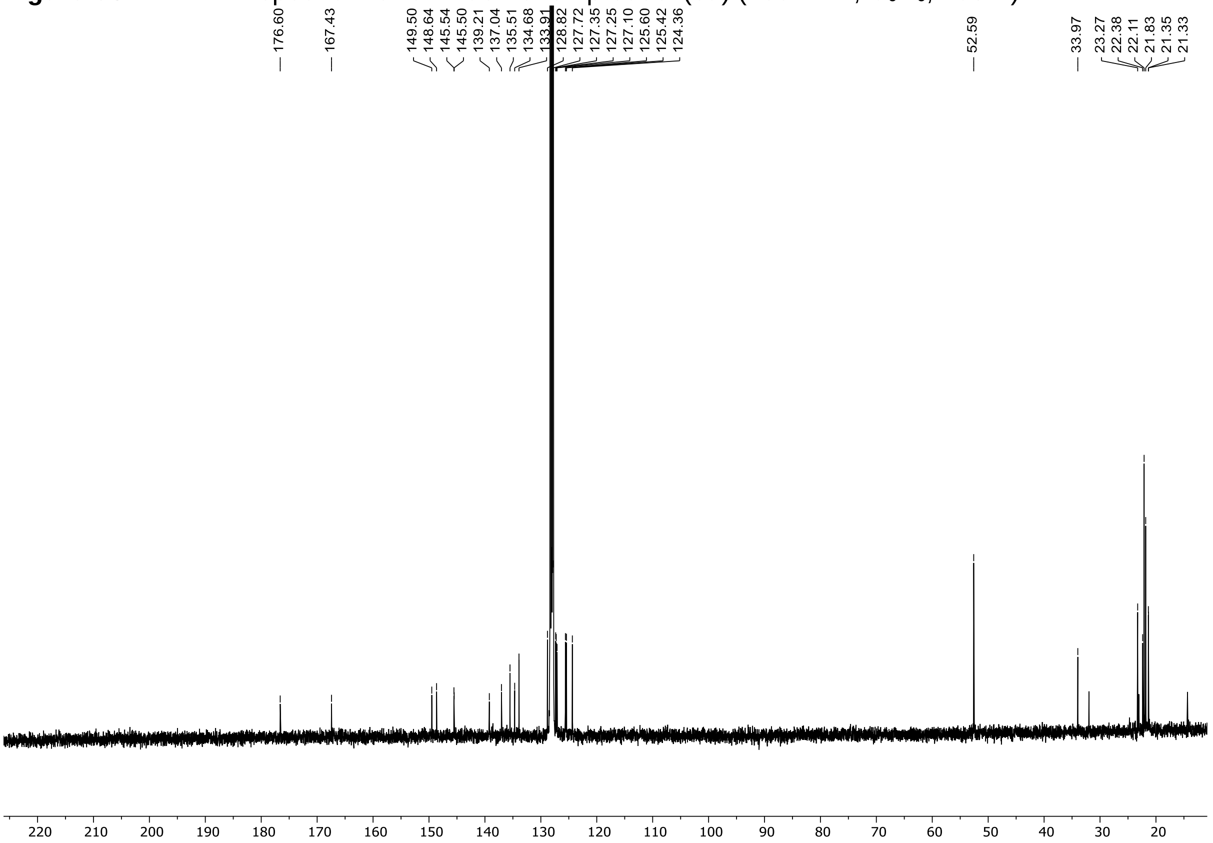

Figure S32. $^{13}C\{^{1}H\}$ NMR spectrum of C–H activation product (**5a**) (126 MHz, C_6D_6, 298 K).

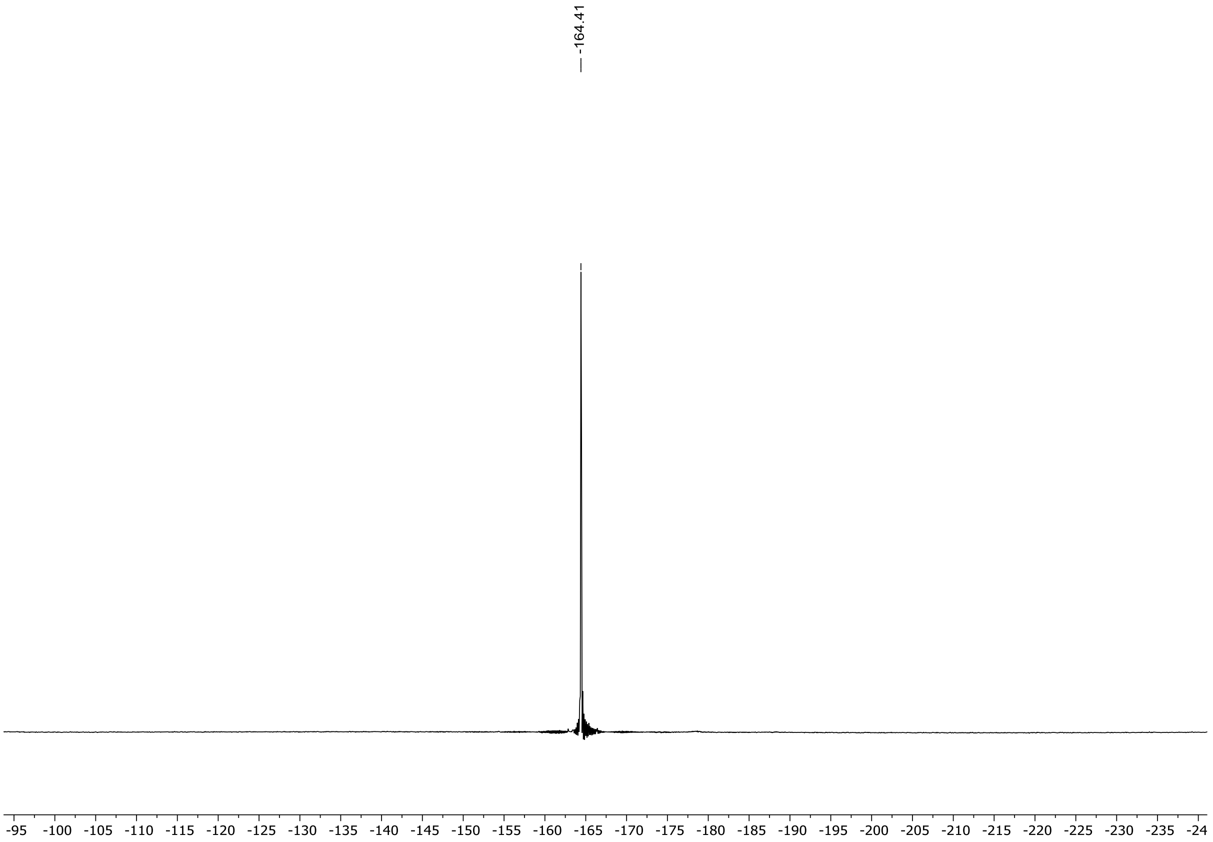

Figure S33. $^{119}Sn\{^{1}H\}$ NMR spectrum of C–H activation product (**5a**) (149 MHz, C_6D_6, 298 K).

Reaction of $^{Dipp}TerSn\{N(Si(CH_3)_3)_2\}$ (1b) with $I^iPr_2Me_2$ – Synthesis of the doubly C–H Activation Product 6b

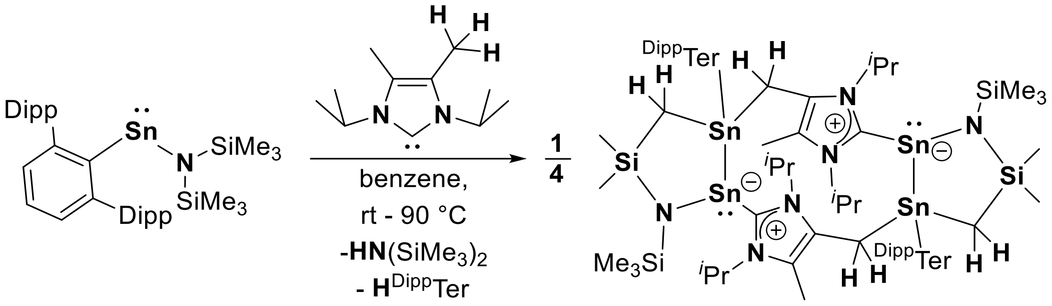

In a typical experiment, $^{Dipp}TerSn\{N(Si(CH_3)_3\}_2$ (**1a**) (0.030 g, 0.044 mmol) and $I^iPr_2Me_2$ (0.008 g, 0.044 mmol) were dissolved in 0.5 mL of C_6D_6 or benzene. Subsequent ^1H NMR analysis of the respective sample in C_6D_6 revealed no occurring reaction at room temperature. Heating the reaction mixture to 90 °C results in the formation of $HN(SiMe_3)_2$, as evident by its characteristic ^1H NMR chemical shift (δ^1H = 0.10 ppm), and also the formation of $H^{Dipp}Ter$ can be observed (Figure S26). Heating was continued until both starting materials were completely consumed (approximately three days). During the course of the reaction, the formation of a yellow crystalline material can be observed. These crystals were suitable for single crystal X-ray diffraction, revealing the formation of the doubly C–H activation product **6b**. The yellow crystalline material can be separated by filtration and was dried under vacuum to give **6b** as a yellow solid.

Yield: 0.008 g (0.004 mmol; 37%). **Note**: Due to the low solubility in common organic solvents preventing sufficient NMR analysis, the yield has to be taken with caution.
MS: No meaningful results could be obtained using LIFDI, ESI and CI MS.
EA: Anal. calcd. for $C_{94}H_{146}N_6Si_4Sn_4$: C: 57.98; H: 7.56; N: 4.32; Found: C: 57.74; H: 7.56; N: 4.24.

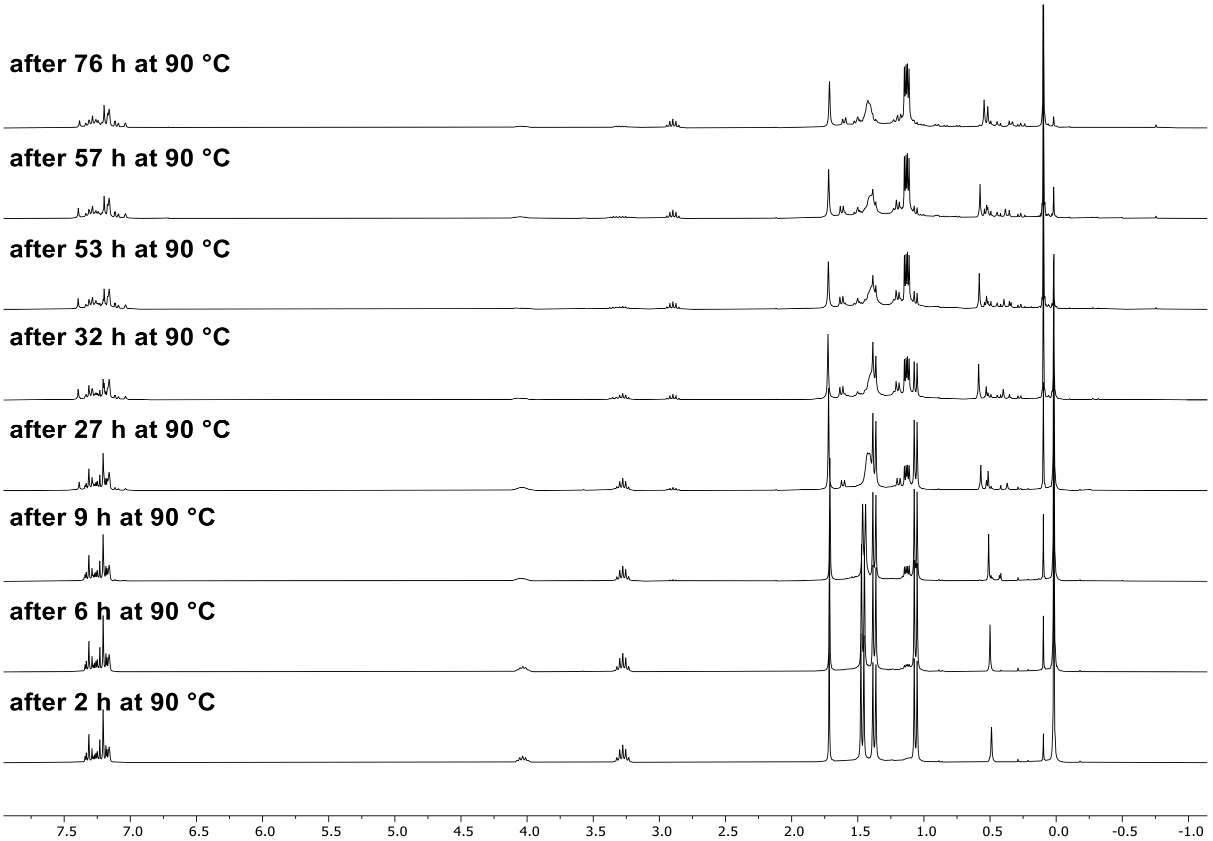

Figure S34. Monitoring of the reaction of $^{Dipp}TerSn\{N(SiMe_3)_2\}$ (**1b**) with $IiPr_2Me_2$ by ^1H NMR spectroscopy (300 MHz, C_6D_6, 298 K); 0.10 ppm: $HN(SiMe_3)_2$; 1.11-1.15 and 2.90 ppm: characteristic signals of $H^{Dipp}Ter$.

Reaction of ^{Tipp}TerSn{N(Si(CH$_3$)$_3$)$_2$} (1c) with I^{i}Pr$_2$Me$_2$ – Synthesis of the doubly C–H Activation Product 6c

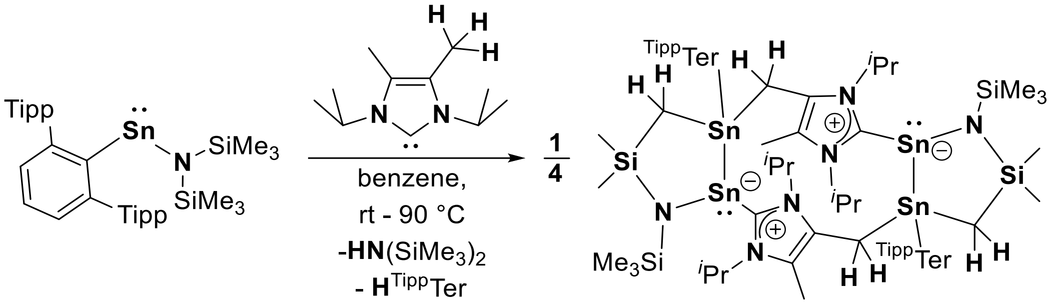

In a typical experiment, ^{Tipp}TerSn{N(Si(CH$_3$)$_3$}$_2$ (**1c**) (0.030 g, 0.039 mmol) and I^{i}Pr$_2$Me$_2$ (0.007 g, 0.039 mmol) were dissolved in 0.5 mL of C_6D_6 or benzene. Subsequent ^{1}H NMR analysis of the respective sample in C_6D_6 revealed no occurring reaction at room temperature. Heating the reaction mixture to 100 °C results in the formation of HN(SiMe$_3$)$_2$, as evident by its characteristic ^{1}H NMR chemical shift (δ^1H = 0.10 ppm), and also the formation of H^{Tipp}Ter can be observed (Figure S27). Heating was continued until both starting materials were completely consumed (approximately four days). During the course of the reaction, the formation of a yellow crystalline material can be observed. These crystals were suitable for single crystal X-ray diffraction, revealing the formation of the doubly C–H activation product **6c**. The yellow crystalline material can be separated by filtration and was dried under vacuum to give **6c** as a yellow solid.

Yield: 0.007 g (0.003 mmol; 34%). **Note**: Due to the low solubility in common organic solvents preventing sufficient NMR analysis, the yield has to be taken with caution.
MS: No meaningful results could be obtained using LIFDI, ESI and CI MS.
EA: Anal. calcd. for $C_{106}H_{170}N_6Si_4Sn_4$: C: 60.18; H: 8.10; N: 3.97; Found: C: 59.96; H: 7.72; N: 3.43.

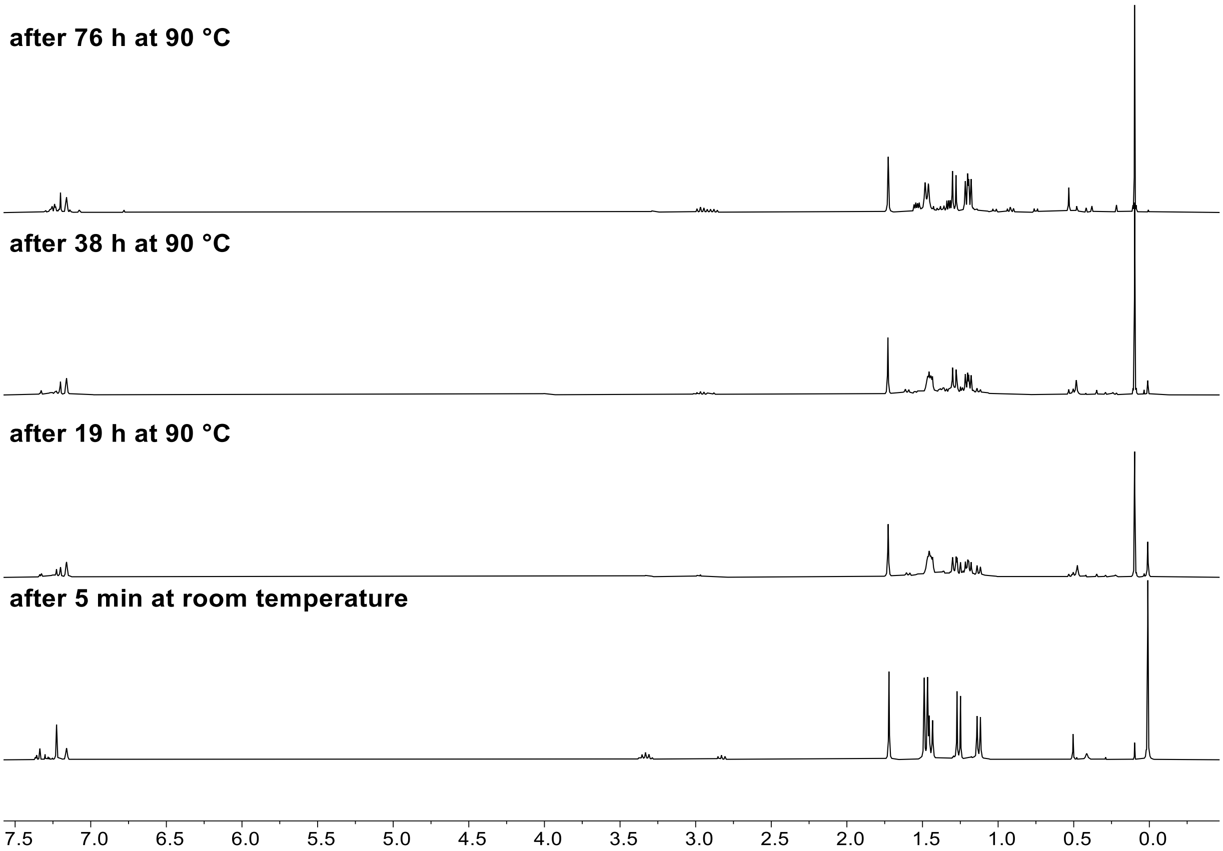

Figure S35. Monitoring of the reaction of $^{Tipp}TerSn\{N(SiMe_3)_2\}$ (**1c**) with $IiPr_2Me_2$ by ^{1}H NMR spectroscopy (300 MHz, C_6D_6, 298 K); 0.10 ppm: $HN(SiMe_3)_2$.

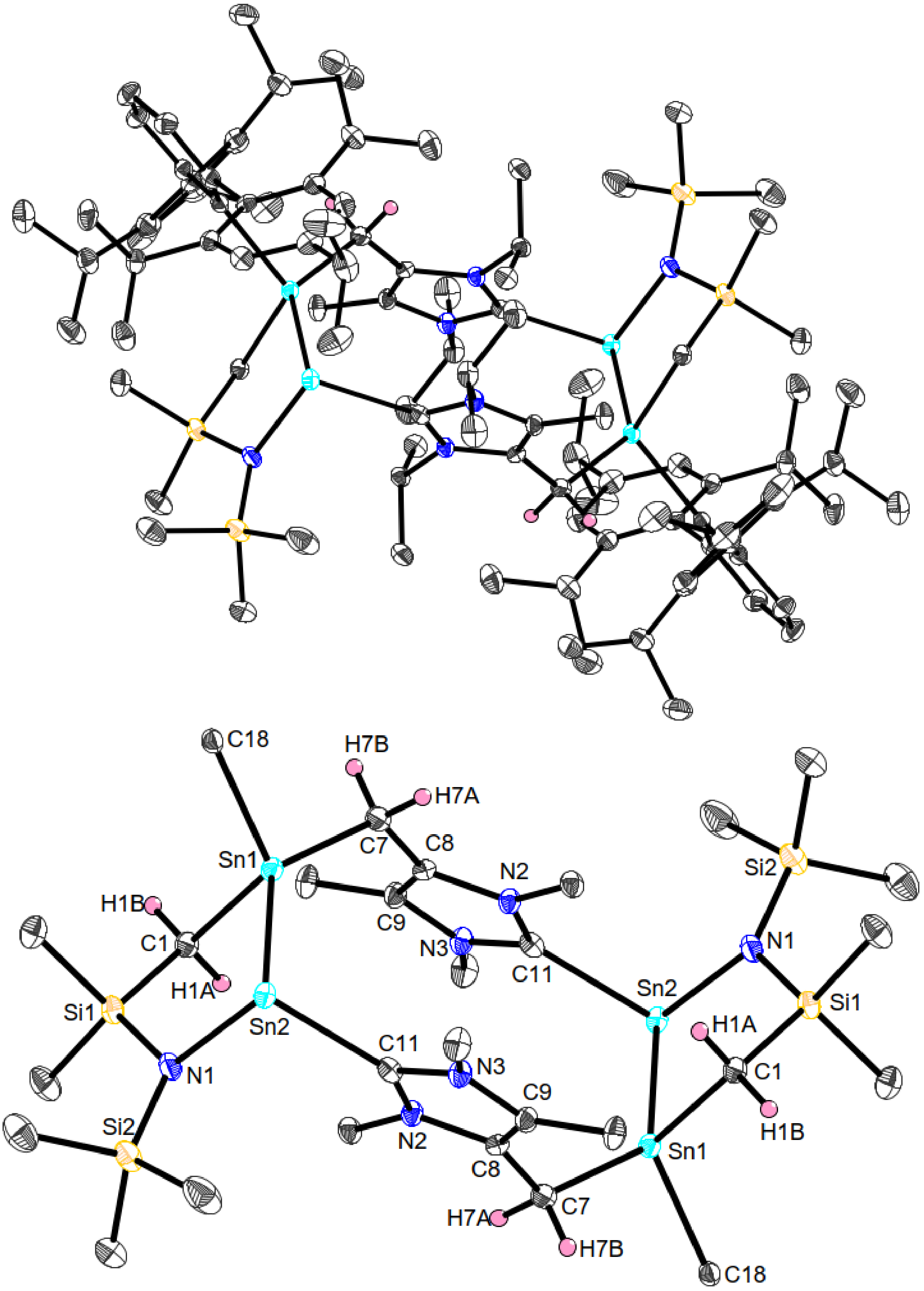

Figure S36. Molecular structure of the doubly C–H activation product **6c** determined by single crystal X-ray crystallography. Anisotropic displacement parameters are drawn at the 50% probability level (hydrogen atoms, except H1A, H1B, H7A and H7B, terphenyl substituents and part of the *N*-*iso*-propyl groups have been omitted or reduced for clarity). Selected bond lengths (Å) and angles (deg): Sn1–Sn2 2.9058(7), Sn1–C1 2.176(3), Sn1–C7 2.221(3), Sn1–C18 2.225(3), Sn2–N1 2.188(3), Sn2–C11 2.351(3), N1–Sn2–Sn1 87.79(7), Sn1–Sn2–C11 95.06(7), N1–Sn2–C11 98.33(10), C1–Sn1–Sn2 93.28(8), C1–Sn1–C7 107.30(11), C1–Sn1–C18 109.80(11), Sn2–Sn1–C7 133.48(8), Sn2–Sn1–C18 116.14(8), C7–Sn1–C18 95.80(11).

Crystallographic Details

Suitable crystals were mounted on a MiTeGen micromount with perfluoroether oil. Single crystal X-ray data were collected from shock-cooled single crystals at 100.00 K on a Bruker D8 VENTURE dual wavelength Mo/Ag four-circle diffractometer or a Bruker D8 Venture dual wavelength Mo/Cu four-circle diffractometer, both with microfocus sealed X-ray tubes using mirror optics as monochromators and Bruker PHOTON III detectors. The diffractometer was equipped with an Oxford Cryostream 800 low temperature device and used MoK_{α} radiation (λ = 0.71073 Å). All data were integrated with SAINT and a numerical absorption correction using SADABS was applied.[5,6] The structure was solved by dual methods using XT and refined by full-matrix least-squares methods against F^2 by XL using Olex2.[7-9] Some atoms were refined using isotropic displacement parameters. All pther non-hydrogen atoms were refined with anisotropic displacement parameters. All C-bound hydrogen atoms were refined isotropic on calculated positions using a riding model with their U_{iso} values constrained to 1.5 times the U_{eq} of their pivot atoms for terminal sp^3 carbon atoms and 1.2 times for all other carbon atoms. Disordered moieties were refined using bond lengths restraints and displacement parameter restraints. Crystallographic data for the structural analyses have been deposited with the Cambridge Crystallographic Data Centre[10] under reference numbers 2386607–2386614. Copies of this information may be obtained free of charge from The Director, CCDC, 12 Union Road, Cambridge CB2 1EZ, UK (Fax: +44-1223-336033; email: deposit@ccdc.cam.ac.uk or http://www.ccdc.cam.ac.uk). The reports and the CIF file were generated using FinalCif.[11]

Refinement details, asymmetric units, pictures of the crystals used for data collection and further information:

2a: After the model was completed, a singular density peak persists, for which no chemically reasonable explanation can be given. It was attributed to an alternative Sn position and may represent the only visible aspect of a broader disorder with very low occupancies.

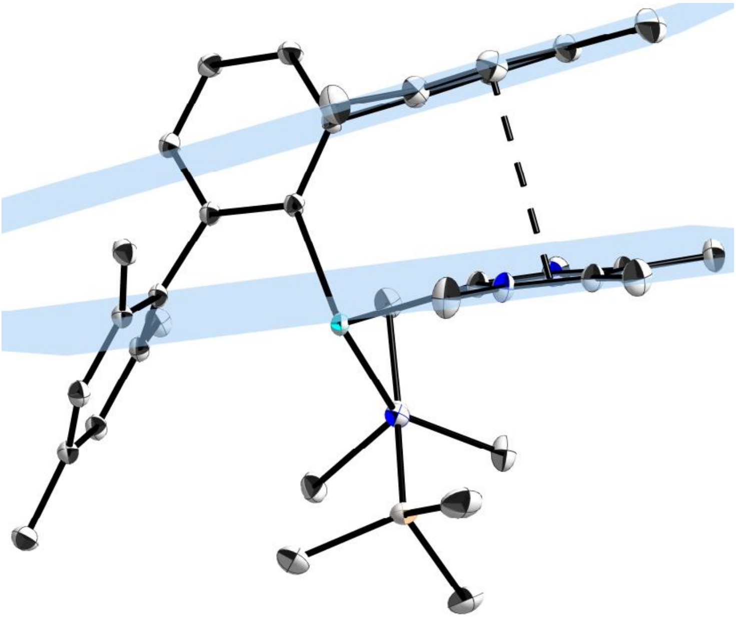

Figure S37. Visualization of the π–π-interactions in **2a**.

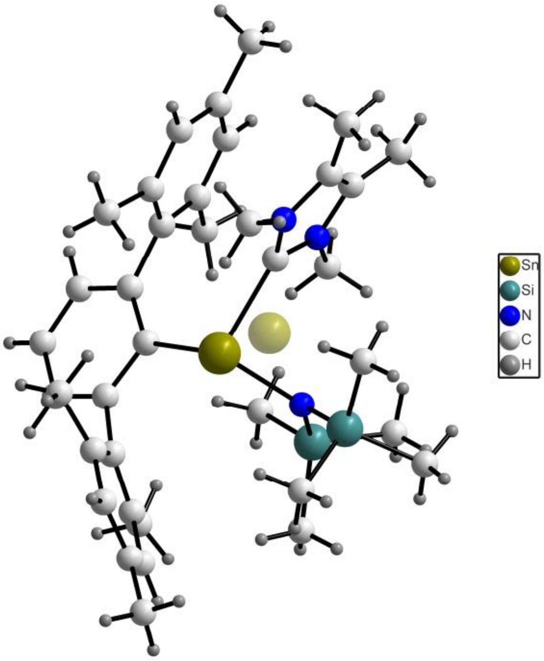

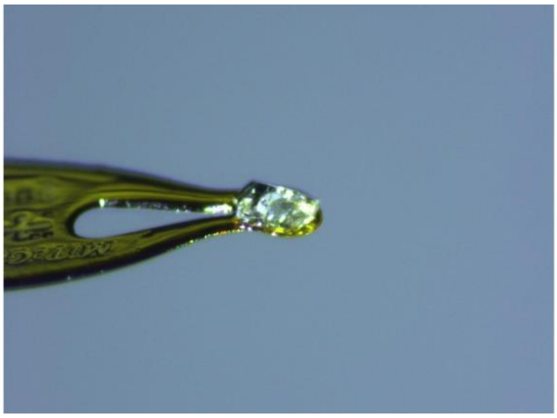

Figure S38. Left: Asymmetric unit of **2a**; Right: Picture of the crystal used for data collection.

3a: The sum of occupancies of the disordered benzenes might not be integer, so the SUMP command was commented out.

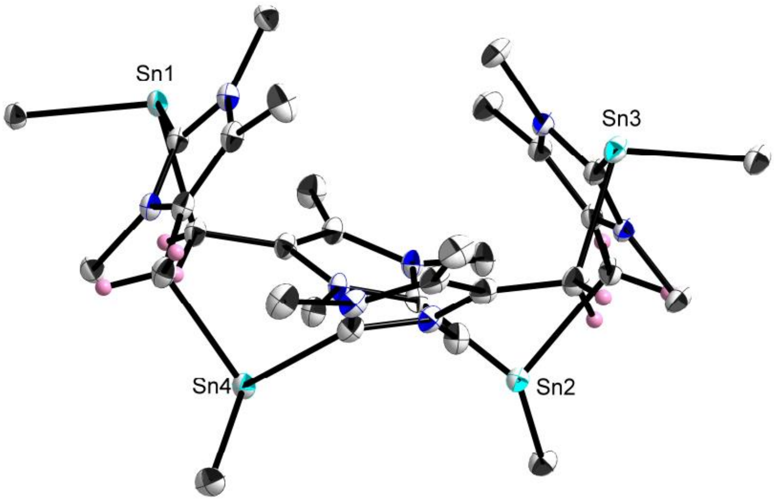

Figure S39. Visualization of the shape of the 20-membered macrocycle **3a**.

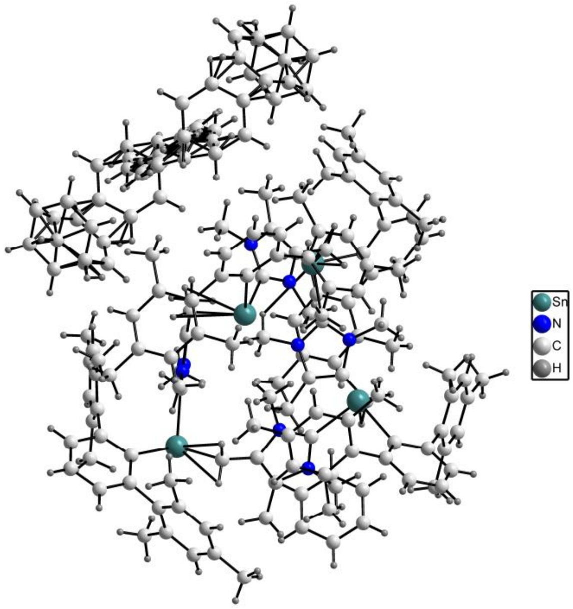

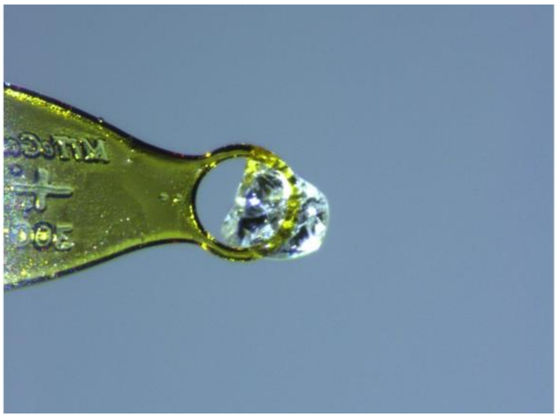

Figure S40. Left: Asymmetric unit of **3a**; Right: Picture of the crystal used for data collection.

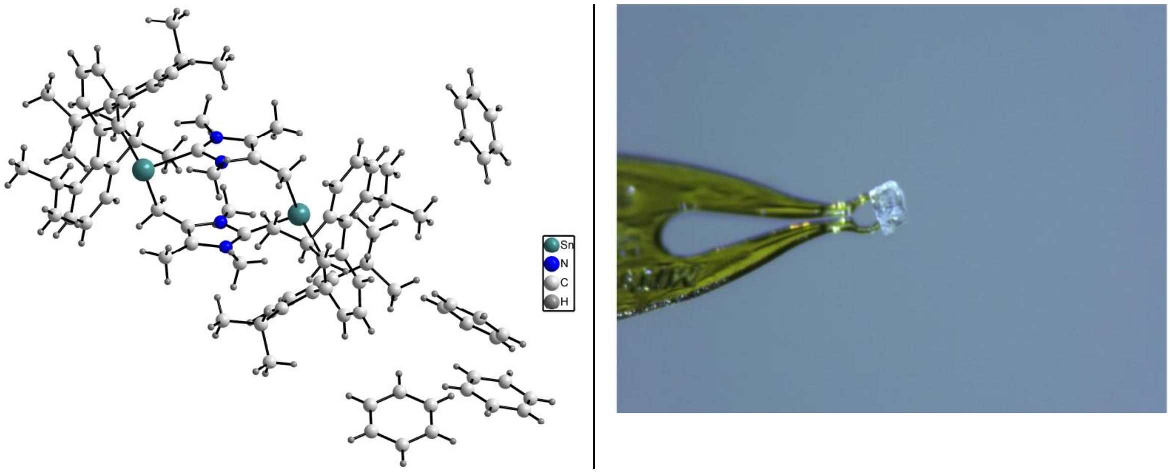

Figure S41. Left: Asymmetric unit of **4b** (polymorph a); Right: Picture of the crystal used for data collection.

4b (polymorph b): A minor disorder position for the bridging NHC fragment is clearly evident in the residual difference density. Due to its low occupancy, the ADPs of PART 2 are unstable, with isotropic refinement resulting in non-positive definite values for some atoms. Consequently, the U_{iso} of the minor part is fixed at a value of 0.04.

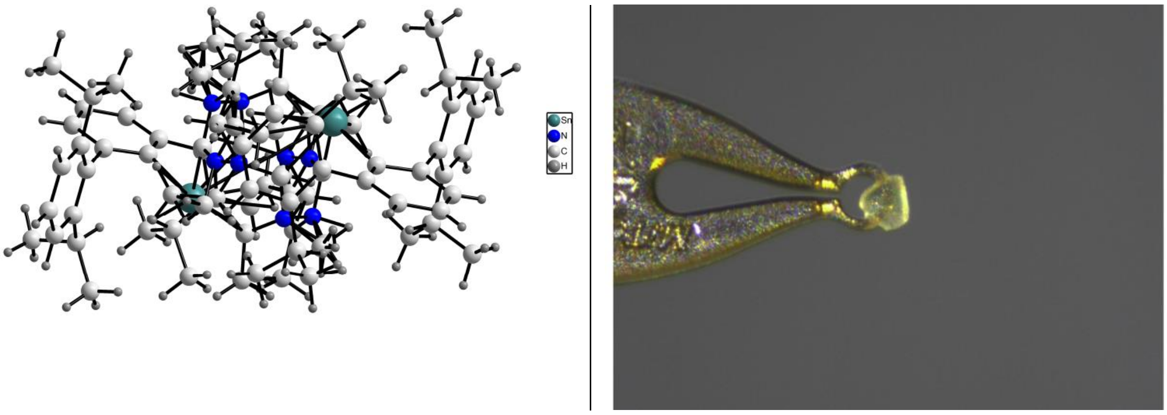

Figure S42. Left: Asymmetric unit of **4b** (polymorph b); Right: Picture of the crystal used for data collection.

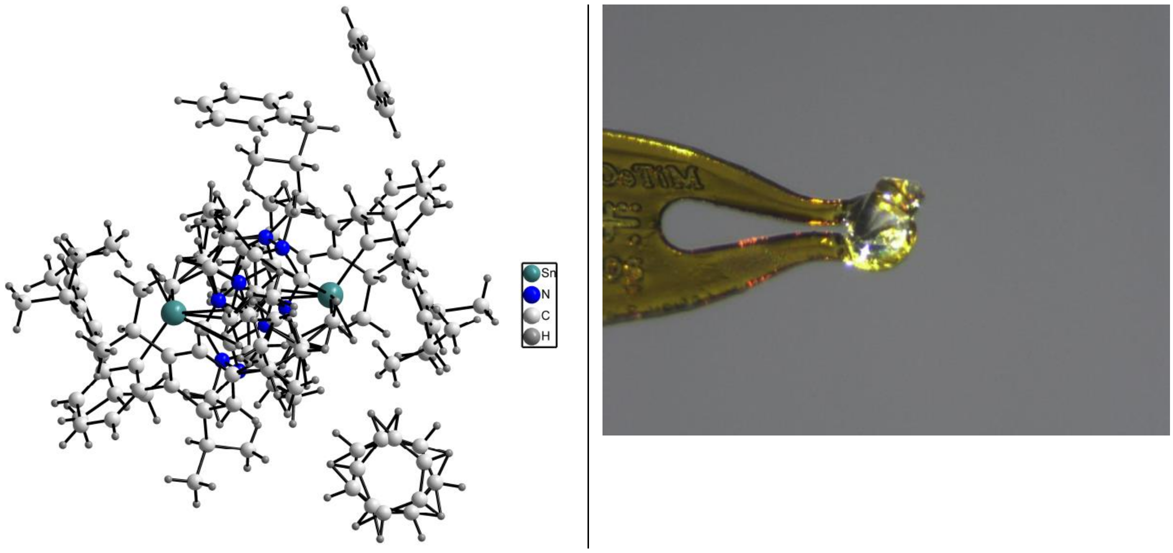

Figure S43. Left: Asymmetric unit of **4c**; Right: Picture of the crystal used for data collection.

5a: Refined as a two-component twin.

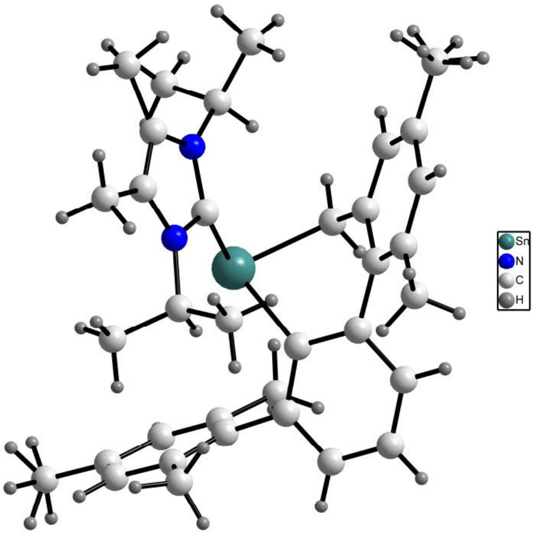

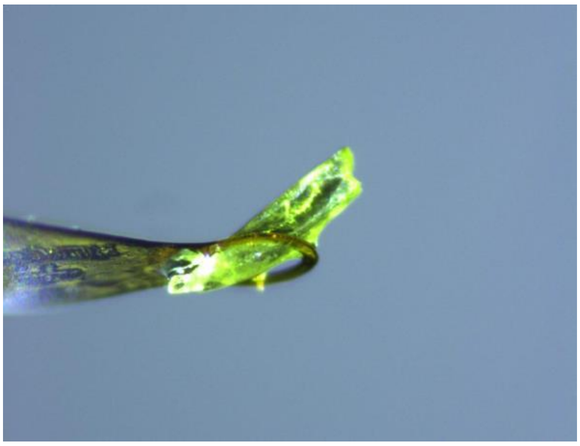

Figure S44. Left: Asymmetric unit of **5a**; Right: Picture of the crystal used for data collection.

6b: Block-shaped crystals: The diffraction pattern displays diffuse reflections, suggesting potential twinning resulting from crystal fragmentation. However, due to significant overlap, no distinct twin domains could be indexed. While one twin-law (0.988 -0.001 0.024 / 0.249 -1 0.003 / 0.994 0 -0.988) was identified, it did not lead to a significant improvement in data quality.

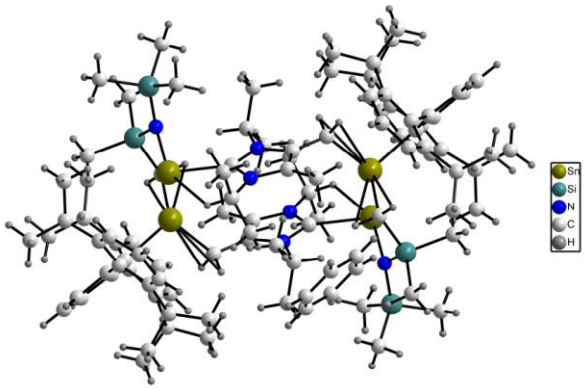

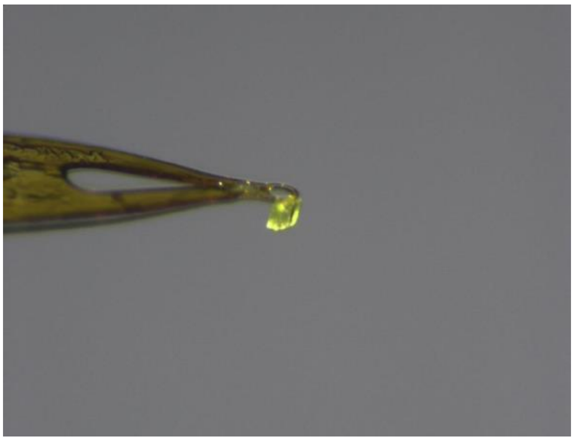

Figure S45. Left: Asymmetric unit of **6b** (block-shaped crystals); Right: Picture of the crystal used for data collection.

6b: Needle-shaped crystals: Similar to the other polymorph, the diffraction pattern is not perfectly clean but presents fewer challenges during data integration. The residual density artifacts observed here resemble typical absorption effects caused by the tin.

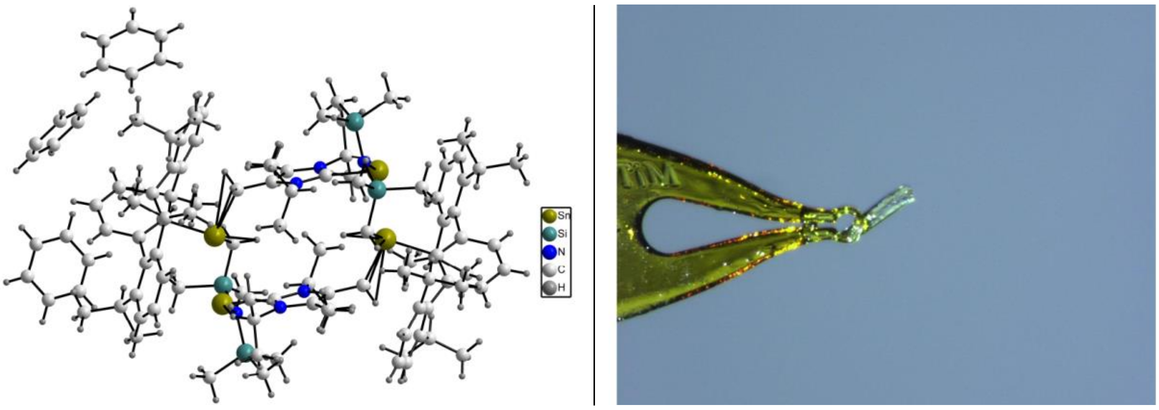

Figure S46. Left: Asymmetric unit of **6b** (needle-shaped crystals); Right: Picture of the crystal used for data collection.

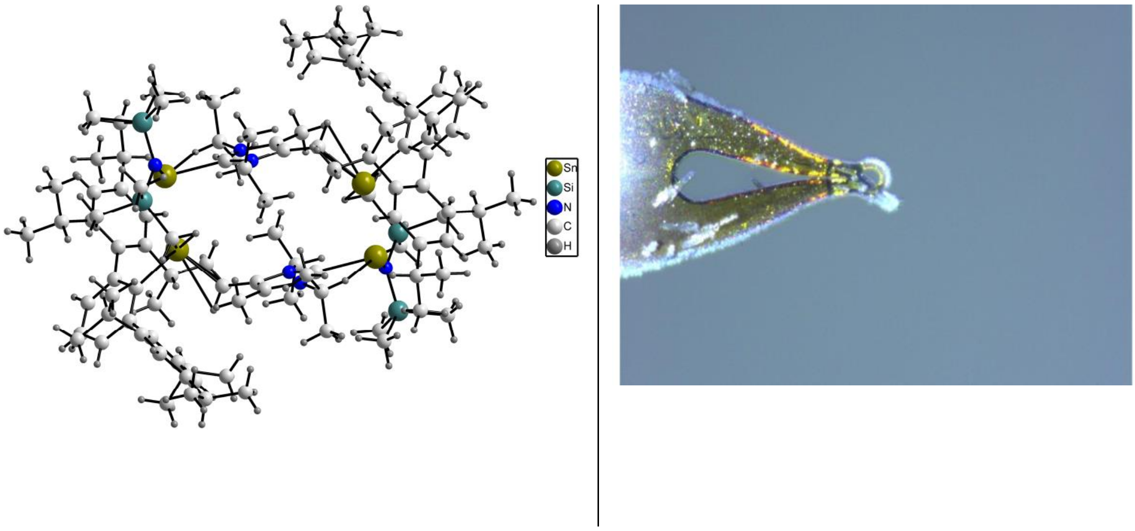

Figure S47. Left: Asymmetric unit of **6c**; Right: Picture of the crystal used for data collection.

Table S1. Crystal structure data for compounds **2a**, **3a** and **4b** (polymorph a).

	**2a**	**3a**	**4b** (polymorph a)
CCDC number	2386607	2386608	2386609
Empirical formula	$C_{37}H_{55}N_3Si_2Sn$	$C_{140.32}H_{160.32}N_8Sn_4$	$C_{116}H_{138}N_4Sn_2$
Formula weight	716.71	2433.61	1825.68
Temperature [K]	100.00	100.00	100.00
Crystal system	monoclinic	triclinic	triclinic
Space group (number)	$P2_1/n$ (14)	$P\bar{1}$ (2)	$P\bar{1}$ (2)
a [Å]	10.5872(3)	17.3865(7)	11.8124(5)
b [Å]	21.0768(12)	18.9013(9)	12.0378(5)
c [Å]	16.8089(7)	19.8150(8)	17.8539(8)
α [°]	90	88.498(2)	96.1960(10)
β [°]	100.538(2)	88.718(2)	101.9410(10)
γ [°]	90	67.8100(10)	95.6870(10)
Volume [$Å^3$]	3687.5(3)	6026.9(4)	2449.98(18)
Z	4	2	1
ρ_{calc} [gcm^{-3}]	1.291	1.341	1.237
μ [mm^{-1}]	0.786	0.873	0.560
F(000)	1504	2516	962
Crystal size [mm^3]	0.262×0.175×0.119	0.345×0.319×0.149	0.144×0.117×0.11
Crystal colour	yellow	colourless	colourless
Crystal shape	block	block	block
Radiation	MoK_α (λ=0.71073 Å)	MoK_α (λ=0.71073 Å)	MoK_α (λ=0.71073 Å)
2θ range [°]	4.23 to 65.17 (0.66 Å)	4.03 to 59.15 (0.72 Å)	3.89 to 65.16 (0.66 Å)
Index ranges	$-13 \le h \le 16$<br>$-31 \le k \le 31$<br>$-25 \le l \le 22$	$-24 \le h \le 23$<br>$-26 \le k \le 26$<br>$-27 \le l \le 27$	$-17 \le h \le 15$<br>$-18 \le k \le 18$<br>$-27 \le l \le 27$
Reflections collected	140490	322409	154899
Independent reflections	13414<br>R_{int} = 0.0255<br>R_{sigma} = 0.0127	33785<br>R_{int} = 0.0212<br>R_{sigma} = 0.0101	17774<br>R_{int} = 0.0257<br>R_{sigma} = 0.0137
Completeness to θ = 25.242°	100.0 %	100.0 %	99.9 %
Data / Restraints / Parameters	13414/0/409	33785/525/1530	17774/0/561
Absorption correction T_{min}/T_{max} (method)	0.8548/0.9439 (numerical)	0.7915/0.9221 (multi-scan)	0.9234/0.9713 (numerical)
Goodness-of-fit on F^2	1.058	1.074	1.085
Final *R* indexes [$I \geq 2\sigma(I)$]	R_1 = 0.0187<br>wR_2 = 0.0474	R_1 = 0.0201<br>wR_2 = 0.0492	R_1 = 0.0236<br>wR_2 = 0.0598
Final *R* indexes [all data]	R_1 = 0.0217<br>wR_2 = 0.0493	R_1 = 0.0227<br>wR_2 = 0.0513	R_1 = 0.0254<br>wR_2 = 0.0610
Largest peak/hole [$eÅ^{-3}$]	0.52/−0.32	0.63/−0.91	1.24/−0.67

Table S2. Crystal structure data for compounds **4b** (polymorph b), **4c** and **5a**.

	**4b** (polymorph b)	**4c**	**5a**
CCDC number	2411687	2386610	2386611
Empirical formula	$C_{74}H_{96}N_4Sn_2$	$C_{116}H_{150}N_4Sn_2$	$C_{35}H_{44}N_2Sn$
Formula weight	1278.92	1837.77	611.41
Temperature [K]	100.00	100.00	100.00
Crystal system	monoclinic	triclinic	triclinic
Space group (number)	$C2/c$ (15)	$P\bar{1}$ (2)	$P\bar{1}$ (2)
a [Å]	32.775(3)	12.9385(10)	9.8966(7)
b [Å]	11.3293(9)	14.0610(9)	12.7337(7)
c [Å]	17.5417(14)	16.5869(14)	13.8474(8)
α [°]	90	106.832(2)	69.103(2)
β [°]	92.413(4)	99.262(2)	71.796(2)
γ [°]	90	112.283(2)	85.167(2)
Volume [$Å^3$]	6507.8(10)	2544.7(3)	1547.95(17)
Z	4	1	2
ρ_{calc} [gcm^{-3}]	1.305	1.199	1.312
μ [mm^{-1}]	0.812	0.539	0.850
F(000)	2672	974	636
Crystal size [mm^3]	0.082×0.152×0.187	0.332×0.199×0.193	0.838×0.205×0.166
Crystal colour	yellow	yellow	yellow
Crystal shape	block	block	block
Radiation	MoK_α (λ=0.71073 Å)	MoK_α (λ=0.71073 Å)	MoK_α (λ=0.71073 Å)
2θ range [°]	4.43 to 63.22 (0.68 Å)	4.41 to 65.39 (0.66 Å)	4.33 to 63.01 (0.68 Å)
Index ranges	−48 ≤ h ≤ 48<br>−16 ≤ k ≤ 14<br>−25 ≤ l ≤ 24	−19 ≤ h ≤ 19<br>−21 ≤ k ≤ 21<br>−25 ≤ l ≤ 25	−14 ≤ h ≤ 14<br>−18 ≤ k ≤ 18<br>−20 ≤ l ≤ 20
Reflections collected	100789	162025	16011
Independent reflections	10915<br>R_{int} = 0.0555<br>R_{sigma} = 0.0282	18668<br>R_{int} = 0.0449<br>R_{sigma} = 0.0221	16011<br>R_{int} = 0.0249<br>R_{sigma} = 0.0215
Completeness to θ = 25.242°	100.0 %	99.9 %	66.7 %
Data / Restraints / Parameters	10915 / 16 / 400	18668/90/725	16011/0/357
Absorption correction T_{min}/T_{max} (method)	0.5710 / 0.7462 (numerical)	0.7541/1.0000 (numerical)	0.252836/0.318214 (multi-scan)
Goodness-of-fit on F^2	1.040	1.119	1.061
Final *R* indexes [I≥2σ(I)]	R_1 = 0.0271<br>wR_2 = 0.0588	R_1 = 0.0301<br>wR_2 = 0.0690	R_1 = 0.0211<br>wR_2 = 0.0610
Final *R* indexes [all data]	R_1 = 0.0356<br>wR_2 = 0.0631	R_1 = 0.0329<br>wR_2 = 0.0704	R_1 = 0.0218<br>wR_2 = 0.0615
Largest peak/hole [$eÅ^{-3}$]	0.43/−0.32	1.73/−1.47	0.93/−0.46

Table S3. Crystal structure data for compounds **6b** (polymorph b) and **6c**.

	**6b** (polymorph a)	**6b** (polymorph b)	**6c**
CCDC number	2386613	2386612	2386614
Empirical formula	$C_{124}H_{174}N_6Si_4Sn_4$	$C_{106}H_{158}N_6Si_4Sn_4$	$C_{106}H_{170}N_6Si_4Sn_4$
Formula weight	2335.80	2103.49	2115.59
Temperature [K]	100.00	100.00	100.00
Crystal system	triclinic	triclinic	triclinic
Space group (number)	$P\bar{1}$ (2)	$P\bar{1}$ (2)	$P\bar{1}$ (2)
a [Å]	11.886(3)	12.505(3)	11.8302(19)
b [Å]	12.567(3)	14.068(6)	14.801(3)
c [Å]	20.980(5)	16.593(6)	17.083(4)
α [°]	78.253(7)	98.266(7)	112.561(8)
β [°]	74.700(7)	103.799(12)	100.184(8)
γ [°]	83.390(7)	109.427(13)	92.374(9)
Volume [$Å^3$]	2953.2(13)	2592.8(16)	2699.0(9)
Z	1	1	1
ρ_{calc} [gcm^{-3}]	1.313	1.347	1.302
μ [mm^{-1}]	0.926	1.046	1.005
F(000)	1216	1092	1104
Crystal size [mm^3]	0.139×0.099×0.071	0.371×0.064×0.048	0.219×0.06×0.042
Crystal colour	yellow	yellow	yellow
Crystal shape	plate	needle	needle
Radiation	MoK_α (λ=0.71073 Å)	MoK_α (λ=0.71073 Å)	MoK_α (λ=0.71073 Å)
2θ range [°]	4.09 to 56.07 (0.76 Å)	3.63 to 54.51 (0.78 Å)	3.94 to 58.30 (0.73 Å)
Index ranges	−15 ≤ h ≤ 15<br>−16 ≤ k ≤ 16<br>−27 ≤ l ≤ 27	−15 ≤ h ≤ 15<br>−18 ≤ k ≤ 18<br>−21 ≤ l ≤ 21	−15 ≤ h ≤ 16<br>−20 ≤ k ≤ 20<br>−23 ≤ l ≤ 23
Reflections collected	94926	79486	130978
Independent reflections	14168<br>R_{int} = 0.0792<br>R_{sigma} = 0.0575	11392<br>R_{int} = 0.0707<br>R_{sigma} = 0.0466	14559<br>R_{int} = 0.0667<br>R_{sigma} = 0.0408
Completeness to θ = 25.242°	99.9 %	99.9 %	100.0 %
Data / Restraints / Parameters	14168/433/640	11392/0/559	14559/0/563
Absorption correction T_{min}/T_{max} (method)	0.7086/1.0000 (numerical)	0.7095/1.0000 (numerical)	0.8910/0.9895 (numerical)
Goodness-of-fit on F^2	1.120	1.042	1.068
Final *R* indexes [$I \geq 2\sigma(I)$]	R_1 = 0.0698<br>wR_2 = 0.1598	R_1 = 0.0458<br>wR_2 = 0.1134	R_1 = 0.0416<br>wR_2 = 0.0732
Final *R* indexes [all data]	R_1 = 0.0847<br>wR_2 = 0.1657	R_1 = 0.0580<br>wR_2 = 0.1231	R_1 = 0.0625<br>wR_2 = 0.0808
Largest peak/hole [$eÅ^{-3}$]	4.12/−4.28	2.19/−1.06	1.73/−1.06

Computational Details

Geometry optimizations, frequency calculations and PCM solvent corrections were run with Gaussian 16 Revision A.03[12] using the BP86[13,14] functional. For geometry optimisations, all atoms were described with def2-SVP basis sets of Ahlrichs and Weigand.[15] Single point energy calculations were performed on the optimised geometries, at the BP86/def2-TZVP level of theory. Stationary points were fully characterized using analytical frequency calculations as either minima (all positive eigenvalues) or transition states (one negative eigenvalue). IRC calculations and subsequent geometry optimizations were used to confirm the minima linked by the transition states. Energies reported in the text are based on the gas-phase free energies and incorporate a correction for dispersion effects using Grimme's D3 parameter set with Becke-Johnson dampening[16,17] (i.e. BP86-D3BJ) as well as solvation (PCM approach) in benzene. Energies are given in atomic units (a.u.) unless otherwise stated.

Natural Bond Orbital (NBO) and Natural Localised Molecular Orbital (NLMO) analysis was performed using NBO-7 using single point calculations performed at the BP86/def2-SVP or BP86/def2-TZVP level of theory.[18]

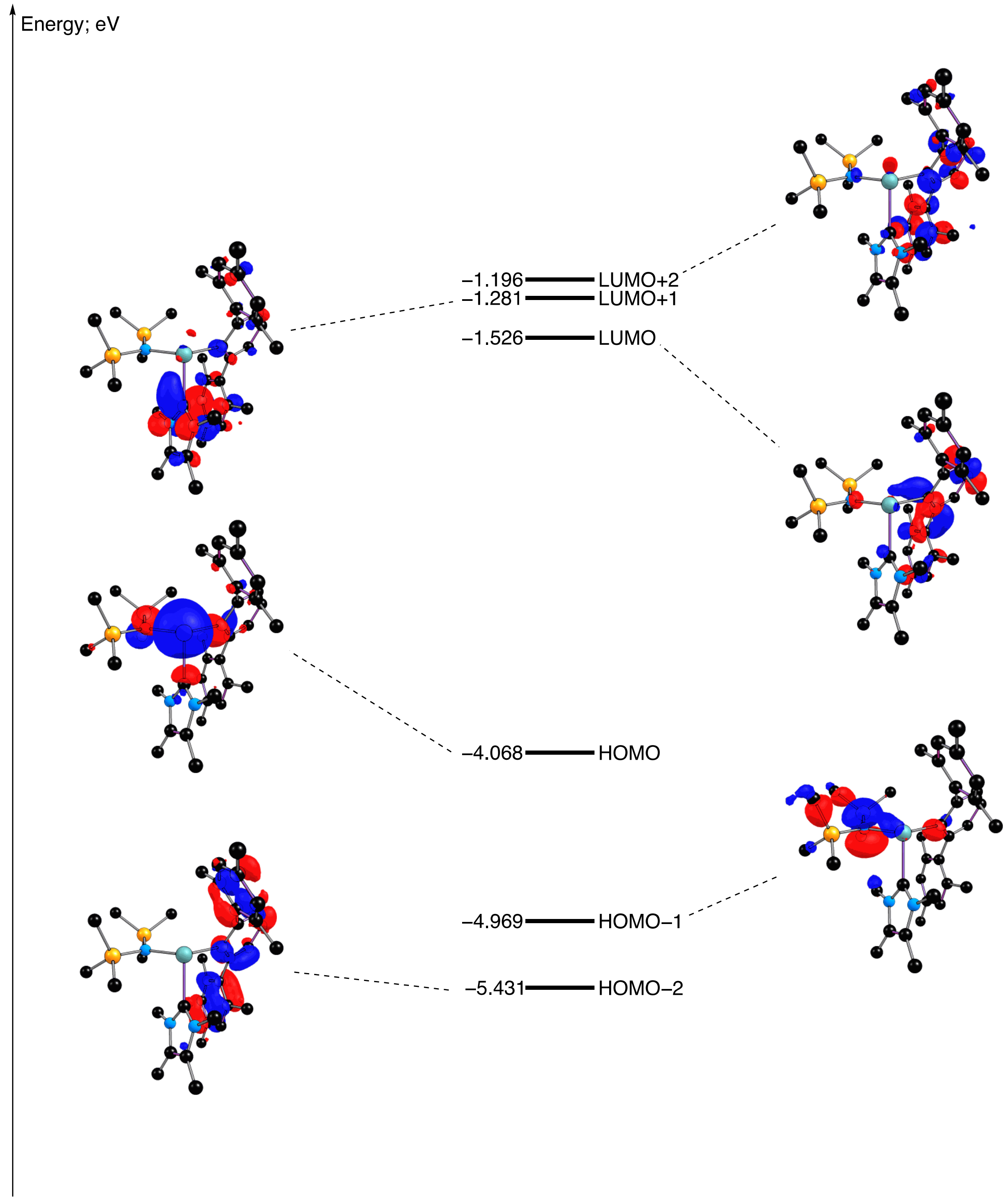

Figure S48. Kohn-Sham Molecular Orbital (BP86/def2-TZVP) diagram of **2a**.

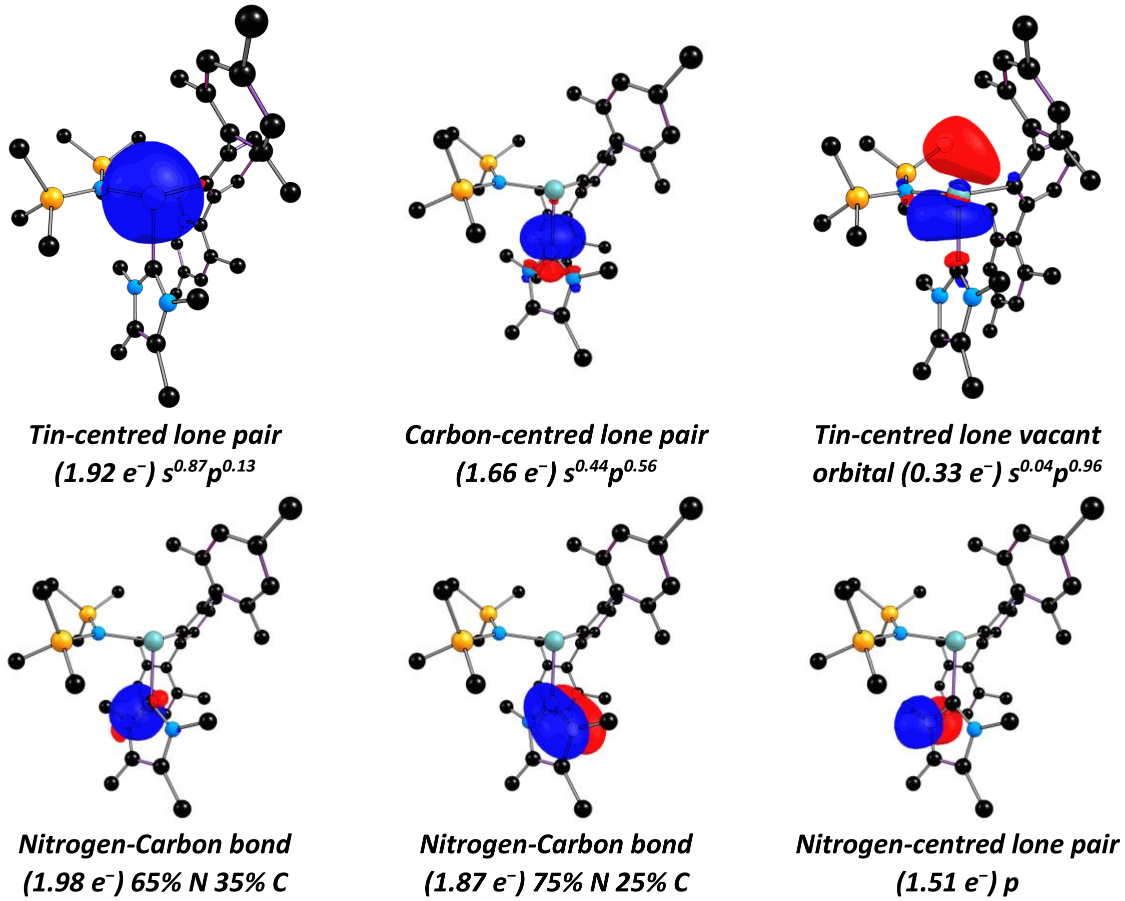

Figure S49. Selected Natural Bond Orbitals (BP86/def2-TZVP) diagram of **2a**.

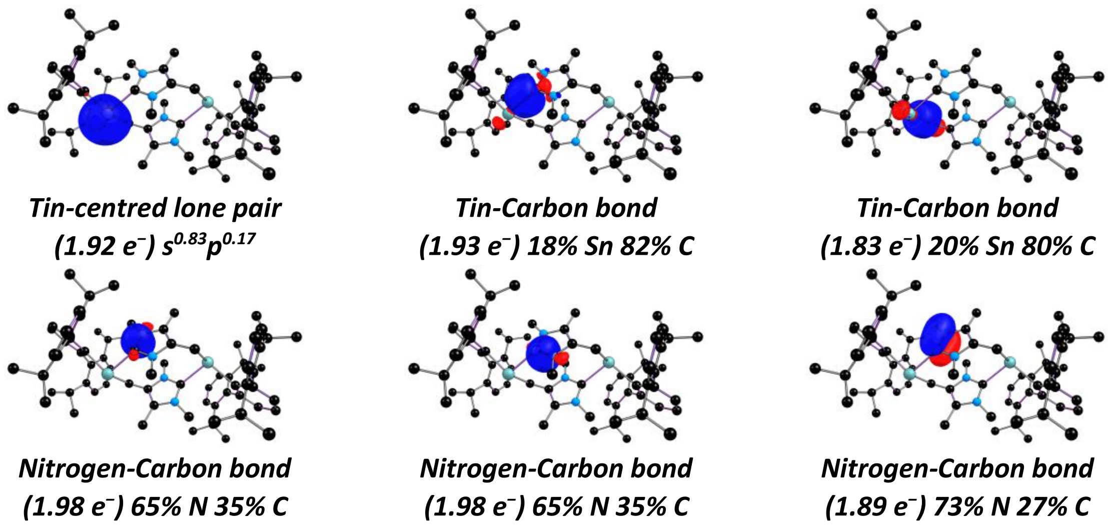

Figure S50. Selected Natural Bond Orbitals (BP86/def2-TZVP) diagram of **4b**.

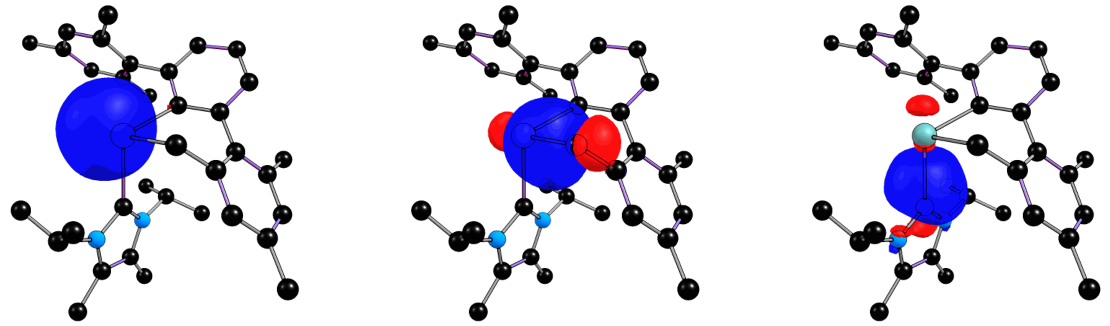

Tin-centred lone pair
(1.92 e^-) $s^{0.83}p^{0.17}$

Tin-Carbon bond
(1.86 e^-) 24% Sn 76% C

Tin-Carbon bond
(1.93 e^-) 18% Sn 81% C

Figure S51. Selected Natural Bond Orbitals (BP86/def2-TZVP) diagram of **5a**.

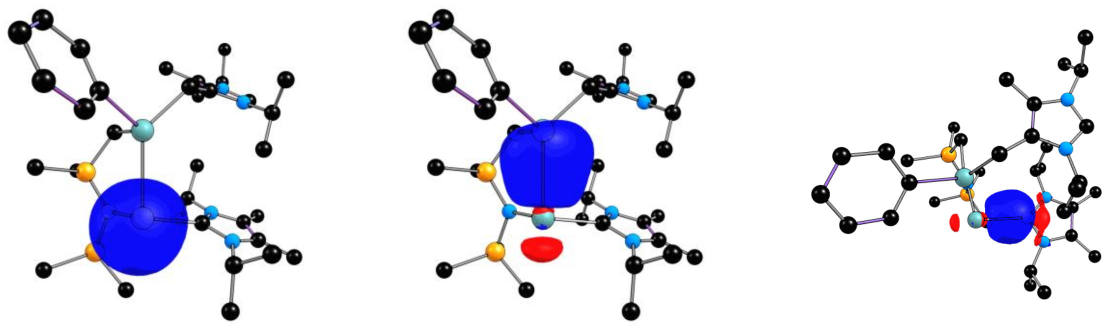

Tin-centred lone pair
(1.93 e^-) $s^{0.89}p^{0.11}$

Tin-Tin bond
(1.85 e^-) 64% Sn 36% Sn

Tin-Carbon bond
(1.95 e^-) 18% Sn 82% C

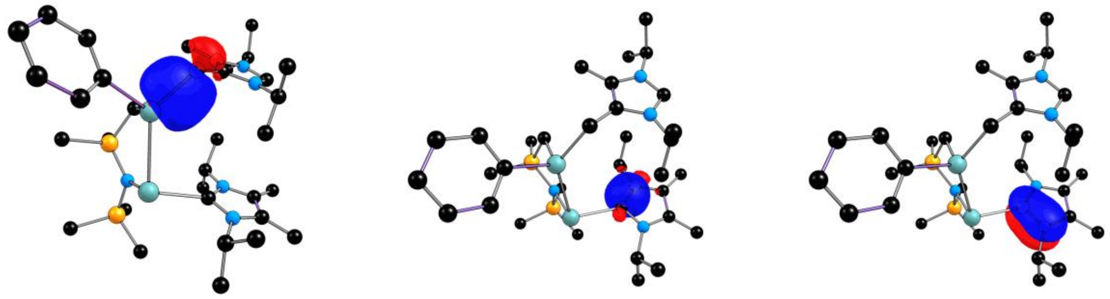

Tin-Carbon bond
(1.88 e^-) 26% Sn 74% C

Nitrogen-Carbon bond
(1.98 e^-) 66% N 34% C

Nitrogen-Carbon bond
(1.89 e^-) 73% N 27% C

Figure S52. Selected Natural Bond Orbitals (BP86/def2-TZVP) diagram of **6b**.

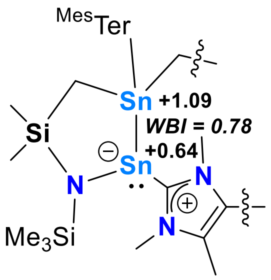

Figure S53. Schematic of the bonding situation in the core of **6b**, charges shown are derived from Natural Population Analysis (NPA), and WBI = Wiberg Bond Index.

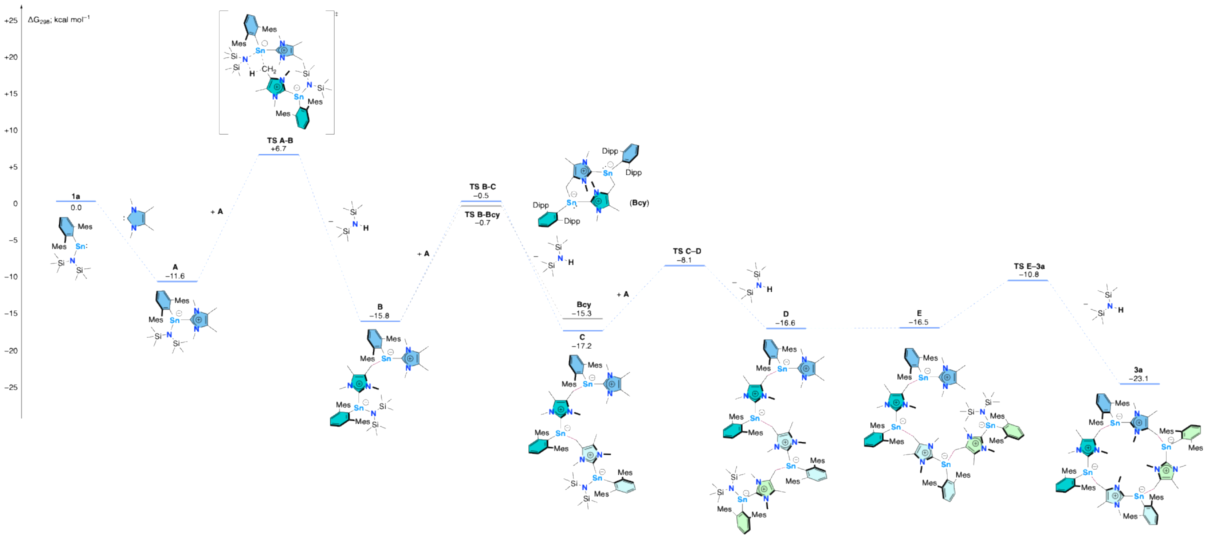

Figure S54. Calculated Mechanism (BP86-D3BJ/def2-TZVP/PCM=Benzene) for the formation of **3a**.

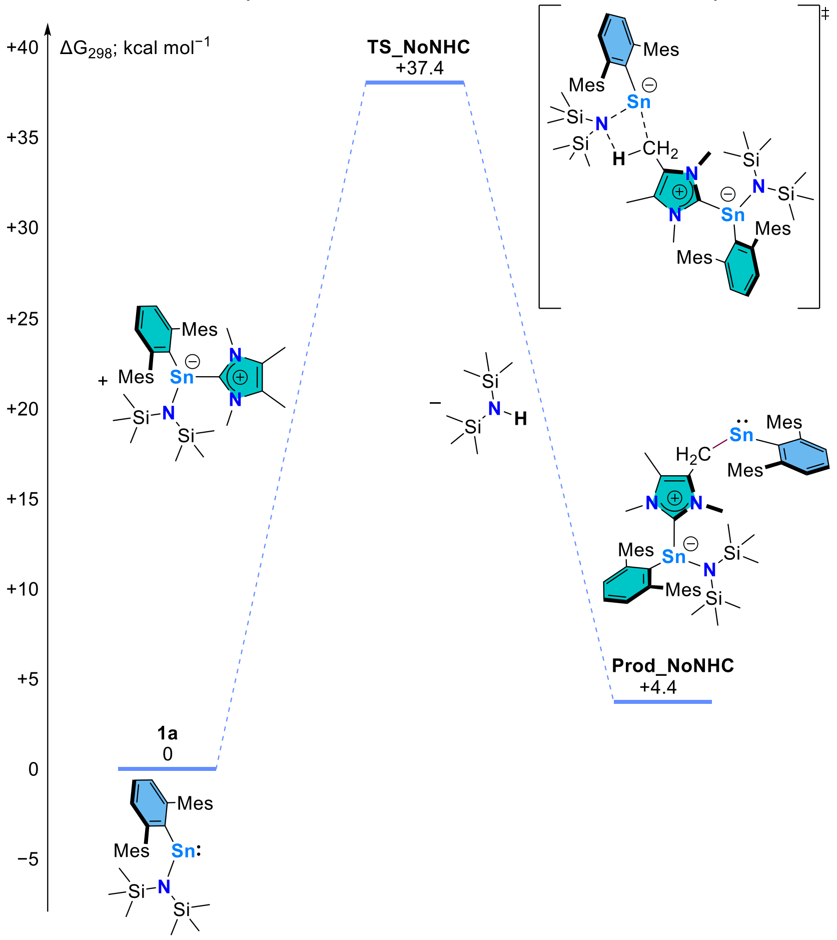

Figure S55. Calculated Mechanism (BP86-D3BJ/Def2-TZVP/PCM=Benzene) for the hypothetical reaction of **1a** and **2a**.

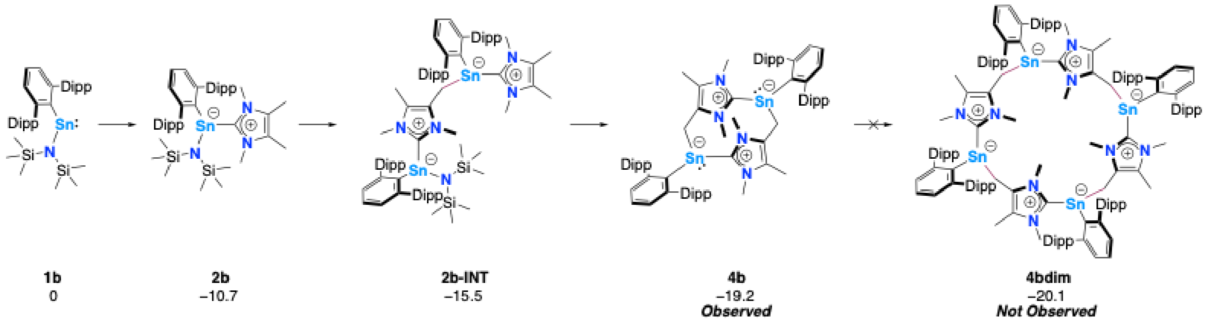

Figure S56. Calculated Mechanism and Energetic Comparison of Products (BP86-D3BJ/def2-TZVP/PCM=Benzene) in the formation of **4b**.

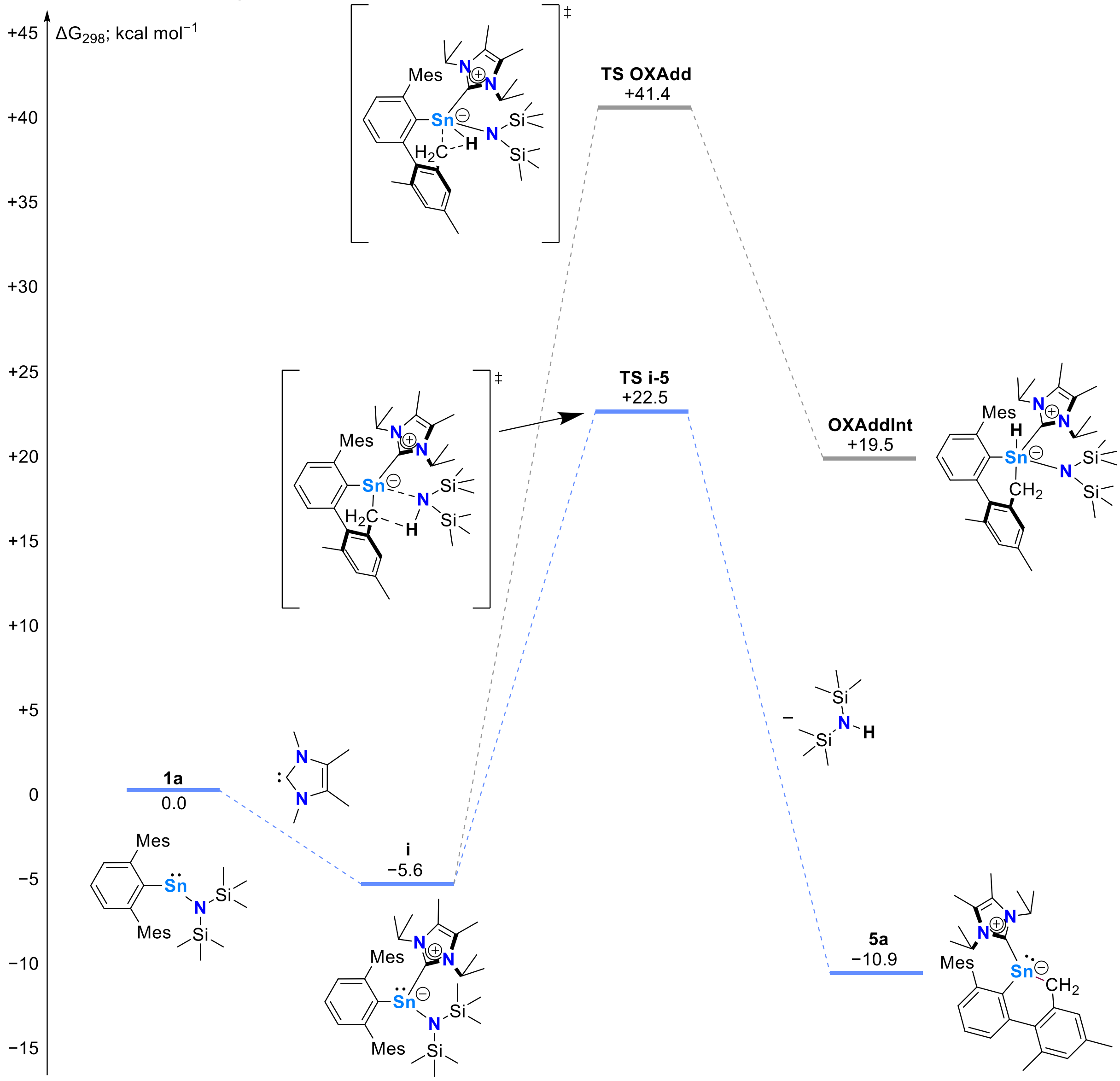

Figure S57. Calculated Mechanism (BP86-D3BJ/Def2-TZVP/PCM=Benzene) for the formation of **5a**. The investigated, but energetically unfeasible oxidative addition pathway is shown in grey.

Energies and Frequencies for the DFT Optimised Geometries

1a
SCF = -2016.34775970
H(0 K) = -2015.724718
H(298 K) = -2015.678988
G(298 K) = -2015.805284
SCF+D3 = -2016.55620561
PCM SCF (Benzene) = -2016.34984705
BS2 (def2-tzvp) = -2017.87641272
Low Freq. = 11.5799cm-1, 19.7113cm-1

1b
SCF = -2252.02008847
H(0 K) = -2251.229347
H(298 K) = -2251.177013
G(298 K) = -2251.314643
SCF+D3 = -2252.27714034
PCM SCF (Benzene) = -2252.02216159
BS2 (def2-tzvp) = -2253.79958272
Low Freq. = 13.5333cm-1, 18.8090cm-1

HHMDS
SCF = -873.580513276
H(0 K) = -873.350248
H(298 K) = -873.332018
G(298 K) = -873.394200
SCF+D3 = -873.627297014
PCM SCF (Benzene) = -873.581289692
BS2 (def2-tzvp) = -874.125928025
Low Freq. = 24.9284cm-1, 32.6687cm-1

IMe4
SCF = -383.154110374
H(0 K) = -382.977933
H(298 K) = -382.966601
G(298 K) = -383.012744
SCF+D3 = -383.184508999
PCM SCF (Benzene) = -383.158238925
BS2 (def2-tzvp) = -383.569156659
Low Freq. = 122.4971cm-1, 123.4304cm-1

IiPr2Me2
SCF = -540.289452294
H(0 K) = -540.004074
H(298 K) = -539.987220
G(298 K) = -540.047345
SCF+D3 = -540.342884476
PCM SCF (Benzene) = -540.292691205
BS2 (def2-tzvp) = -540.871699162
Low Freq. = 16.6897cm-1, 32.5431cm-1

DippTerH
SCF = -1165.23954952
H(0 K) = -1164.660210
H(298 K) = -1164.626664
G(298 K) = -1164.728794
SCF+D3 = -1165.38686167
PCM SCF (Benzene) = -1165.24151010
BS2 (def2-tzvp) = -1166.47321928
Low Freq. = 6.8066cm-1, 16.2612cm-1

2a
SCF = -2399.52445893
H(0 K) = -2398.722863
H(298 K) = -2398.665486
G(298 K) = -2398.815823
SCF+D3 = -2399.79086453
PCM SCF (Benzene) = -2399.52934659
BS2 (def2-tzvp) = -2401.46255367
Low Freq. = 14.4829cm-1, 21.0727cm-1

TS2a-B
SCF = -4798.98254110
H(0 K) = -4797.384722
H(298 K) = -4797.268901
G(298 K) = -4797.550505
SCF+D3 = -4799.54173968
PCM SCF (Benzene) = -4798.99433177
BS2 (def2-tzvp) = -4802.85343459
Low Freq. = -429.9729cm-1, 6.8918cm-1

B
SCF = -3925.48098193
H(0 K) = -3924.109661
H(298 K) = -3924.012158
G(298 K) = -3924.255249
SCF+D3 = -3925.96143917
PCM SCF (Benzene) = -3925.48990332
BS2 (def2-tzvp) = -3928.81297205
Low Freq. = 4.3732cm-1, 8.2793cm-1

TSB-BCy
SCF = -3925.42760048
H(0 K) = -3924.061239
H(298 K) = -3923.964574
G(298 K) = -3924.202994
SCF+D3 = -3925.92005840
PCM SCF (Benzene) = -3925.43379894
BS2 (def2-tzvp) = -3928.75453845
Low Freq. = -310.5045cm-1, 6.8505cm-1

BCy
SCF = -3051.90727309
H(0 K) = -3050.767711
H(298 K) = -3050.689621
G(298 K) = -3050.887528
SCF+D3 = -3052.31261801
PCM SCF (Benzene) = -3051.91363775
BS2 (def2-tzvp) = -3054.69603699
Low Freq. = 10.8147cm-1, 11.3448cm-1

TSB-C
SCF = -6324.93249539
H(0 K) = -6322.764154
H(298 K) = -6322.608513
G(298 K) = -6322.981948
SCF+D3 = -6325.70646748
PCM SCF (Benzene) = -6324.94813970
BS2 (def2-tzvp) = -6330.19670825
Low Freq. = -247.5797cm-1, 2.8639cm-1

C
SCF = -5451.43965356
H(0 K) = -5449.498524
H(298 K) = -5449.361012
G(298 K) = -5449.694383
SCF+D3 = -5452.13542895
PCM SCF (Benzene) = -5451.45219495
BS2 (def2-tzvp) = -5456.16509100
Low Freq. = 5.3788cm-1, 5.8228cm-1

TSC-D
SCF = -7850.90198107
H(0 K) = -7848.162188
H(298 K) = -7847.966870
G(298 K) = -7848.427118
SCF+D3 = -7851.90626646
PCM SCF (Benzene) = -7850.91923229
BS2 (def2-tzvp) = -7857.55798179
Low Freq. = -65.1894cm-1, 3.8986cm-1

D
SCF = -6977.39180654
H(0 K) = -6974.881355
H(298 K) = -6974.703615
G(298 K) = -6975.129321
SCF+D3 = -6978.30218736
PCM SCF (Benzene) = -6977.40539546
BS2 (def2-tzvp) = -6983.50986811
Low Freq. = 4.4954cm-1, 5.2495cm-1

E
SCF = -6977.39183329
H(0 K) = -6974.882232
H(298 K) = -6974.703895
G(298 K) = -6975.131895
SCF+D3 = -6978.29681891
PCM SCF (Benzene) = -6977.40571684
BS2 (def2-tzvp) = -6983.51155113
Low Freq. = 3.6380cm-1, 4.8154cm-1

TSE-3a
SCF = -6977.33643941
H(0 K) = -6974.831961
H(298 K) = -6974.654748
G(298 K) = -6975.078227
SCF+D3 = -6978.26900303
PCM SCF (Benzene) = -6977.34719461
BS2 (def2-tzvp) = -6983.44921857
Low Freq. = -573.9213cm-1, 4.6473cm-1

3a
SCF = -6103.84124229
H(0 K) = -6101.562493
H(298 K) = -6101.403754
G(298 K) = -6101.785440
SCF+D3 = -6104.69650566
PCM SCF (Benzene) = -6103.85182347
BS2 (def2-tzvp) = -6109.41570934
Low Freq. = 6.0016cm-1, 7.9146cm-1

i
SCF = -2556.64559373
H(0 K) = -2555.734395
H(298 K) = -2555.671755
G(298 K) = -2555.831944
SCF+D3 = -2556.94351555
PCM SCF (Benzene) = -2556.64995361
BS2 (def2-tzvp) = -2558.75097720
Low Freq. = 17.9146cm-1, 20.5815cm-1

TSi-5a
SCF = -2556.59688300
H(0 K) = -2555.691881
H(298 K) = -2555.629684
G(298 K) = -2555.790064
SCF+D3 = -2556.89513161
PCM SCF (Benzene) = -2556.60111869
BS2 (def2-tzvp) = -2558.69916106
Low Freq. = -707.1543cm-1, 9.3275cm-1

5a
SCF = -1683.08445926
H(0 K) = -1682.405466
H(298 K) = -1682.361704
G(298 K) = -1682.482895
SCF+D3 = -1683.29325742
PCM SCF (Benzene) = -1683.08983450
BS2 (def2-tzvp) = -1684.64836951
Low Freq. = 14.5655cm-1, 17.5096cm-1

2b
SCF = -2635.19166683
H(0 K) = -2634.222603
H(298 K) = -2634.158553
G(298 K) = -2634.319683
SCF+D3 = -2635.51123251
PCM SCF (Benzene) = -2635.19618951
BS2 (def2-tzvp) = -2637.38045966
Low Freq. = 9.2467cm-1, 20.0342cm-1

2b-INT
SCF = -4396.81825481
H(0 K) = -4395.112098
H(298 K) = -4395.001229
G(298 K) = -4395.265789
SCF+D3 = -4397.40428004
PCM SCF (Benzene) = -4396.82640800
BS2 (def2-tzvp) = -4400.65163791
Low Freq. = 5.5228cm-1, 10.0197cm-1

4b
SCF = -3523.25324332
H(0 K) = -3521.779937
H(298 K) = -3521.687899
G(298 K) = -3521.911985
SCF+D3 = -3523.76323646
PCM SCF (Benzene) = -3523.25897785
BS2 (def2-tzvp) = -3526.54337397
Low Freq. = 8.0986cm-1, 13.7450cm-1

4bdim
SCF = -7046.49603687
H(0 K) = -7043.546699
H(298 K) = -7043.361768
G(298 K) = -7043.784966
SCF+D3 = -7047.56798503
PCM SCF (Benzene) = -7046.50488281
BS2 (def2-tzvp) = -7053.07211334
Low Freq. = 6.3298cm-1, 7.2772cm-1

TS1b-INTIiPr2Me2
SCF = -2792.30421469
H(0 K) = -2791.225999
H(298 K) = -2791.157255
G(298 K) = -2791.328709
SCF+D3 = -2792.64891356
PCM SCF (Benzene) = -2792.30794206
BS2 (def2-tzvp) = -2794.66077724
Low Freq. = -15.1031cm-1, 7.8674cm-1

INTiPr2Me2
SCF = -2792.30600201
H(0 K) = -2791.227120
H(298 K) = -2791.157818
G(298 K) = -2791.329019
SCF+D3 = -2792.65612059
PCM SCF (Benzene) = -2792.31005893
BS2 (def2-tzvp) = -2794.66176991
Low Freq. = 15.6069cm-1, 19.4789cm-1

6b
SCF = -6011.14825327
H(0 K) = -6009.031194
H(298 K) = -6008.890895
G(298 K) = -6009.212736
SCF+D3 = -6011.93712279
PCM SCF (Benzene) = -6011.15361034
BS2 (def2-tzvp) = -6015.86387282
Low Freq. = 10.7720cm-1, 13.5236cm-1

TSOXAdd
SCF = -2556.55732238
H(0 K) = -2555.651812
H(298 K) = -2555.590166
G(298 K) = -2555.744881
SCF+D3 = -2556.86328301
PCM SCF (Benzene) = -2556.56431645
BS2 (def2-tzvp) = -2558.66419010
Low Freq. = -434.9918cm-1, 21.6317cm-1

OXAddInt
SCF = -2556.60033877
H(0 K) = -2555.692484
H(298 K) = -2555.630716
G(298 K) = -2555.786785
SCF+D3 = -2556.90345520
PCM SCF (Benzene) = -2556.60444342
BS2 (def2-tzvp) = -2558.70594779
Low Freq. = 15.5053cm-1, 21.5919cm-1

TS_NoNHC
SCF = -4415.80576070
H(0 K) = -4414.385578
H(298 K) = -4414.281793
G(298 K) = -4414.539986
SCF+D3 = -4416.30911646
PCM SCF (Benzene) = -4415.81250856
BS2 (def2-tzvp) = -4419.26581697
Low Freq. = -256.0225cm-1, 2.9885cm-1

Prod_NoNHC
SCF = -3542.28683085
H(0 K) = -3541.094727
H(298 K) = -3541.008620
G(298 K) = -3541.230824
SCF+D3 = -3542.70256335
PCM SCF (Benzene) = -3542.29320065
BS2 (def2-tzvp) = -3545.20941960
Low Freq. = 4.7886cm-1, 7.6703cm-1

References

1 Barnett, C., Cole, M. L. & Harper, J. B. Steric Properties of *N*-Heterocyclic Carbenes affect the Performance of Electronic Probes. *Eur. J. Inorg. Chem.*, 4954-4958 (2021).

2 Kuhn, N. & Kratz, T. Synthesis of Imidazol-2-ylidenes by Reduction of Imidazole-2(3*H*)-thiones. *Synthesis*, 561-562 (1993).

3 Fischer, M., Roy, M. M. D, Wales, L. L., Ellwanger, M. A., Heilmann, A. & Aldridge, S. Structural Snapshots in Reversible Phosphinidene Transfer. *J. Am. Chem. Soc.* **144**, 8908-8913 (2022).

4 Pu, L., Olmstead, M. M., Power, P. P. & Schiemenz, B. Synthesis and Characterization of the Monomeric Terphenyl–Metal Halides Ge(Cl){C_6H_3-2,6-$Trip_2$} (Trip = C_6H_2-2,4,6-*i*-Pr_3) and Sn(I){C_6H_3-2,6-$Trip_2$} and the Terphenyl–Metal Amide Sn{N($SiMe_3$)$_2$}{C_6H_3-2,6-$Trip_2$}. *Organometallics* **17**, 5602-5606 (1998).

5 Bruker, *SAINT*, *V8.40B*, Bruker AXS Inc., Madison, Wisconsin, USA.

6 Krause, L., Herbst-Irmer, R., Sheldrick, G. M. & Stalke, D. Comparison of silver and molybdenum microfocus X-ray sources for single-crystal structure determination. *J. Appl. Cryst.* **48**, 3-10 (2015).

7 Sheldrick, G. M. *SHELXT* – Integrated space-group and crystal-structure determination. *Acta Cryst.* **A71**, 3-8 (2015).

8 Sheldrick, G. M. Crystal structure refinement with *SHELXL*. *Acta Cryst* **C71**, 3-8 (2015).

9 Dolomanov, O. V., Bourhis, L. J., Gildea, R. J., Howard, J. A. K. & Puschmann, H. *OLEX2*: a complete structure solution, refinement and analysis program. *J. Appl. Cryst.* **42**, 339-341 (2009).

10 Groom, C. R., Bruno, I. J., Lightfoot, M. P. & Ward, S. C. The Cambridge Structural Database. *Acta Cryst.* **B72**, 171-179 (2016).

11 Kratzer, D. *FinalCif*, *V132*, https://dkratzert.de/finalcif.html.

12 Gaussian 16, Revision A.03, Frisch, M. J., Trucks, G. W., Schlegel, H. B., Scuseria, G. E., Robb, M. A., Cheeseman, J. R., Scalmani, G., Barone, V., Petersson, G. A., Nakatsuji, H., Li, X., Caricato, M., Marenich, A. V., Bloino, J., Janesko, B. G., Gomperts, R., Mennucci, B., Hratchian, H. P., Ortiz, J. V., Izmaylov, A. F., Sonnenberg, J. L., Williams-Young, D., Ding, F., Lipparini, F., Egidi, F., Goings, J., Peng, B., Petrone, A., Henderson, T., Ranasinghe, D., Zakrzewski, V. G., Gao, J., Rega, N., Zheng, G., Liang, W., Hada, M., Ehara, M., Toyota, K., Fukuda, R., Hasegawa, J., Ishida, M., Nakajima, T., Honda, Y., Kitao, O., Nakai, H., Vreven, T., Throssell, K., Montgomery, J. A., Jr., Peralta, J. E., Ogliaro, F., Bearpark, M. J., Heyd, J. J., Brothers, E. N., Kudin, K. N., Staroverov, V. N., Keith, T. A., Kobayashi, R., Normand, J., Raghavachari, K., Rendell, A. P., Burant, J. C., Iyengar, S. S., Tomasi, J., Cossi, M., Millam, J. M., Klene, M., Adamo, C., Cammi, R., Ochterski, J. W., Martin, R. L., Morokuma, K., Farkas, O., Foresman, J. B. & Fox, D. J. Gaussian, Inc., Wallingford CT, 2016.

13 Becke, A. D. Density-functional exchange-energy approximation with correct asymptotic behavior. *Phys. Rev. A* **38**, 3098-3100 (1998).

14 Perdew, J. P. Density-functional approximation for the correlation energy of the inhomogeneous electron gas. *Phys. Rev. B* **33**, 8822-8824 (1986).

15 Weigend, F. & Ahlrichs, R. Balanced basis sets of split valence, triple zeta valence and quadruple zeta valence quality for H to Rn: Design and assessment of accuracy. *Phys. Chem. Chem. Phys.* **7**, 3297-3305 (2005).

16 Grimme, S., Antony, J., Ehrlich, S. & Krieg, H. A consistent and accurate ab initio parametrization of density functional dispersion correction (DFT-D) for the 94 elements H−Pu. *J. Chem. Phys.* **132**, 154104 (2010).

17 Grimme, S., Ehrlich, S. & Goerigk, L. Effect of the damping function in dispersion corrected density functional theory. *J. Comp. Chem.* **32**, 1456-1465 (2011).

18 NBO 7.0, Glendening, E. D., Badenhoop, J. K., Reed, A. E., Carpenter, J. E., Bohmann, J. A., Morales, C. M., Karafiloglou, P., Landis, C. R. & Weinhold, F. Theoretical Chemistry Institute, University of Wisconsin, Madison, WI, 2018.
